# Global transcriptome profiling and functional analysis reveal that tissue-specific constitutive overexpression of cytochrome P450s confers tolerance to imidacloprid in palm weevils in date palm fields

**DOI:** 10.1186/s12864-019-5837-4

**Published:** 2019-05-31

**Authors:** Binu Antony, Jibin Johny, Mahmoud M. Abdelazim, Jernej Jakše, Mohammed Ali Al-Saleh, Arnab Pain

**Affiliations:** 1 Department of Plant Protection, College of Food and Agricultural Sciences, King Saud University, Chair of Date Palm Research, Riyadh, 11451 Saudi Arabia; 2 Biotechnical Faculty, Agronomy Department, University of Ljubljana, SI-1000 Ljubljana, Slovenia; 3 BESE Division, King Abdullah University of Science and Technology (KAUST), Thuwal, Jeddah, 23955-6900 Saudi Arabia

**Keywords:** Insecticide resistance, Red palm weevil, Date palm, Cytochrome P450-dependent monooxygenases, Constitutive overexpression, RNAi

## Abstract

**Background:**

Cytochrome P450-dependent monooxygenases (P450s), constituting one of the largest and oldest gene superfamilies found in many organisms from bacteria to humans, play a vital role in the detoxification and inactivation of endogenous toxic compounds. The use of various insecticides has increased over the last two decades, and insects have developed resistance to most of these compounds through the detoxifying function of P450s. In this study, we focused on the red palm weevil (RPW), *Rhynchophorus ferrugineus,* the most devastating pest of palm trees worldwide, and demonstrated through functional analysis that upregulation of P450 gene expression has evolved as an adaptation to insecticide stress arising from exposure to the neonicotinoid-class systematic insecticide imidacloprid.

**Results:**

Based on the RPW global transcriptome analysis, we identified 101 putative P450 genes, including 77 likely encoding protein coding genes with ubiquitous expression. A phylogenetic analysis revealed extensive functional and species-specific diversification of RPW P450s, indicating that multiple CYPs actively participated in the detoxification process. We identified highly conserved paralogs of insect P450s that likely play a role in the development of resistance to imidacloprid: *Drosophila Cyp6g1* (*CYP6345J1*) and *Bemisia tabaci CYP4C64* (*CYP4LE1*). We performed a toxicity bioassay and evaluated the induction of P450s, followed by the identification of overexpressed P450s, including *CYP9Z82*, *CYP6fra5, CYP6NR1*, *CYP6345J1* and *CYP4BD4*, which confer cross-resistance to imidacloprid. In addition, under imidacloprid insecticide stress in a date palm field, we observed increased expression of various P450 genes, with *CYP9Z82*, *CYP4BD4, CYP6NR1* and *CYP6345J1* being the most upregulated detoxification genes in RPWs. Expression profiling and cluster analysis revealed P450 genes with multiple patterns of induction and differential expression. Furthermore, we used RNA interference to knock down the overexpressed P450s, after which a toxicity bioassay and quantitative expression analysis revealed likely candidates involved in metabolic resistance against imidacloprid in RPW. Ingestion of double-stranded RNA (dsRNA) successfully knocked down the expression of *CYP9Z82, CYP6NR1* and *CYP345J1* and demonstrated that silencing of *CYP345J1* and *CYP6NR1* significantly decreased the survival rate of adult RPWs treated with imidacloprid, indicating that overexpression of these two P450s may play an important role in developing tolerance to imidacloprid in a date palm field.

**Conclusion:**

Our study provides useful background information on imidacloprid-specific induction and overexpression of P450s, which may enable the development of diagnostic tools/markers for monitoring the spread of insecticide resistant RPWs. The observed trend of increasing tolerance to imidacloprid in the date palm field therefore indicated that strategies for resistance management are urgently needed.

**Electronic supplementary material:**

The online version of this article (10.1186/s12864-019-5837-4) contains supplementary material, which is available to authorized users.

## Background

Approximately 700 species of insects cause serious damage to agricultural crops worldwide, both in the field and during storage [1]. It has been almost seven decades since the introduction of the new concept of synthetic organic insecticides for successful insect control, and current pesticide expenditures exceed $10.2 billion globally [2, 3]. However, the recent intensive and repeated use of certain insecticides has led to resistance in many insects worldwide. One of the ways insects evolve to cope with insecticides by invoking internal enzyme systems to break down and excrete these compounds [4–6]; these functions are achieved through elevated activity of detoxifying enzymes [7] and xenobiotic transporters [8], respectively. In general, there are three main types of detoxifying enzymes: cytochrome P450-dependent monooxygenases (P450s), general esterases (Ests) and glutathione S-transferases (GSTs); and ATP-binding cassette (ABC) transporters are also involved in the metabolic detoxification and excretion of insecticides [7, 8]. One characteristic of these enzyme systems is increased levels of activity resulting from constitutive overexpression in resistant insects [7, 9–13]. In addition to greater efficiency, these enzyme systems may exhibit a broad spectrum of activity, allowing them to degrade many different insecticides and plant toxins that are lethal to insects. Overall, increased activity and elevated expression of the Est, GST and P450 enzyme systems are associated with insecticide resistance [7], and studies conducted over the past two decades provide evidence that induction and constitutive overexpression are responsible for the detoxification of insecticides in resistant insects [5, 6, 9, 14–18]. For instance, constitutive overexpression of detoxifying enzymes is associated with neonicotinoid resistance in *Bemisia tabaci* [19, 20], *Nilaparvata lugens* [21, 22], *Leptinotarsa decemlineata* [23–26], *Tribolium castaneum* [27], *Laodelphax striatellus* [28] and *Bradysia odoriphaga* [29]. It has been hypothesized that the induction and constitutive overexpression of detoxification enzymes and their activities in insects are involved in the adaptation of insects to their environment, detoxification of insecticides and development of resistance [5, 9, 23]. Such resistance has serious consequences in agriculture practice, and some of the major concerns include significant yield losses and environmental contamination, which have been estimated to translate to $3–5 billion per year on average [30, 31].

P450s are a common route by which insects become resistant to insecticides [4, 9, 32]. P450s metabolize and detoxify insecticides through oxidative reactions; thus, constitutive upregulation of P450 genes is considered a marker of the development of insecticide resistance [5, 6, 32]. P450 activity is found in many insect tissues, such as the fat body, midgut, Malpighian tubules, nervous system and antennae, with the highest activity usually being associated with gut tissues and the fat body [33–35]. Although many studies have identified overexpression of multiple P450 genes in various insects upon neonicotinoid exposure [36–41], there have been few studies on the P450 mechanisms of insecticide resistance in insect pests of trees. Here, we present the P450 repertoire of the red palm weevil (RPW), *Rhynchophorus ferrugineus* (Olivier), which is the most successful pest of palm trees, and we compare P450 expression between field-caught imidacloprid-resistant, laboratory-induced and susceptible weevils. After these analyses, we selected the highly induced P450 transcripts, and functional analysis of these genes was performed through RNA interference (RNAi).

RPW is among the world’s most invasive pest species of palm trees. It is indigenous to South Asia and has spread rapidly during the last three decades, mostly due to the transport of infested planting material to Middle Eastern countries, Africa, and Europe [42–46]. In Middle Eastern countries, this pest has wreaked havoc during the last two decades, with annual losses in the Gulf region due to the eradication of severely infested palms estimated at US $8 million in 2010 [42]. Moreover, *R. ferrugineus* is the most destructive pest of date palms in Saudi Arabia, where the Agriculture Ministry launched a $31 million national campaign to fight the pest in January 2011. Although various methods of control are still practiced in many countries, insecticide treatment using “trunk injection techniques” provides satisfactory control for infected trees [42, 43]. In Middle Eastern countries, next-generation insecticides belonging to the neonicotinoid (imidacloprid) and phenylpyrazole (fipronil) groups have commonly been used during the last decade as prophylactic and curative applications against *R. ferrugineus* in date palms [42, 47–49]. Although insecticide treatment initially provided satisfactory results, recent studies have shown that RPW continues to attack date palm trees even after many insecticide applications [50]. In this study, we sought to determine the P450 mechanism underlying the connection between the induction of imidacloprid in *R. ferrugineus* and the identification of a resistant population in the field. We focused on the cytochrome P450 complement (CYPome) because many previous biochemical and physiological studies have demonstrated the involvement of P450-mediated detoxification in imidacloprid resistance in insects [19, 20, 23, 27, 51]. We investigated potential differences in the expression levels of P450s that might be associated with imidacloprid resistance. We then used RNA interference to knock-down the expression of overexpressed P450 genes in RPWs, and toxicity bioassays were conducted to evaluate the phenotypic effects of these genes on imidacloprid resistance mechanisms. We compared the gene expression pattern of silenced resistance-related P450s between field-caught resistant RPWs and laboratory-induced RPWs. Our analyses confirmed that constitutive overexpression of P450s likely plays a role in developing tolerance to imidacloprid in RPWs. The results of this study are expected to contribute to a better understanding of resistance in *R. ferrugineus* in Middle Eastern countries and to provide more insight into the candidate P450s that are directly involved in neonicotinoid detoxification in *R. ferrugineus.*

## Methods

### Insecticide

Imidacloprid (Confidor® 350 SC), which was purchased from Bayer Corporation (Riyadh, Saudi Arabia), is a suspension concentrate containing 350 g.a.i./L imidacloprid (a 35% concentration of the active ingredient, imidacloprid).

### Insect tissue collection and RNA extraction

The RPW collections were made with the direct permission of a cooperating landowner [Al-Kharj region (24.1500° N, 47.3000° E) of Saudi Arabia] in 2009; since then, the RPW culture has been maintained in our laboratory on sugarcane stems, as previously described [52]; this RPW strain is considered to be susceptible to imidacloprid. The tolerant strain of RPW was originally collected from a date palm orchard in the Al Qassim (25.8275° N, 42.8638° E) area, where farmers have continuously used imidacloprid-trunk-injection in last 10 years as a pest management strategy.

Total RNA was extracted from RPW fat body and gut tissues and previously reported whole body and antennae transcriptome data [53, 54] were screened and annotated for candidate P450 genes. For tissue specificity and reverse transcription polymerase chain reaction (RT-qPCR) analyses, samples of approximately 30 mg of tissue were collected from the antennae, proboscis, legs, wings, abdomen and thorax of adult males and females, and total RNA was isolated using the RNeasy Plus kit (Qiagen, USA) according to the manufacturer’s instructions. First-strand cDNA was synthesized from total RNA using SuperScript II Reverse Transcriptase (Invitrogen, USA) in accordance with the manufacturer’s instructions, and the quantity and quality of the total RNA and cDNA were validated using a NanoDrop spectrophotometer (Thermo Fisher Scientific, USA) and through PCR with the tubulin gene primer pair RferTubulin F/R (Additional file 3: Table S1) [46, 54, 55], respectively.

### Identification and classification of *R. ferrugineus* P450 genes

Each P450 transcript from the RPW transcriptome dataset [53, 54] was identified via local or web-based searches using the BLASTx and BLASTn programs of the National Center for Biotechnology Information (NCBI) [56]. A quality control step was first performed on the raw sequencing reads using the NGS QC Toolkit [30]. BLAST hits with e-values < 1.0e^− 5^ were considered to be significant [57], and transcripts were putatively assigned to each contig based on BLASTx hit score values. The BLAST XML files were uploaded to Blast2GO, followed by mapping, transcript annotation, INTERPRO and Kyoto Encyclopedia of Genes and Genomes (KEGG) analyses [58, 59]. Transcripts containing errors leading to incorrect assembly were edited using Geneious v7.1.5 [60], and de novo assembly of the P450 isotigs were performed. The open reading frame (ORF) finder tool (NCBI) was used to determine the ORF of each P450 contig. Reads per kilobase per million (RPKM) values were calculated for the assembled transcripts based on mapping data [61]. We reassembled both the antennal and whole-body transcriptome P450s using Geneious v7.1.5 to generate a reliable transcriptome dataset, and the cytochrome P450 genes and nomenclature were determined based on the guidelines provided by the Committee on Standardized Cytochrome P450 Nomenclature [62, 63], and P450 genes were named by Dr. David Nelson (CYP nomenclature committee). Both Blast2GO and manual annotations were performed based on the nomenclature, and the recommended identity cut-offs of 40% for the CYP family and 55% for the subfamily were strictly followed. The transcripts were categorized into four CYP clans: CYP3, CYP4, CYP2 and mitochondrial clans [14].

### Tissue-specific expression analysis

The tissue specificity of the P450 candidates was analyzed in various tissues collected from both male and female *R. ferrugineus*, including samples of the antennae, proboscis, thorax, abdomen, legs and wings. Total RNA extraction and cDNA synthesis were performed as described above. The gene-specific primers indicated in Additional file 3: Table S1 were used to amplify P450 genes, and tubulin and β-actin were used as internal controls [46, 55, 64, 65]. PCR amplification was performed using the following touchdown PCR program: 95 °C for 5 min, followed by 30 cycles of 95 °C for 1 min, 55 °C (±5 °C) for 30 s and 72 °C for 1 min, with a final cycle at 72 °C for 10 min. The PCR products were separated in 3% agarose gels and visualized with ethidium bromide staining.

### Phylogenetic analysis of RPW P450s

The sequence homology and evolutionary aspects of the RPW P450s were analyzed by reconstructing a phylogenetic tree using protein sequences from RPW and closely related beetles or insects. The annotated P450 sequences from the representative insect species *T. castaneum* (red flour beetle), *D. melanogaster* (fruit fly), *L. decemlineata* (Colorado potato beetle), *Agasicles hygrophila* (alligator weed flea beetle) and *Apis mellifera* (European honey bee) were obtained from the NCBI database and the cytochrome P450 monooxygenase homepage (http://drnelson.uthsc.edu/CytochromeP450.html). Multiple sequence alignments were performed using MAFFT v.7 [66], with the E-INS-i iterative refinement strategy and default parameters. The LG + I + G + F substitution model was determined as the best-fit models of protein evolution by ProtTest 3.4 [67]. Phylogenetic reconstruction and analysis of the CYP clade distribution were performed via the recommended maximum likelihood method, with the statistical bootstrap support of 1000 replications, using PhyML 3.0 [68].

### Relative expression analysis of P450s in the gut and fat body by RT-qPCR

cDNAs were prepared from RNA (~ 1 μg) extracted from the gut and fat body of 20-day-old insects, as noted above. The RT-qPCR experiments were performed according to the Minimum Information Required for Publication of Quantitative Real-Time PCR Experiments (MIQE) Guidelines [69, 70]. RT-qPCR was carried out using SYBR Green PCR Master Mix (Life Technologies, USA) with three biological and three technical replicates, according to the manufacturer’s instructions. The oligonucleotide primers for these analyses were the same as those used in the tissue-specific studies, and tubulin and β-actin (Additional file 3: Table S1) were employed to normalize gene expression. Relative P450 expression levels were measured via the 2^−ΔC^_T_ method [71]. The following thermal cycling program was used to perform PCR amplification: 50 °C for 20 s (precycling), then 95 °C for 10 min (holding), followed by 40 cycles of 95 °C for 15 s and 55 °C for 40 s; then, a melting curve analysis stage consisting of 95 °C for 15 s, 60 °C for 1 min, 95 °C for 30 s and 60 °C for 15 s.. The RT-qPCR products were examined through 3% agarose gel electrophoresis and visualized via ethidium bromide staining.

### Toxicity bioassay - induction of P450s using imidacloprid

Laboratory trials were conducted using a susceptible strain of RPW and field-caught RPWs (21-day-old males and females at a ratio of 1:1) to evaluate the efficiency of insecticide dilutions using a bioassay technique in which food is dipped into the insecticide [49, 72]. The numbers of moribund or dead adults were recorded daily, and moribund adults were counted as dead adults. Mortality was recorded for a period of 11 days. A preliminary laboratory toxicity bioassay was performed to identify a range of concentrations to be used for determining the LC_50_ values of imidacloprid. Based on the preliminary laboratory toxicity bioassay of Confidor® 350 SC, concentrations of 1, 2, 4, 6 and 8 mM (250, 500, 1000, 1500 and 2000 ppm, respectively), were prepared using dH_2_O; sugarcane stems treated with dH_2_O were used as the control. For the determination of LC_50_ values, the bioassay results were analyzed via probit analysis using the Ldp Line program (http://www.ehabsoft.com/ldpline/). Mortality was corrected using Abbott’s formula for each probit analysis. In this study, we considered 33% of the median lethal value as the sublethal concentration. Biological triplicate samples of RPWs of both the susceptible strain (to obtain an induced strain, hereafter referred to as *ind*) and field-caught strains (hereafter, *res*) were exposed to sublethal concentrations of imidacloprid for 11 days. Adults of the susceptible strain of RPW were treated with distilled water to obtain a negative control (hereafter, *sus*). After 11 days, *sus, ind* and *res* weevils were dissected, and the gut and fat body tissues were removed, placed in ice-cold phosphate-buffered saline (PBS) and stored in RNA*later*® solution (Ambion, USA). Total RNA was extracted, and cDNA was synthesized as described above.

### Overexpression profiling of P450s in *sus*, *ind* and *res* populations of *R. ferrugineus*

Expression profiling of P450s induced in response to imidacloprid was evaluated by comparing P450 expression in *sus*, *ind* and *res* samples via quantitative real-time PCR (RT-qPCR). Tissues from the major detoxification sites (i.e., the gut and fat body) were removed from the three samples and stored in RNA*later*® solution (Ambion, USA) prior to RNA extraction. RNA extraction and cDNA synthesis were conducted as described above, and RT-qPCR was performed with the same primers used for the tissue specificity analysis (Additional file 3: Table S1) with *Power* SYBR® Green PCR Master Mix (Life Technologies, USA). Of the 77 P450 genes identified in this study, 50 were selected for RT-qPCR expression profiling based on their tissue-specific expression patterns, RPKM values and available support in the literature. Twenty-seven P450s were discarded based on previous analyses and published evidence of a lack of expression in relevant tissues. From literature-supported important CYP families, specifically CYP6, CYP4, CYP9 and CYP345, a total of 50 P450s from *R. ferrugineus* were selected for RT-qPCR analysis using cDNA from the *sus*, *res* and *ind* populations of RPWs (Additional file 3: Table S1). Thermal cycling was conducted using a 7500 Fast Real-Time PCR system (Life Technologies, USA) at 50 °C for 20 s (precycling), then 95 °C for 10 min (holding), followed by 40 cycles of 95 °C for 15 s and 55 °C for 35 s; then, a melting curve analysis stage consisting of 95 °C for 15 s, 60 °C for 1 min, 95 °C for 30 s and 60 °C for 15 s was conducted. The reactions were performed in triplicate with three biological replicates for each tissue type/sample, and relative quantification of gene expression in each target tissue was calculated using the comparative 2^-ΔΔCт^ method [73] in comparison with the same tissues from *sus* insects. The RPW tubulin and β-actin genes (Additional file 3: Table S1) were used as endogenous controls [46, 54, 55].

#### Cluster analysis, expression profiling and statistical analysis

Hierarchical cluster analysis was performed on the RT-qPCR data using Cluster software [74]. The 2^-ΔCт^ values from the RT-qPCR analysis were log transformed, and data from each sample and tissue were clustered on the basis of the average linkage distance between the median values [71, 74]. The significance of differences in P450 transcript expression were calculated between *ind* and *res* samples compared to *sus* using the paired t-test, with an alpha significance level of 0.05 (*p* < 0.05), using SPSS (v24). Multiple-comparison testing with the least significant difference (LSD) test was performed to assess the differential expression of transcripts within each CYP family (*p* < 0.05) using SPSS (*v*24).

### P450 silencing through RNA interference (RNAi) and toxicity bioassays

The three most important and differentially expressed P450 genes (*CYP9Z82*, *CYP345J1* and *CYP6NR1*) were selected for RNAi experiments. We selected *CYP9Z82*, *CYP6NR1* and *CYP345J1* for the RNAi experiments because the first two P450s were highly expressed and differentially expressed, respectively, and the remaining P450 was a paralog of *Drosophila Cyp6g1* [41, 75, 76] and *B. tabaci CYP4C64* [19], which confer cross-resistance to imidacloprid. The PCR amplification reactions were carried out as follows: 95 °C for 5 min, followed by 30 cycles of 95 °C for 1 min, 56 °C for 30 s and 72 °C for 2 min and, finally, one cycle at 72 °C for 10 min.

The full-length amplified PCR products within the expected size range were gel purified with the Wizard® SV Gel and PCR Clean-Up System (Promega, WI, USA), followed by ligation into the pGem-T Easy vector (Promega) and transformation into the JM109 *Escherichia coli* system. The plasmid products were isolated and sequenced with an ABI 3500 genetic analyzer (Life Technologies) using vector primers M13-F/R (Additional file 3: Table S1). We used plasmids containing the full-length P450s ORF as template DNA to synthesize double-stranded RNA (dsRNA). The vector primer, M13-F and reverse primers targeting the ORFs with a T7 overhang (Additional file 3: Table S1) were used to amplify and linearize the ORFs, which were rechecked via direct sequencing (ABI 3500, Life Technologies, USA). dsRNA synthesis was performed using the MEGAscript RNAi Kit (Life Technologies, USA) according to the manufacturer’s instructions, and the results were quantified using a NanoDrop 2000 (Thermo Scientific, DE, USA). The integrity and efficiency of dsRNA duplex formation were examined through 1% agarose gel electrophoresis. The RNAi experimental design was set up into three separate controls which were universal negative dsRNA control (Integrated DNA Technologies, Leuven, Belgium) (hereafter, negative control), no-injection (hereafter, NI) and nuclease free water-injected (hereafter, NFW) group. We selected 10-day-old *R. ferrugineus* pupae for the RNAi experiments, and 100 ng/μL dsRNA (in 20 μL) was injected, after which the dsRNA-injected and RPW pupae were maintained as previously described [55, 64]. The emerged adults were transferred to a separate box containing a piece of fresh sugarcane, and after 7 days were further subjected to toxicity assays using sublethal concentrations of imidacloprid (~ 1.1 mM) for 11 days. Seven days after injection, cDNAs prepared from RNA extracted from the fat body of each individual insect and the gene expression pattern of RPW adults test sample (*CYP9Z82*, *CYP345J1* and *CYP6NR1*) was compared with that of dsRNA control, NI and NFW weevils to obtain the percentage knockdown of each P450 gene. RT-qPCR reactions were carried out using SYBR Green PCR Master Mix (Life Technologies, USA) according to the manufacturer’s instructions, with three biological and three technical replicates. Eleven days after toxicity assays, all surviving adults were exposed to a temperature of − 20 °C until completely immobilized (approximately 10 min), at which time RNA extraction and cDNA synthesis was carried out as described below, from the gut and fat body tissues for each experimental group. Thereafter, an analysis of phenotypic effects and comparison of P450 gene expression pattern between silenced weevils and *res* and *ind* RPWs were carried out as described above. The toxicity assays were carried out with two separate controls, P450 dsRNA-injected RPW adults were treated with nuclease-free water (hereafter, *n-trt*) or were left uninjected (same as *sus*). P450 dsRNA-injected RPW adults were subjected to toxicity assays (hereafter, *trt*) and were compared with the *sus*, *ind* and *res* groups to determine the gene-silencing effect on CYP gene induction and constitutive overexpression.

### Phenotypic effects and gene silencing validation via RT-qPCR

During the toxicity assay, conducted over a period of 11 days, the adult weevil survival rate was monitored daily, and Kaplan-Meier survival analysis with the log rank test was performed using SPSS (v24) to determine the survival function in the treated and control groups. cDNAs prepared from RNA extracted from the gut and fat body of each individual insect (surviving) in the experimental (*n-trt* and survived adult-*trt*) and *sus*, *ind* and *res* RPW groups were also used as templates for RT-qPCR. Reactions were carried out using SYBR Green PCR Master Mix (Life Technologies, USA) according to the manufacturer’s instructions, with six biological and three technical replicates. *Tubulin* and *β-actin* primers were used to normalize gene expression (Additional file 3: Table S1). The relative expression levels of P450s in the silenced vs. control groups were measured via the 2^−ΔΔC^_T_ method [71, 73]. PCR amplification, data analysis and gel evaluation were performed as described above. Difference analysis was performed by using Student’s t test, followed by Duncan’s multiple comparison test, with SPSS (*v*24) software. A value of *p* < 0.05 was considered significant.

## Results

### Identification of putative RPW P450 candidates

The RPW transcriptome dataset sequence assembly and annotation are summarized in Additional file 4: Table S2. Our extensive search for P450s in the RPW antennal and whole-body transcriptome datasets resulted in the identification of 101 putative candidates, which were further screened for duplications through a BLASTx homology search within the Blast2GO annotation program (Additional file 5: Table S3). The 24 removed transcripts included hypothetical proteins as well as repeated transcripts from the antenna and whole-body databases. After combined assembly of the antennal and whole-body transcriptome datasets of *R. ferrugineus*, we obtained 77 transcripts encoding P450s (Additional file 5: Table S3). The adopted nomenclature was according to the guidelines provided by the Committee on Standardized Cytochrome P450 Nomenclature [63] (Additional file 5: Table S3), and the transcripts were categorized into four CYP clans, namely, the CYP3, CYP4, CYP2 and mitochondrial clans [14, 62, 63, 77, 78]. The official nomenclature obtained from Dr. David R. Nelson (CYP nomenclature committee) is presented in Additional file 5: Table S3. The number of *R. ferrugineus* representatives in each P450 clan and the BLASTx results used for nomenclature are provided in Table 1 and Additional file 5: Table S3, respectively. A total of 16 CYP families were identified, including CYP6 (42.8%) and CYP4 (23%) within the total identified CYPs. The CYP9 family and mitochondrial clan accounted for only 10% of the total *R. ferrugineus* CYPs (Additional file 5: Table S3). The largest families included CYP6, with 33 genes, followed by CYP4, with 14 genes (Table 1). The CYP2 clan contained fewer genes and exhibited less evolutionary differentiation than the other clans.

**Table 1:** Numbers of genes in P450 clans and families identified in *R. ferrugineus*

P450 Clan	Families	Subfamilies	Total number of genes
CYP2	3 (CYP18, 305, 306)	3	3
CYP3	5 (CYP6, 9, 324, 345, 347)	20	43
CYP4	8 (CYP4, 341, 433, 412, 15, 326, 349, 410)	16	23
Mitochondrial	6 (CYP12, 49, 301, 314, 315, 334)	6	8
Total	22	45	77

### P450 phylogenetic analysis

Phylogenetic analysis was conducted based on amino acid sequences using *R. ferrugineus* and four other insects (*T. castaneum*, *L. decemlineata*, *A. hygrophila, B. tabaci* and *Drosophila melanogaster*) [20, 23, 75, 76, 79] to identify gene orthologs and paralogs. RPW contains highly expanded CYP3 (5 families, 43 individual genes) and CYP4 (8 families, 23 individual genes) clans, most notably in family 4, family 6 and family 9 (Table 1). These results show that there is a high frequency of species-specific CYP genes in the CYP4 and CYP6 family, as reported in other coleopterans, *T. castaneum* [79] and *L. decemlineata* [23]. We selected a best fit-model of P450 protein evolution, and maximum-likelihood trees were constructed for CYP2, CYP3, CYP4 and mitochondrial clans.

**Fig. 1:**
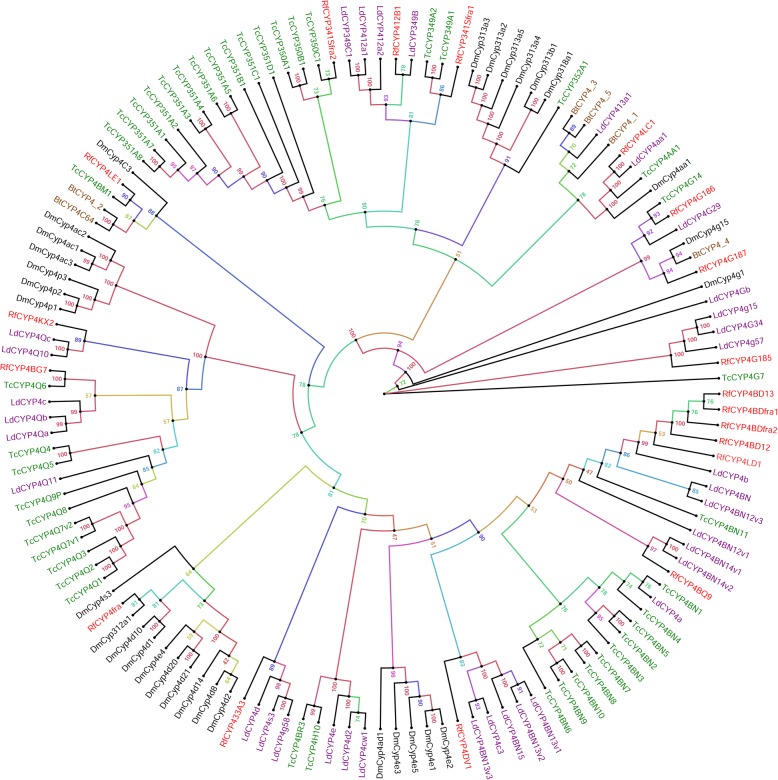
Maximum likelihood consensus tree of CYP4 clan. CYP4 clan sequences from *Tribolium castaneum, Bemisia tabaci*, *Leptinotarsa decemlineata* and *Drosophila melanogaster* P450s were used as references to classify *R. ferrugineus* CYP4 (red). Multiple sequence alignments were performed using MAFFT v.7 with the E-INS-i iterative refinement strategy and default parameters, and the LG + I + G + F substitution model was determined as the best-fit model of protein evolution by ProtTest 3. The maximum likelihood (ML) analysis was computed using PhyML 3.0, with 1000 bootstrap replications and the bootstrap values are indicated at the nodes. P450s from different species were marked with different colors. The phylogenetic tree was visualized using FigTree (http://tree.bio.ed.ac.uk/software/figtree/) and branch appearance was colored based on the bootstrap values. Scale = 0.5 amino acid substitutions per site

In *R. ferrugineus* CYP4 clan*,* 8 families (CYP4, CYP341, CYP433, CYP412, CYP15, CYP326, CYP349 and CYP410) and 16 subfamilies were found (Table 1, Fig. 1). Members of the CYP4 clan show wide diversity in both their sequences and functions (Fig. 1). In Clan 4, CYP4 family is the largest gene family and we identified CYP4G, CYP4BD, CYP4Q, CYP4AA, CYP4LE, CYP4KX, CYP4BQ and CYP4D (Fig. 1). Most RPW P450s in the CYP4 clan had gene orthologs in *L. decemlineata* and *T. castaneum*, or exhibited gene expansions with close phylogenetic relationships. Gene expansions were noticed within the CYP4 clan, particularly the cluster of CYP4BD (Fig. 1). *R. ferrugineus* CYP4 tree also revealed that CYP4G clade belongs to a group containing CYP4G isoforms from *T. castaneum*, *L. decemlineata*, *D. melanogaster* and *B. tabaci*, with > 90% bootstrap support (Fig. 1). Except in CYP433 and CYP4KX, all other CYP genes in *R. ferrugineus* was clustered with their related genes in other insects. Interestingly, *CYP4Q6* and *CYP4BQ9*from *R. ferrugineus* were found to be clustered with *L. decemlineata LdCYP4Qb* and *LdCYP4e*, respectively*,* which has been reported to be more than five-fold overexpressed in imidacloprid resistance (Fig. 1) [23]. The phylogenetic analysis identified an ortholog of *L. decemlineata* (*CYP4Qc*/*CYP4Q10*) that is involved in imidacloprid resistance [23] in *R. ferrugineus*. Similarly, the phylogenetic analysis identified a paralog of *B. tabaci CYP4C64* (GenBank JX144366) that is involved in imidacloprid resistance [20] in *R. ferrugineus* (*CYP4LE1*), with 67% bootstrap support (Fig. 1). In the *L. decemlineata* CYP4Q subfamily, two genes (*CYP4Qc* and *CYP4Qb*) are overexpressed upon imidacloprid induction [23], and we identified two candidates, *RferCYP4Q6* and *RferCYP4KX2*, that were similar in *R. ferrugineus* (Fig. 1). The *CYP4BD* subfamily exhibited the greatest increase in diversity compared with other insects, possibly reflecting adaptation to environmental xenobiotics.

**Fig. 2:**
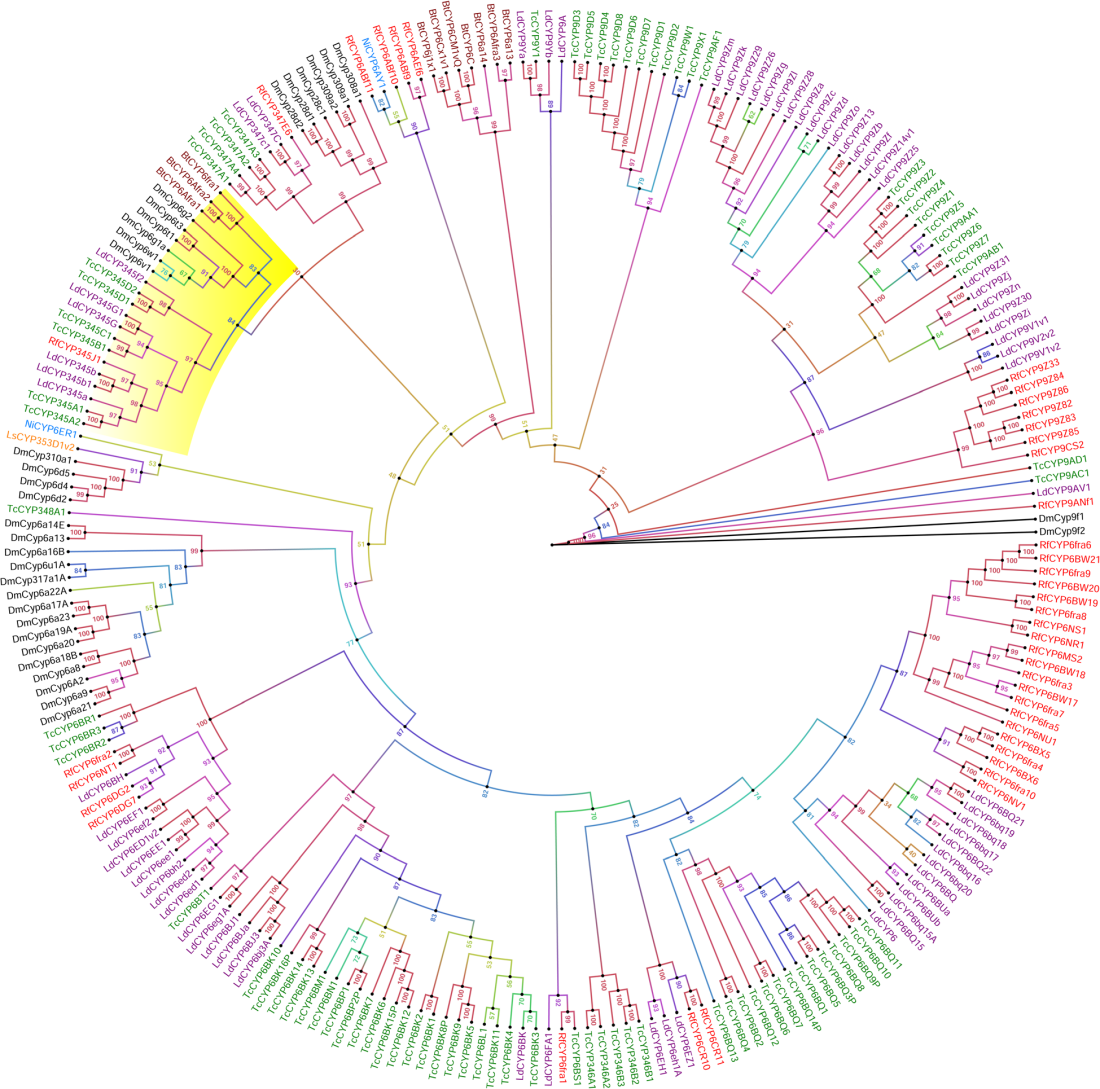
Maximum likelihood consensus tree of CYP3 clan. The phylogenetic tree reconstruction was performed using the same methods mentioned in Fig. 1, with LG + I + G + F substitution model determined as the best-fit model of protein evolution. P450s from *Tribolium castaneum, Bemisia tabaci*, *Leptinotarsa decemlineata* and *Drosophila melanogaster* P450s were used in the analysis to classify *R. ferrugineus* CYP3 clan. Additionally, Clan 3 candidates with reported imidacloprid resistance were added from *Laodelphax striatellus* and *Nilaparvata lugens*. P450s from different species were marked with different colors. The CYP6g1 clade is highlighted in yellow. The phylogenetic tree was visualized using FigTree (http://tree.bio.ed.ac.uk/software/figtree/) and branch appearance was colored based on the bootstrap values. Scale = 0.5 amino acid substitutions per site

In *R. ferrugineus*, we identified four CYP families (CYP6, 9, 324, 345, 347) and 20 subfamilies in the CYP3 clan and the CYP6 family is the largest family that has 35 individual genes. *R. ferrugineus* CYP6 family had 13 subfamilies, including *CYP6AB, CYP6AE, CYP6BW17, CYP6BX5, CYP6CR10, CYP6DG2, CYP6HY3, CYP6MS2, CYP6NR1, CYP6NS1, CYP6NT1, CYP6NU1* and *CYP6NV1*. Genes in the CYP6 -family showed multiple expansions in *R. ferrugineus,* resulting in species-specific clusters and beetle-specific clusters (Fig. 2). During the construction of the phylogenetic tree, we used CYP3 clan from *T. castaneum* and *L. decemlineata* to identify beetle-specific expansion of CYP6 genes, with *D. melanogaster* as an outgroup. These results suggest that lineage-specific expansions of CYPs have occurred in Coleoptera and Diptera [6] (Fig. 2). Significant species-specific expansion and divergence may also have occurred in *R. ferrugineus*, particularly in the clusters containing *CYP6BW*, *CYP6AB*, *CYP6BX,* and *CYP6DG* (Additional file 5: Table S3; Fig. 2). The *CYP6BW* subfamily showed the greatest increase in diversity compared with other insects, possibly reflecting adaptation to environmental xenobiotics. The CYP6 genes *CYP6EH1 CYP6FA1* and *CYP6BH* from *L. decemlineata* have been reported to be overexpressed in the presence of imidacloprid [23], and we identified orthologs in *R. ferrugineus* (*CYP6CR, CYP6fra1* and *CYP6DG*, respectively), with sound bootstrap support (> 90%) (Fig. 2). Similarly, as shown in Fig. 2, one CYP6 gene from *L. decemlineata*, *CYP6EZ1* [23], which is associated with imidacloprid resistance, forms a clade with two *R. ferrugineus* CYP6 candidates, *CYP6CR10* and *CYP6CR11*, with 90% bootstrap support. Phylogenetic analysis identified a paralog of *D. melanogaster CYP6g1* (GenBank: AAF58557.1)*,* a gene with an established association with imidacloprid resistance [80], in *R. ferrugineus* (*CYP345J1*); this gene is also similar to *T. castaneum CYP354A1* (GenBank: EFA12856.1) [27] (Fig. 2). The CYP6 gene *CYP6AY1* from *Nilaparvata lugens* [22] has been reported to be overexpressed in the presence of imidacloprid, and we identified a paralog in *R. ferrugineus* (*CYP6ABf11*), with 82% bootstrap support, also forms a clade with *R. ferrugineus CYP6ABf6, CYP6ABf9*, and *CYP6ABf10*. Close to this clade is represented by a highly expressed *CYP6CM1vQ* from *B. tabaci* (51% bootstrap support)*,* which is associated with imidacloprid resistance (GenBank: ACA51846.1) [19] (Fig. 2). All CYP354 group together in one cluster, with 84% bootstrap support (Fig. 2). This gene appears to undergo expansion by gene duplication forming allelic variants with novel functions.

In the clan 3, CYP9 family was the second biggest in *R. ferrugineus* that consisted of three subfamilies *CY9Z, CYP9CS* and *CYP9AN*. The phylogenetic analysis of CYP9 identified two major clades: one containing *CYP9Z* subfamily genes from *L. decemlineata, T. castaneum* and *R. ferrugineus* (96% bootstrap support); and a second containing CYP9A subfamily genes from *T. castaneum, L. decemlineata, R. ferrugnieus* and *D. melanogaster* (100% bootstrap support)*,* with a high degree of species-specific expansion (Fig. 2). Among them, all *CYP9Z* group together in one cluster, also includes CYP9CS with 99% bootstrap support (Fig. 2). The CYP9 families are relatively highly conserved across insect species and show diversity in terms of both sequence and function.

**Fig. 3:**
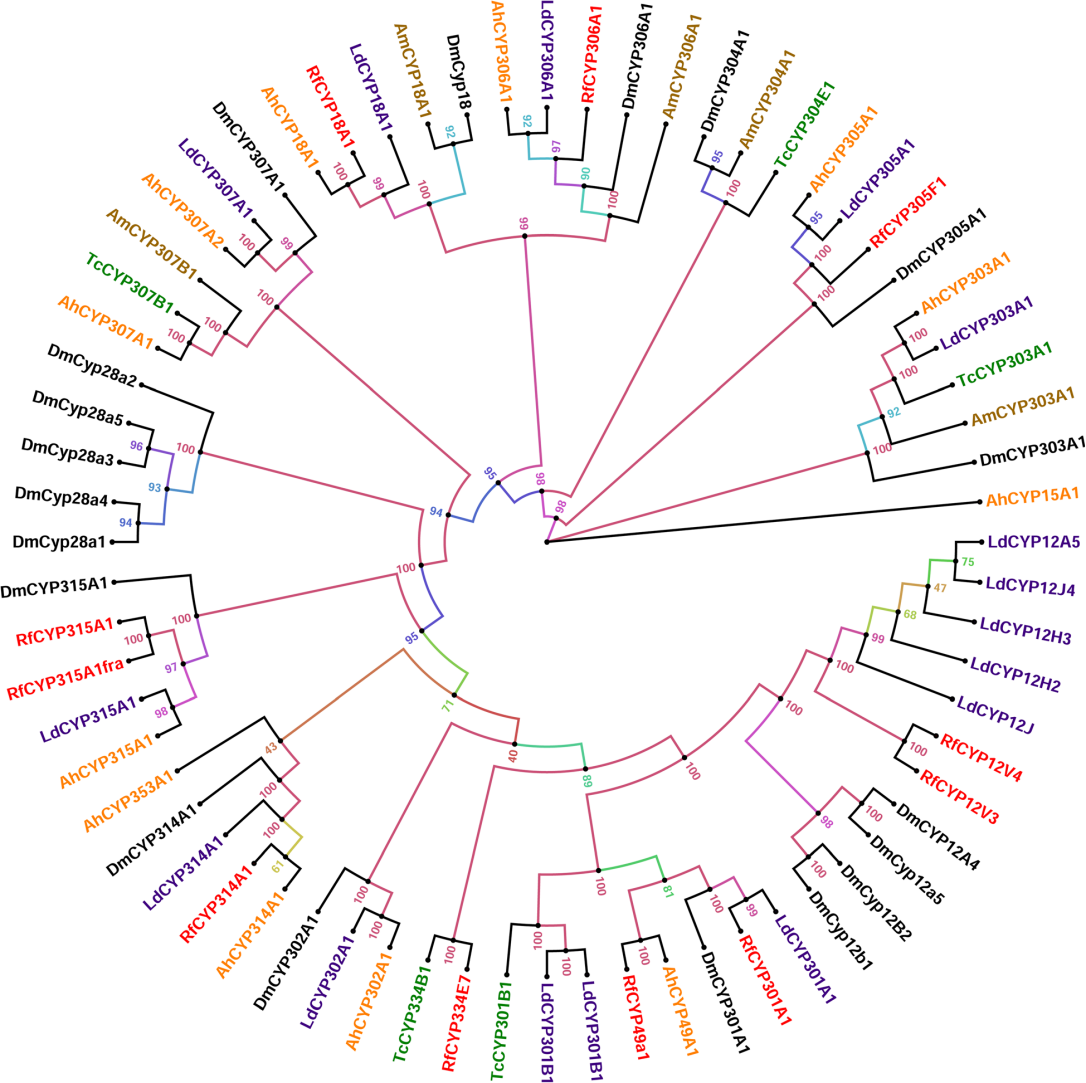
Maximum likelihood consensus tree of CYP2 and Mitochondrial clans. The phylogenetic tree reconstruction was performed using the same methods mentioned in Fig. 1, with LG + I + G + F substitution model determined as the best-fit model of protein evolution. P450s from *Tribolium castaneum*, *Bemisia tabaci*, *Leptinotarsa decemlineata* and *Drosophila melanogaster* P450s were used in the analysis to classify *R. ferrugineus* CYP2 and Mitochondrial clans. Additionally, candidates from *Agasicles hygrophila* and *Apis mellifera* were added for classification purposes. P450s from different species were marked with different colors. The phylogenetic tree was visualized using FigTree (http://tree.bio.ed.ac.uk/software/figtree/) and branch appearance was colored based on the bootstrap values. Scale = 2.0 amino acid substitutions per site

Phylogenetic analyses was performed for the *R. ferrugineus* mitochondrial clan and clan 2 with P450s identified in *T. castaneum*, *D. melanogaster*, *L. decemlineata, A. hygrophila* and *Apis mellifera*. *R. ferrugineus* CYP2 and mitochondrial clans showed a high degree of 1:1 orthology with those from other insect species. Within the clan 2, CYP303 A1showed high level of 1:1:1:1 orthologies. Three P450s were found in clan 2, belonging to CYP18, CYP305 and CYP306 families (Fig. 3). Of these, CYP306A1 was encoded by the *Phantom* gene from other insects, showed high sequence similarities with. *T. castaneum* and *A. hygrophila* (Fig. 3). In the clan 2, CYP18, CYP305 and CYP306 families, the tree branching showed that all coleopterans, *R. ferrugineus*, *T. castaneum*, *L. decemlineata* and *A. hygrophila* were grouped together and evolutionally separated from *A. mellifera* and *D. melanogaster* (Fig. 3). The major difference between the *R. ferrugineus* and *T. castaneum* in the clan 2 was possible loss of the CYP303, CYP304, CYP307 and CYP15 paralogs or orthologs in the *R. ferrugineus*.

In the mitochondrial clan in *R. ferrugineus*, eight P450s were found in six families of CYP12, CYP49, CYP301, CYP314, CYP315 and CYP334, and P450s, CYP314A1 and CYP315A1, encode orthologs of the Halloween genes *Shade* (*shd*) and *Shadow* (*sad*) [81], respectively from other insect species (Additional file 5: Table S3). In the mitochondrial clan, *R. ferrugineus* P450s were closely related to the coleopteran clade, namely CYP12, CYP301, CYP314 and CYP315 (Fig. 3). The major difference between the *R. ferrugineus* and *T. castaneum* in the mitochondrial clan was possible loss of the CYP302 and CY353 paralogs or orthologs in the *R. ferrugineus*. *R. ferrugineus* CYP301, CYP314 and CYP315 shared high sequence similarities with corresponding proteins in *D. melanogaster*, *A. mellifera*, *T. castaneum*, *L. decemlineata* and *A. hygrophila*.

### Tissue-specificity analysis

**Fig. 4:**
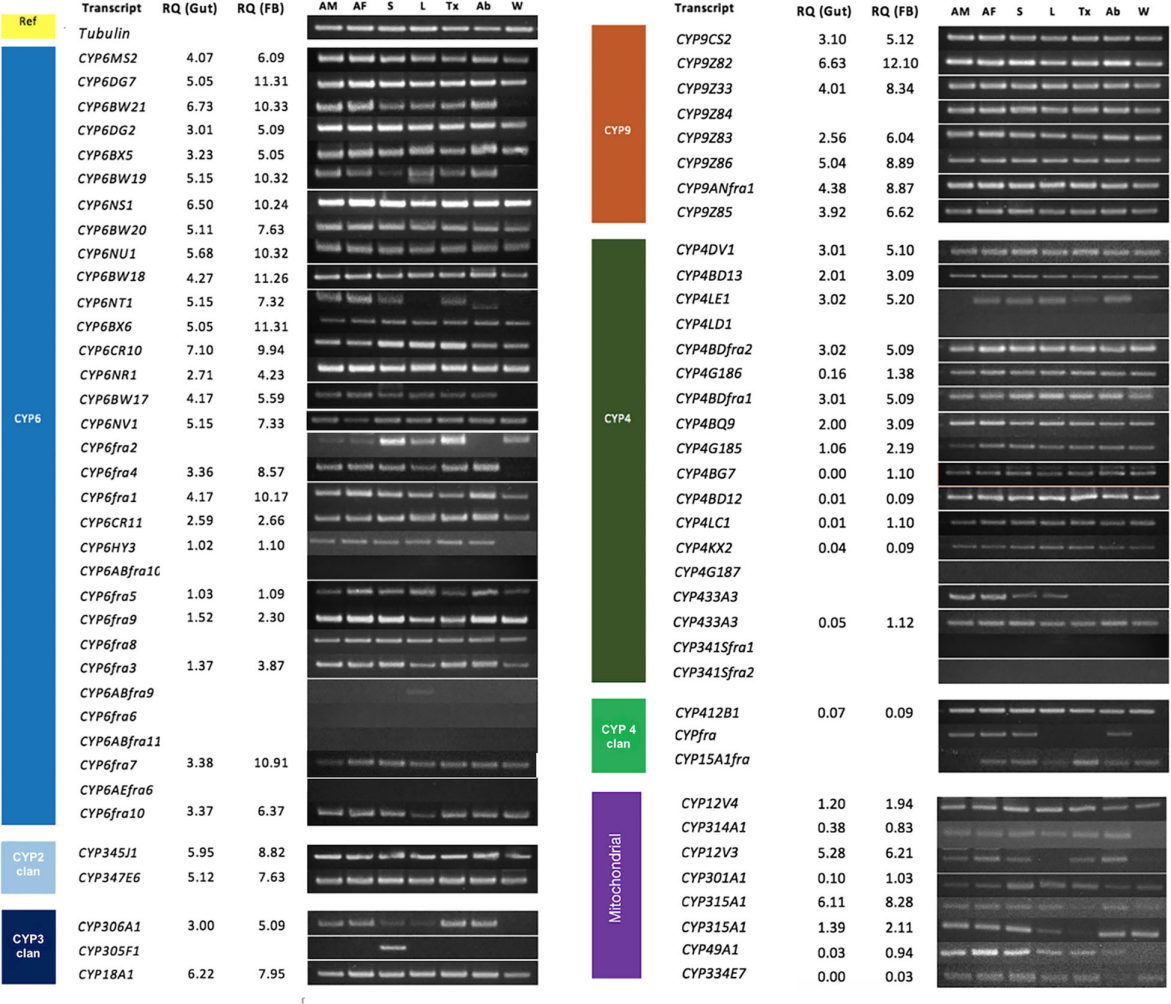
Relative tissue-specific expression of P450 transcripts, as analyzed via PCR and visualized in 3% agarose gels. Antenna Male (AM), Antenna Female (AF), Proboscis (S), Legs (L), Thorax (Tx), Abdomen (Ab) and Wings (W) indicate the source tissues. The expression of all P450s in the gut and fat body (FB) tissues was quantified via RT-qPCR, and the mean fold-changes in gene expression compared to *tubulin* and *β-actin* are provided under RQ. The oligonucleotide primer details, annealing temperature and amplification size (bp) are given in Additional file 1: Table S1. The 3 kb DNA ladder was used as a marker

The tissue-specific expression analysis indicated that most P450s are expressed in multiple tissues, with notable exceptions such as leg-specific *CYP6ABfra9* and proboscis-specific *CYP305F1* (Fig. 4). *CYP306A1* showed high expression levels in antenna, abdomen and thorax tissues. *CYP433A3* was found to be expressed exclusively in tissues such as the antenna, proboscis and thorax, and *CYP4fra*, from clade 4, showed localized expression in the antenna, proboscis and abdomen (Fig. 4). In contrast, low expression of *CYP6fra2* was observed in antennal tissues, and notably, a few transcripts with otherwise ubiquitous expression were absent from the wings (Fig. 4). The mitochondrial P450s were ubiquitously expressed in all tissues analyzed, except for *CYP12V3*, which was not expressed in the legs and wings (Fig. 4).

### Relative expression analysis of P450s in gut and fat body tissues

The expression of all P450s in the *sus R. ferrugineus* gut and fat body tissues was quantitatively measured, and the relative quantification (RQ) values are provided in Fig. 4. Based on the RQ data, *CYP9Z82*, *CYP6BX6*, *CYP6DG7*, *CYP6BW18*, *CYP6fra7*, *CYP6BW21*, *CYP6NU1* and *CYP6BW19* are highly expressed P450s in the fat body of *R. ferrugineus*. Compared to all CYPs, *CYPZ82*, *CYP6DG7* and *CYP6BX6* were found to be highly expressed in the fat body (Fig. 4). Other candidate genes showing high expression in the gut were *CYP6CR10*, *CYP6BW21*, *CYP9Z82*, and *CYP18A1*. One proboscis-specific candidate gene also displayed moderate expression (mean-fold-change normalized by tubulin and β-actin gene expression), as shown in Fig. 4.

### Toxicity bioassay: induction of P450s using imidacloprid

The probit analysis of different Confidor® 350 SC treatments (1–8 mM) against susceptible and field-caught adult RPWs over 4 days of exposure is presented in Additional file 6: Table S4. The action of imidacloprid against RPWs was comparatively rapid at the 8 mM concentration, where 75% mortality was observed for the susceptible RPWs within 1 d, whereas 41.67% mortality was observed for the field-caught adult RPWs. In susceptible RPWs, 100% mortality was observed within 4 d at the 8 mM concentration, whereas field-caught RPWs exhibited 66.67% mortality. In susceptible RPWs, probit analysis identified 3.44 mM as the LC_50_ value for 4 days of exposure (Additional file 6: Table S4), whereas in field-caught RPWs, 5.97 mM was identified as the LC_50_ value for 4 days of exposure (Additional file 6: Table S4). Thus, we selected ~ 1.14 mM, which represents 33% of the median lethal value, as the sublethal concentration for subsequent experiments. Biological triplicate samples of RPWs of both susceptible and field-caught strains (to obtain *res*) were exposed to an ~ 1.14 mM concentration of imidacloprid for 11 days to obtain induced (*ind*) and *res* strains, respectively. The susceptible strain of RPW adults was treated with double distilled water (hereafter, *sus*) for 11 days to obtain a negative control. No RPWs died during the experimental period. After 11 days, the adult weevils were classified as *sus* (negative control)*, ind* (induced) and *res* (positive control); the tissues were dissected; and CYP expression profiling was carried out.

### Overexpression profiling of P450s in *sus, ind* and *res* populations of *R. ferrugineus*

To identify P450s that might be induced in the *sus, ind* and *res* populations, we generated a relative expression profile of 50 P450s belonging to the CYP4, CYP6, CYP9 families and CYP2 clan. Candidates were selected based on tissue-specific expression profiles and RPKM and RQ values. Nine P450 candidates showing no expression in abdomen tissues (Fig. 4) and eight candidates that had not been previously associated with insecticide resistance, such as *CYP349* (2), *CYP341* (2), *CYP305* (2) *CYP410* (1) and *CYP* (1), were excluded (Additional file 5: Table S3). The induction of CYP genes was validated via quantitative PCR (RT-qPCR) on independent biological samples. The results showed a differential P450 induction pattern in gut and fat body tissues.

#### Induction in gut tissues

**Fig. 5:**
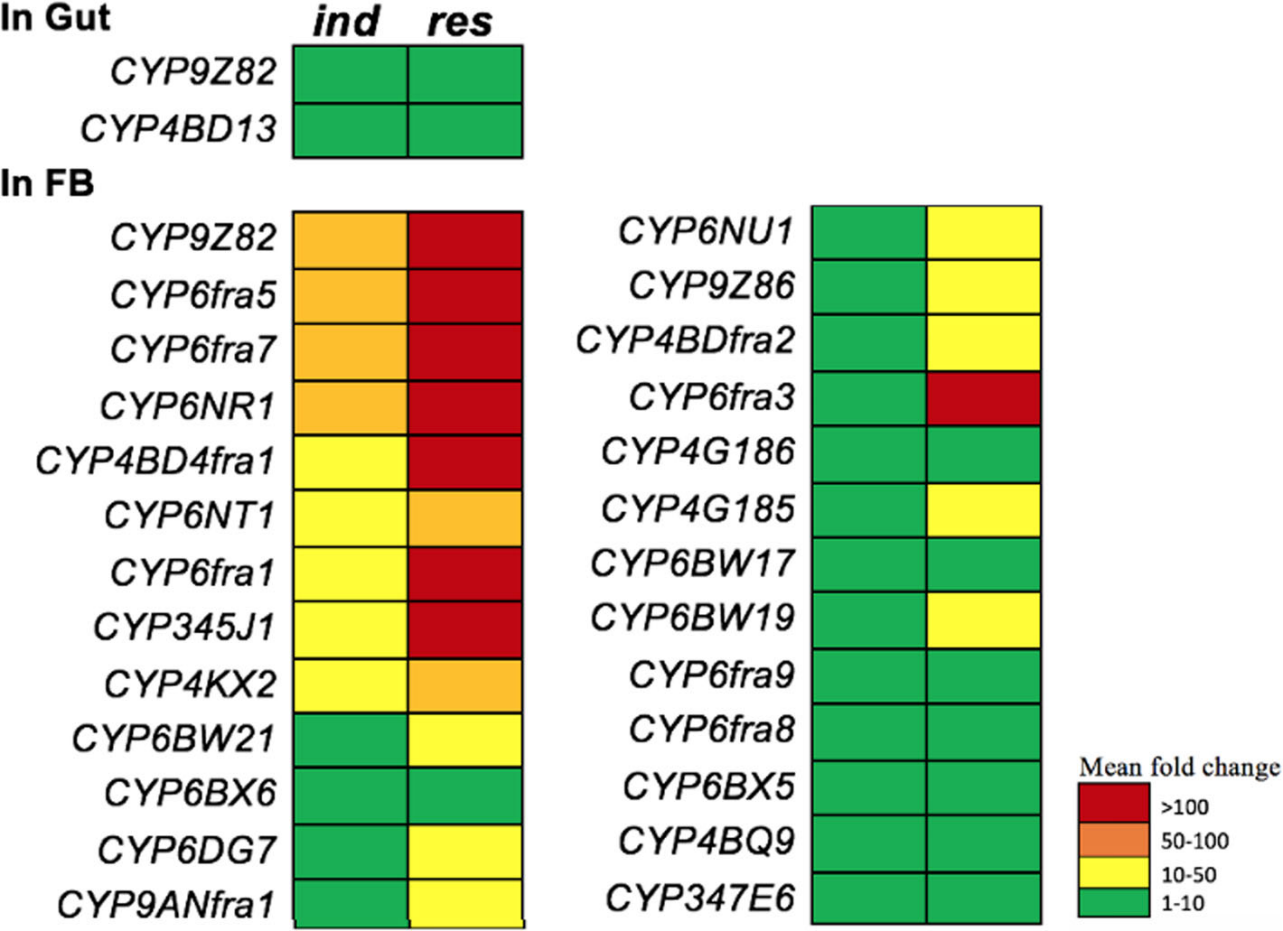
P450s differentially expressed in the gut and fat body tissues of *ind* (treated) and *res* (field-caught) beetles compared to *sus* insects are provided with colour codes indicating the mean fold change in expression

In gut tissues, only two CYP transcripts (*CYP9Z82* and *CYP4BD13*) were found to be induced with a more than five-fold increase in expression compared to *sus* strains (Fig. 5). Apart from these CYP9 and CYP4 family candidates, *CYP6NT1* is another candidate from the CYP6 family that is induced (Additional file 1: Figure S1). Interestingly, we observed that the same CYPs were overexpressed in *res* insects, which highlights the importance of these P450s in the detoxification function in the gut (Fig. 5). Apart from these three genes, four additional P450s formed the CYP4 clan; specifically, *CYP4BQ9, CYP4BG7, CYP4BD4fra1* and *CYP433A3* were overexpressed in *res* insects. The remaining P450s showed similar expression to that in the *sus* insects, exhibiting a mean fold-change in expression of less than one compared to *sus* (Fig. 5).

#### Induction in fat body tissues

Similarly, induction in fat body tissues was also calculated as the mean fold-change increase with respect to the *sus* group. In *ind* insects, four P450s (*CYP9Z82, CYP6fra5, CYP6fra7* and *CYP6NR1*) were highly induced by more than 50-fold compared to the *sus* group. Five P450s (*CYP4BD4, CYP6NT1, CYP6fra1* and *CYP345J1*) showed a more than 10-fold increase in expression compared to the *sus* group (Fig. 5). Apart from these 9 candidate genes, the expression of the remaining P450s was similar to the *sus* group, presenting mean fold-changes ranging from 0.01 to 5. In the *res* group, eight P450s were found to be overexpressed with a more than 100-fold increase in expression compared to the *sus* group, which were *CYP9Z82, CYP4BD4, CYP6fra5, CYP6fra7, CYP345J1, CYP6fra1, CYP6fra3* and *CYP6NR1*. Ten additional P450s showed a more than 10-fold increase in expression compared to the *sus* strains (Fig. 5), and the remaining P450s exhibited a mean fold-change of less than five compared to the *sus* strains. Interestingly, we observed common P450s between the *ind* and *res* groups sharing transcripts from all three CYP9, CYP4 and CYP6 families and a significantly high number (18) of P450s induced in fat body tissues compared to only two in the gut. The variation in expression between the *ind* and *res* gut tissues was significantly different (*p* = 0.015). Comparison between the gut tissues of different groups revealed a significant difference between the *res* and *sus* RPW gut samples, with a *p* value of 0.017.

### Differential induction of P450s

**Fig. 6:**
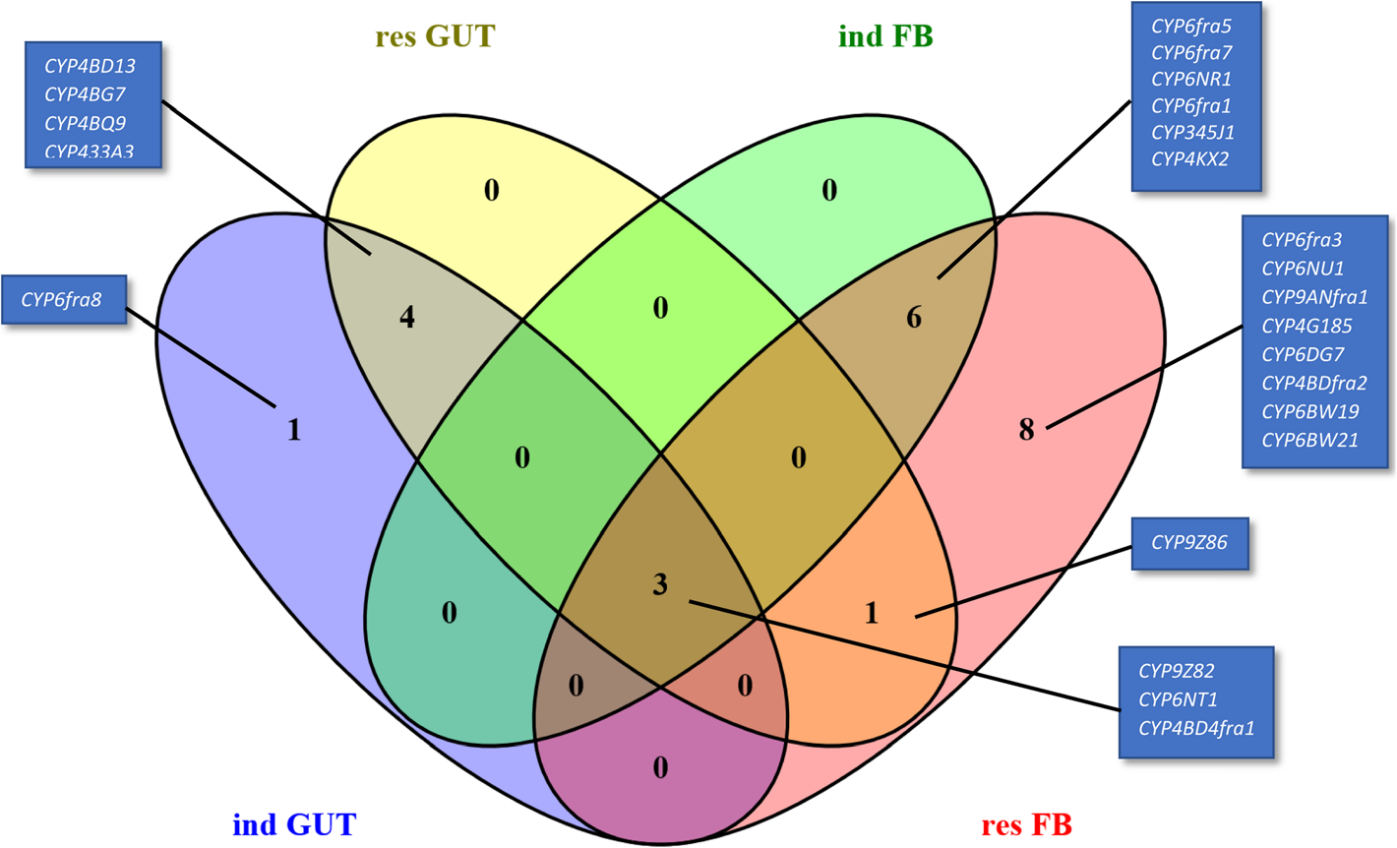
Venn diagram representing the overlap of P450 overexpression in induced (*ind*) and field-caught (*res*) RPW tissue samples: *ind* GUT, res GUT, *ind* fat body (*ind* FB) and *res* FB

We found all three major CYP families (CYP6, CYP4 and CYP9) to be differentially induced in gut and fat body tissues upon treatment with imidacloprid. Induction in the gut was no higher than10-fold, whereas in fat body tissues, 100-fold induction of some P450 genes was observed compared to the *sus* insects. Two P450s (*CYP9Z82* and *CYP4BD13*) were induced in gut tissues, whereas up to 25 P450s were induced in fat body tissues (Fig. 5), including *CYP9Z82, CYP6fra5, CYP6fra7*, and *CYP6NR1*, as highly induced genes. Apart from the ubiquitous overexpression of *CYP9Z82*, most of the P450s induced in fat body tissues were CYP4 and CYP6 candidates (Figs. 5 and 6). Additionally, three P450 genes (*CYP9Z82, CYP6NT1* and *CYP4BD4fra1*) were common to both tissues of *ind* and *res R. ferrugineus*.

In the *ind* gut, five CYP4 transcripts, two CYP6 transcripts and one CYP9 were overexpressed. In the *res* fat body, 4 CYP6 transcripts, two CYP4s and one CYP9 were overexpressed. We also identified *CYP6fra8* as being exclusively induced in the gut tissue (Fig. 6).

P450s induced in different tissue samples were detected using RT-qPCR, and the Venn diagram presented in Fig. 6 depicts overlapping P450s in the four tissue types. *CYP9Z82, CYP6NT1 and CYP4BD4fra1* were the three P450s overlapping in all four samples examined. Among the P450s overexpressed in gut tissues, four P450s were common between the *ind* and *res* samples, including *CYP4BD13, CYP4BG7, CYP4BQ9* and *CYP433A3.* Between the *res* and *ind* fat body tissues, six P450s were common, which comprised *CYP6fra5, CYP6fra7, CYP6NR1, CYP6fra1, CYP345J1* and *CYP4KX2*. We identified three P450s that were overexpressed in all tissues compared, which were *CYP9Z82, CYP6NT1* and *CYP4BD4fra1* (Fig. 6).

### Expression profiling reveals P450 clusters

**Fig. 7:**
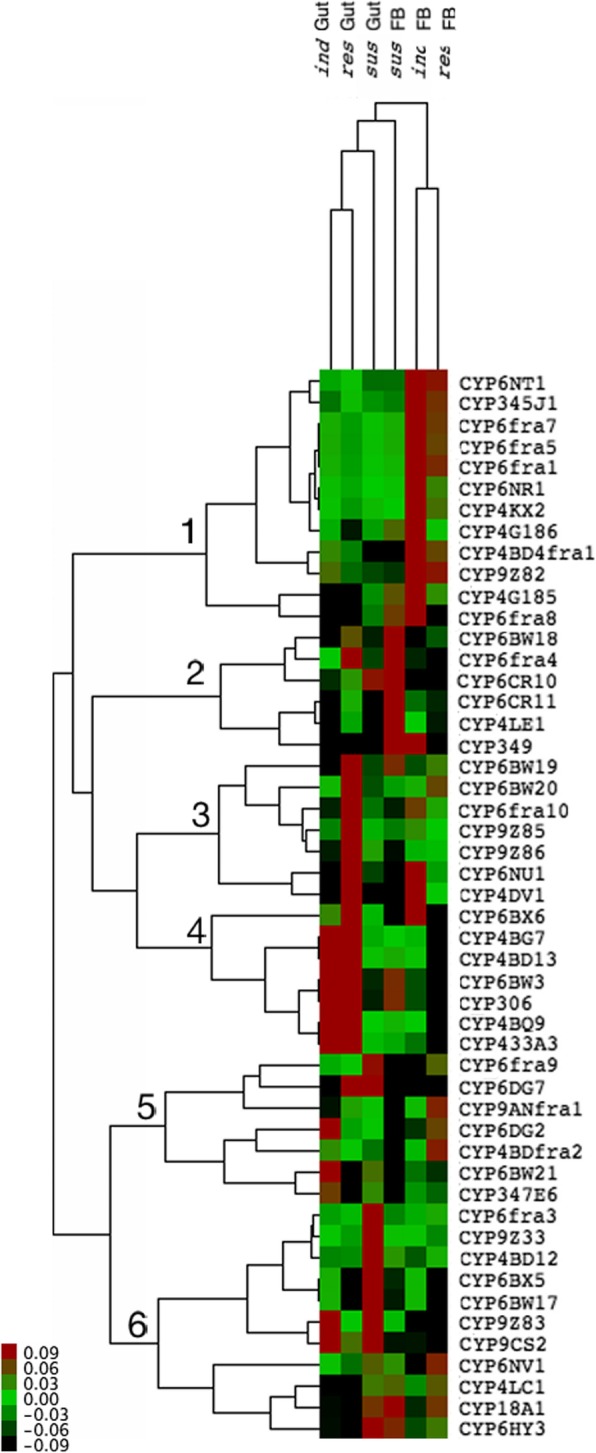
Expression profile of P450s in the gut and fat body tissues of *sus*, *ind* and *res* beetles. The relative expression of P450s in each tissue compared to *tubulin* and *β-actin* gene expression (2^-ΔCт^) is represented; red indicates overexpression, while green represents low expression, and black represents moderate expression. The six major clusters identified are indicated in the tree

Cluster analysis was performed on the RT-qPCR data (2^- ΔCt^) from gut and fat body tissues of the *sus, ind* and *res* groups revealed possible synergy between P450 transcripts. The result (Fig. 7) shows six clusters with characteristic expression patterns. Cluster one comprised 12 P450s, mainly induced in fat body tissues and includes a subgroup of seven P450s with overexpression in both *ind* and res *fat* body tissues (*CYP6NT1*, *CYP345J1, CYP6fra7, CYP6fra5* and *CYP6fra1, CYP4BD4f1, CYP9CS2*). Cluster 2 includes five P450s induced in the *sus* fat body (Fig. 7) and cluster 3 includes 7 candidates overexpressed in the *res* gut. Cluster 4 shows six interesting candidates with overexpression in both *ind* and *res* gut tissues, which were *CYP4BG7, CYP4BD13, CYP6BW3, CYP306, CYP4BQ9* and *CYP433A3*. Cluster 5 includes seven candidates induced exclusively in the *sus* fat body tissues, and 6th cluster includes 11 P450s induced mainly in *sus* gut tissues (Fig. 7). A cluster analysis conducted separately on gut and fat body tissues is provided as supporting information (Additional file 2: Figure S2). Additional file 2: Figure S2A shows three clusters identified in gut tissues with overexpression in the *res* gut and *ind* gut, while the remaining candidates presented low expression. In fat body tissues Additional file 2: Figure S2B, two clusters were identified as overexpressed cluster 1 in *res* insects and the remaining less induced P450s in another cluster (Additional file 2: Figure S2B).

### RNAi-based gene silencing of P450s

**Fig. 8:**
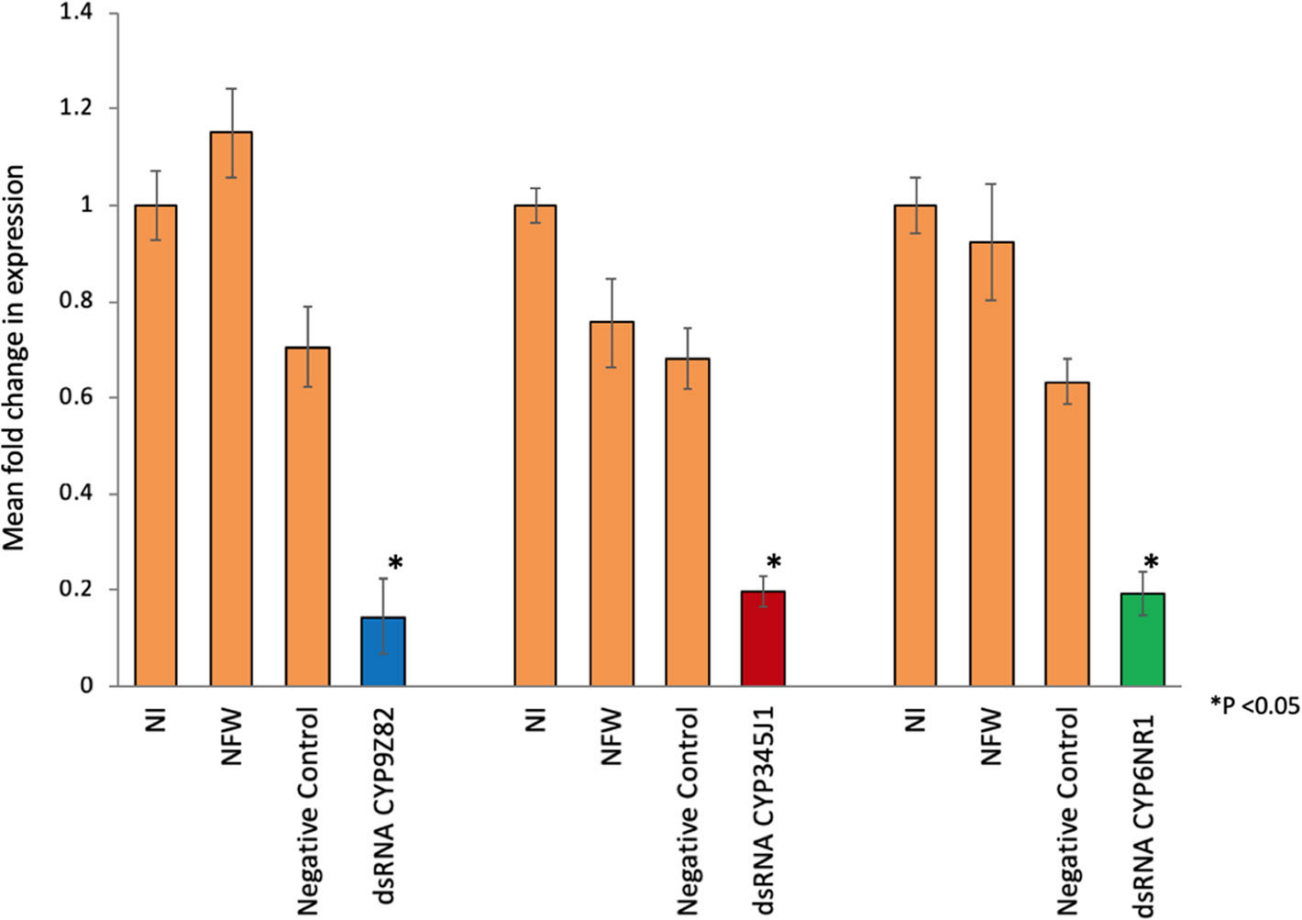
RNAi-based silencing of the selected P450s *CYP9Z82, CYP345J1* and *CYP6NR1.* Mean fold change in P450s expression in fat body tissues estimated by RT-qPCR, from different samples (NI: non-injected, NFW: Nuclease free water- injected, negative control dsRNA-injected and respective P450 dsRNA-injected). (*) represents the statistical significance measured at *p* < 0.05 and error bars represents SEM. All P450s showed a significant reduction in expression compared to the dsRNA negative control. Representative visual band gel images are provided in the insight. (*p* < 0.05; one-way ANOVA with LSD)

We selected *CYP9Z82*, *CYP6NR1* and *CYP345J1* for the RNAi experiments because the first two P450s were highly expressed and differentially expressed, respectively, and the remaining P450 was a paralog of *Drosophila Cyp6g1* [41, 75, 76, 82, 83] and *B. tabaci CYP4C64* [19], which confer cross-resistance to imidacloprid. The RT-qPCR results revealed 79.42, 54.99 and 69.76% reduction in the 2^-ΔΔCt^ values respectively in *CYP6NR1*, *CYP345J1* and *CYP9Z82* RPW adults compared to the dsRNA negative control, and a significant difference to values for NFW and NI RPWs (Fig. 8). Results from one-way ANOVA showed that injecting dsRNA for the P450 genes resulted in a significant reduction in the mRNA levels of all genes [*CYP9Z82* (*p* = 0.008), *CYP6NR1* (*p* = 0.029) and *CYP345J1* (*p* = 0.001)] compared to the dsRNA negative control RPWs (Fig. 8).

#### Phenotypic effects and validation of gene silencing by RT-qPCR

**Fig. 9:**
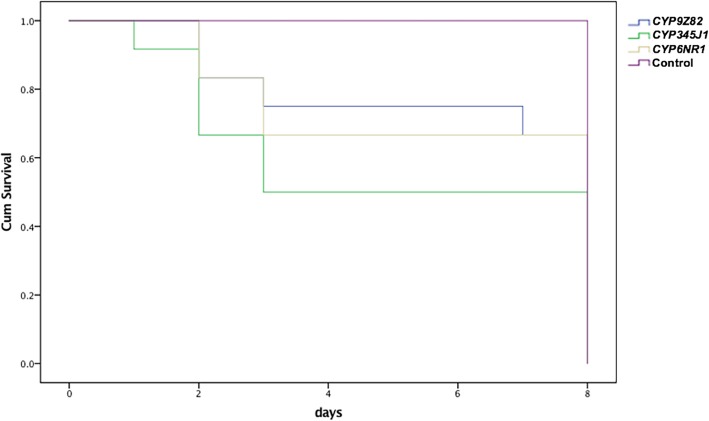
The Kaplan-Meier survival analysis provides the survival functions from three groups of P450-silenced insects, represented as *CYP9Z82* (blue), *CYP345J1* (green), *CYP6NR1* (yellow), and control (violet) line diagrams. The experiment was censored after 11 days and the data represents comparison of 8 days, till the survival rate of silenced insects reached 0. The survival functions calculated based on the Mantel-Cox Log Rank test showed no significant difference in survival functions between the silenced groups (*p* = 0.054, Mantel-Cox Log Rank test)

The percentages of mortality observed in *CYP9Z82*-*, CYP6NR1*- and *CYP345J1*-silenced insects were determined to be 25, 33 and 50%, respectively, upon exposure to a sublethal concentration of imidacloprid for 11 days. The Kaplan-Meier survival analysis between the silenced insects showed no significant difference in survival functions between the silenced groups (*p* = 0.054, Mantel-Cox Log Rank test) (Fig. 9). However, the 50% mortality observed in *CYP345J1*-silenced insects upon imidacloprid treatment demonstrates the importance of this gene in imidacloprid detoxification.

**Fig. 10:**
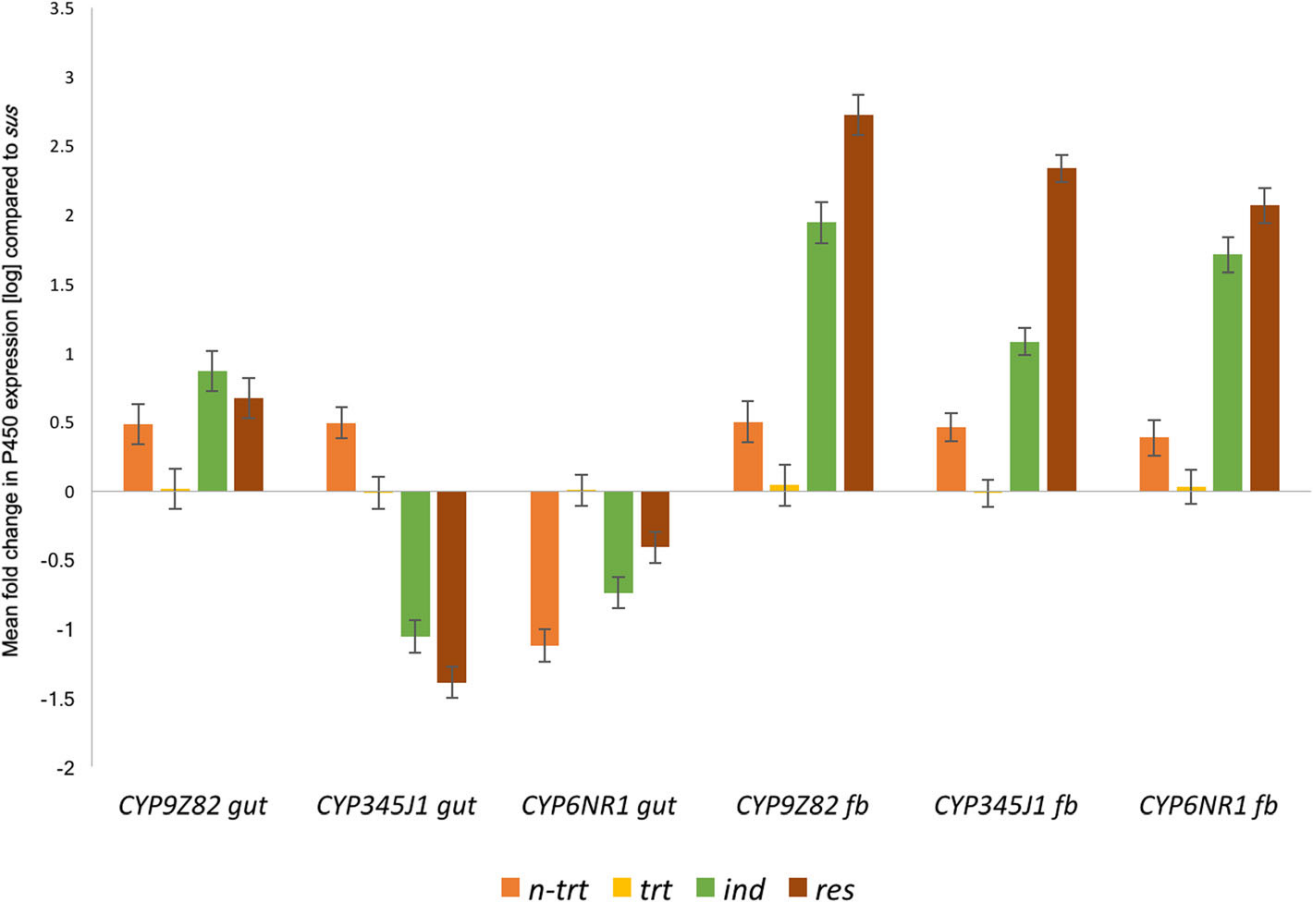
The figure represents relative expression of the *CYP9Z82*, *CYP345J1* and *CYP6NR1* genes in the gut and fat body tissues of different experiment groups, silenced and imidacloprid treated (*trt*), silenced and non-treated (*n-trt*), non-silenced and imidacloprid treated (*ind*), and field (*res*), compared to non-silenced and non-treated (*sus*) group. The bars represent mean fold change in expression compared to *sus* group. Each sample group was color coded as *n-trt* (orange), *trt* (yellow), *ind* (green) and *res* (brown). Error bars represent SEM. The statistical significance was measured using Tukey’s HSD with *p* < 0.05 as the level of significance

The expression status of P450s was further analyzed in the gut and fat body tissues of the silenced insects, which allowed us to compare the effect of imidacloprid treatment in injected, treated and surviving insects. There were significant differences (*p* = 0.010) in each group compared to the *sus* group, especially in fat body tissues. The comparison showed that the *ind* group presented the greatest differences between the genes (*p ≤* 0.001)*,* followed by the *n-trt* (*p* = 0.042), *trt* (*P* = 0.059), *sus* (*p* = 0.011) and *res* (*p* = 0.015) groups. For *CYP9Z82,* which displayed the highest expression in the *ind* and *res* strains (> 100-fold), showed no significant increase in expression after treatment in either gut or fat body tissues. *CYP345J1,* with > 10-fold and > 50-fold expression in the *ind* and *res* strains, respectively, also showed low expression in *trt* samples. Likewise, *CYP6NR1* showed a similar expression pattern with high expression in the gut *ind* and *res* strains (> 100-fold and > 10-fold, respectively) and low expression in *trt* samples. These results show that the silencing of these genes was lower in all treated samples, and thus, mortality rates can be considered to be a factor that represents the importance of all three P450s in contributing to imidacloprid resistance (Fig. 10).

## Discussion

The RPW, *R. ferrugineus,* is the most invasive and globally important quarantine pest [42–45]. The cause of the high rate of spread of this pest is human intervention via transport of infested young or adult date palm trees and offshoots from contaminated areas into uninfected areas [43]. As the earliest commercial neonicotinoid, imidacloprid has been extensively used for the control of *R. ferrugineus* in date palm cropping systems in Middle Eastern countries [42]. However, the widespread application of imidacloprid has resulted in the development of resistance, which has emerged as a major problem for *R. ferrugineus* control [50]. Differential expansion and diversification of P450 subfamilies were observed in *R. ferrugineus*, and these changes are possible to be linked to adaptations to novel ecological niches. We showed that dsRNA injection and silencing of the *CYP345J1* and *CYP6NR1* genes resulted in a significant decrease in the survival rate of imidacloprid-treated adult weevils. Hence, based on our RNAi results, we conclude that constitutive overexpression of several P450s and selective detoxification involving at least *CYP345J1* and *CYP6NR1*, along with several other enzyme systems, are involved in imidacloprid detoxification in RPWs in a date palm field.

The current study provides the global transcriptome repertoire of RPW P450s and a comprehensive picture of the *R. ferrugineus* CYPome, based on the identification of genes and imidacloprid-induced expression profiling. Our tissue-specific expression analysis revealed nearly ubiquitous expression patterns, including some P450s with possible duplications or pseudogenes that are not expressed. Our identification of 77 P450s from *R. ferrugineus,* which belong to 22 families and 45 subfamilies; is more or less similar to previous reports on other insects, such as *Bombyx mori,* exhibiting 84 P450s, *L. decemlineata*, exhibiting 96 P450s [23], and *D. melanogaster,* exhibiting 85 P450s [77]. However, the total P450s in *R. ferrugineus* is relatively low compared to the numbers identified in other coleopteran model insects including *T. castaneum* (143 P450s) [79], as genome sequence data become available for *Tribolium*.

The phylogenetic analysis of *R. ferrugineus* P450s with other insect species revealed that insect P450s have diverged over time into different groups leads to species-specific and lineage specific expansions (Figs. 1, 2 and 3), consistent with the earlier studies [6, 14, 84]. *R. ferrugineus* P450s CYP2 and mitochondrial clans showed a high degree of sequence homology with other insects, indicating functional conservation of these P450s. Notably, *CYP18A1, CYP305A1* and *CYP306A1* from the CYP2 clan, and *CYP12, CYP302A1, CYP314A1* and *CYP315A1* from the mitochondrial clade were clustered into one group with > 90% bootstrap support, indicates that these P450s may be originated from a common ancestor and differentiated during evolution linked to adaptations. Several earlier studies have demonstrated that P450s in the CYP2 and mitochondrial clan were generally associated with the ecdysteroid biosynthesis such as 306A1 encoded by *Phantom* gene in *D. melanogaster* and *B. mori* [85, 86]*, T. cataneum* [79]*, L. decemlineata* [87] and *A. mellifera*; 18A1 encoded an ecdysteroid 26-hydroxylase/oxidase activities in *D. melanogaster* [88]*; CYP314A1* and *CYP315A1* encoded by Halloween genes *Shadow and Shade* respectively in *D. melanogaster* [81], and *CYP301A1* involved in the ecdysone regulation in *D. melanogaster* [89]. Whereas, *CYP49A1* and *CYP18A1* are expressed in the integument during *Drosophila* development [90, 91], and CYP12 family genes associated xenobiotic metabolism in *D. melanogaster* and *A. gambiae* [84]. In the *R. ferrugineus* CYP2 and mitochondrial clans, we can speculate based on above mentioned studies that *CYP305A1, CYP306A1, CYP301A1, CYP314A1*, and *CYP315A1* may be involved in ecdysone biosynthesis; CYP12 family genes associated xenobiotic metabolism, and *CYP49A1* and *CYP18A1* in the integument during *R. ferrugineus* development.

Members of the CYP3 clan are reported to be involved in xenobiotic and insecticide metabolism, and are induced by the exposure of pesticides and natural products [7, 9, 37, 41]. The phylogenetic analysis showed that genes in Clan 3 and 4 appear to undergo multiple expansion and extensive species-specific clustering leads to CYP “blooms”. Most P450s in the CYP3 clan had gene orthologos in *T. castaneum* [79] and *L. decemlineata* [23, 87], or gene expansion with close phylogenetic relationships (Fig. 2). P450s in the CYP6 family had several functions. For instance, *CYP6G1, CYP6BQ, CYP6CM/CYP6ER1/CYP6AY1* and *CYP6D/CYPBG* subfamilies are reported to be involved in DDT, deltamethrin, imidacloprid and pyrethroid metabolism respectively in several insects [19, 27, 75, 92]. *R. ferrugineus* CYP6 phylogenetic analysis clustered with several orthologs CYP6 subfamily genes reported earlier, notably, *CYP6G1, CYP6EZ1, CYP6EH1, CYP6AY1* and *CYP6CM1*, which are associated with imidacloprid resistance (Fig. 2). Our phylogenetic analysis suggesting the potential functions of these *R. ferrugineus* P450s in detoxification of imidacloprid in the date palm field. However, many other P450s in the CYP6 family genes have been reported to be involved in detoxification of plant allelochemicals and had various functions. For instance, *CYP6B* subfamily genes reported to be involved in the metabolism of plant toxins furanocoumarins to nontoxic compound in the insect order Papilionidae [93], and *CYP6A* subfamily genes associated with pheromone-sensing, and its expression level in the olfactory sensilla associated with the social behavior, aggressiveness in *D. melalanogastor* [94, 95]. The second largest family in the Clan 3 is CYP9, and several P450s in the family is reported to be associated with insecticide and odorant metabolism in insects [96, 97]. In our study, three CYP9 subfamily P450s (*CYP9Z, CYP9AN* and *CYP9CS*) displayed close phylogenetic relationship with *T. castaneum* and *L. decemlineata*, however, high degree of species-specific expansion reflecting adaptation to environmental xenobiotics. In the Clan 4, CYP4 is the largest gene family identified so far; and our phylogenetic analysis identified several potential P450s orthologs and paralogs associated with biosynthesis of endogeneous compounds [98, 99], pyrethroid resistance [18, 100] and pheromone metabolism [101]. Gene expansion and species specific CYP “bloom” is common in the CYP4 family as reported in other insects [14, 84], particularly in the *CYP4BD* and *CYP4G* cluster. The orthologs CYP4, *CYPQc* and *CYP4Qb* from *L. decemlineata* reported to be involved in the imidacloprid resistance [23] were found grouped with *R. ferrugineus* CYP4Q6 and *CYP4KX2*, indicating a common ancestor (Fig. 1). *CYP4G7* and *CYP4G61* are thought to be a strong candidate for enhancing the cypermethrin and pyriproxyfen resistance respectively in *T. castaneum* [27] and *Trialeurodes vaporariorum* [102]. Similarly, the *CYP4G25* is associated with diapause in *Antheraea yamanai* [103]. Such diversity in CYP4G and CYP4D subfamily genes indicating a diverse function.

P450s are well known for their role in detoxification mechanisms in a wide range of organisms, including humans. In insects, the major sites of detoxification are the head, gut and fat body, among which the fat body is the most important [22, 23]. We identified three CYP6 candidates and one CYP9 candidates that were highly overexpressed in the fat body of *ind* RPWs in a laboratory bioassay (Fig. 5). Interestingly, we observed that imidacloprid-induced transcripts in *ind* insects were also highly overexpressed in field-caught *res* samples, indicating that they may play a role in the development of resistance in response to the heavy doses of imidacloprid used in the date palm field in Saudi Arabia. Among CYP families, 6, 4 and the *CYP345* family have been reported to be involved in imidacloprid resistance in various insects (Additional file 4: Table S2) [19, 23, 27, 80, 104–108], and the current study specifically focused on genes induced in response to imidacloprid treatment.

Interestingly, the candidate (*CYP345J1*) with the highest RPKM value shows high sequence similarity to *CYP6g1,* a well-studied DDT and imidacloprid cross-resistance gene from *D. melanogaster* [75, 80, 109] and *D. simulans* [82]; this candidate is overexpressed in the fat body of *ind* and *res* RPWs (Fig. 5). *CYP345A1* from *T. castaneum* is reported to be paralog of *CYP6g1* [27] and shares 52% nucleotide identity with *CYP345J1,* which was significantly upregulated by imidacloprid. Another candidate, *CYP4LE1,* is a paralog (53% nucleotide identity) of *B. tabaci CYP4C64*, which is associated with imidacloprid resistance [19, 20]. *R. ferrugineus CYP4BD4* is overexpressed and induced in both *res* and *ind* RPW populations (Fig. 7). Notably, *CYP4BD4* expression was higher in the RPW fat body than in the gut, indicating that metabolic detoxification and catalytic activity are more prevalent in the fat body. More interestingly, our bioassay revealed imidacloprid-induced transcripts, with 9 more transcripts being overexpressed at a higher level in the *ind* fat body than in the gut. Our induction data from the *ind* fat body also highlight the role of multiple P450s involved in the metabolic detoxification process (Fig. 5), which supports the recent identification of the induction of multiple P450s [75] in *L. decemlineata* [23]. The promising imidacloprid-induced P450 transcripts that may lead to resistance included six common transcripts found in both *ind* and *res* fat body tissues as well as four common transcripts found in the *ind* and *res* gut. Interestingly, 4 CYP6 family transcripts fell into this category for the fat body, whereas the CYP4, CYP6 and CYP9 families were all considered to be involved in detoxification in the gut (Fig. 6).

Intriguingly, the overlap of P450s induced in different tissues indicates that these enzymes act at different detoxification sites, especially in the gut and fat body. The largest number of P450s was induced (37 of the 50 studied) in the *res* fat body (Additional file 1: Figure S1). Half of all P450s studied were also found to be induced in the *res* gut, indicating the possibility of high rates of detoxification in this organ. We identified imidacloprid-induced genes that overlap with overexpressed/resistance genes from *res* insects [gut 9:25 (1:1.3), fat body 22:37 (1:1.7)]. Moreover, the highly induced candidate gene *CYP9Z82*, showed higher induction in the fat body compared to the gut. This transcript, with a > 100-fold increase in expression, also exhibits 65% identity with *CYP9Zg* (GenBank: XP_023014564) from *L. decemlineata*, a gene reported to be involved in imidacloprid resistance [23]. Similarly, we found three RPW CYP4 candidates (*CYP4G185, CYP4G186 and CYP4G187*) showing 51–59% nucleotide identity with *T. castaneum CYP4G7*, a gene reported to be involved in imidacloprid resistance [27]. Among these candidates, only *CYP4G186* was overexpressed in the fat body of *ind* and *res* RPWs. These results indicate that CYP family members are differentially induced between various strains of beetles and that even closely related insects exhibit limited P450 functional similarity. As only an induction profile for *L. decemlineata* CYP is available for comparison [23], there is a significant need for more studies on insect CYPomes and induction profiles.

Considering the large number of P450s reported in insects and their possible roles in different biosynthetic and detoxification mechanisms, one approach to identifying P450s involved in imidacloprid detoxification is to develop an induction profile in response to imidacloprid. In the whitefly *B. tabaci,* increased expression levels of *CYP6CM1 and CYP4C64* are reported to be associated with imidacloprid resistance [19, 20], and in *N. lugens,* overexpression of *CYP6ER1* and *CYP6AY1* are closely linked to imidacloprid resistance [21, 22]. Furthermore, in the Colorado potato beetle *L. decemlineata,* upregulation through the induction and constitutive overexpression of multiple genes of the CYP4, 6 and 9 families was found to be associated with imidacloprid resistance [23]. We identified six clusters or expression patterns for P450s that can provide insight into the P450s involved in imidacloprid resistance. The four transcripts from cluster 2 have key detoxification functions in fat body tissues, as supported by 13 transcripts from cluster 1. Fifteen candidates identified in clusters 4 and 5 are also proposed to be involved in the detoxification of insecticides and plant toxins in gut tissues (Additional file 1: Figure S1).

We further evaluated the role of three overexpressed P450s in imidacloprid detoxification by silencing those genes using the RNAi method, followed by imidacloprid bioassays. Our results showed that 79.42 and 54.99% silencing of *CYP6NR1* and *CYP345J1*, respectively, resulted in 33 and 50% mortality in RPW adults in toxicity bioassays. Similar results showing that up to 76% silencing of *CYP9e2-like* resulted in 35.78% mortality in *B. odoriphaga* have been reported [29], and silencing of *CYP4Q3* resulted in 46.7% mortality in *L. decemlineata* in an imidacloprid toxicity bioassay [24]. The 50% mortality observed in *CYP345J1*-silenced insects was also comparable to the 55.1% mortality achieved for *CYP6AY1*-silenced *N. lugens* [110]. The sole CYP9 RNAi candidate from RPW, *CYP9Z82*, was associated with only 25% mortality upon imidacloprid treatment, which was similar to the 25.3% mortality achieved in *CYP9AQ2*-silenced *L. migratoria*, although this was determined in a deltamethrin bioassay [111]. Considering the mortality rates achieved, it is reasonable to conclude that the three P450s that we studied play key roles in imidacloprid detoxification in RPW. The 33 to 50% mortality achieved in the current experiment is promising considering the fact that other enzymes, such as UDP-glucosyl transferases (UGTs), GSTs, Ests and ATP-binding cassette transporters, are possibly important in imidacloprid detoxification, as reported recently in *L. decemlineata* [24]. Considering that nearly equal silencing levels were achieved for all three genes, it is possible to differentiate the importance of P450s based on the mortality rates achieved. Thus, this study confirms the importance of two CYPs (*CYP6NR1* and *CYP345J1*) in imidacloprid detoxification. Three factors that we need to consider further before targeting these genes as a P450-based pest control strategy are the species-specificity of these genes, the role of other detoxification enzymes and the possible synergism between them. Considering the development of resistance as a multistage process with multiple contributing factors, it is also important to study the role of mutations as well as the role of other detoxification enzymes, especially role of GST, as recently reported in house flies [112]. It will be more interesting to study role of regulatory genes, such as those related to xenobiotic factors, in the overexpression P450 genes [113].

## Conclusion

Our study presents 77 cytochrome P450s in *R. ferrugineus*, which belong to 22 families and 45 subfamilies, and the results provide a basis for the further molecular and functional characterization of P450s. The current study demonstrates that multiple P450s are overexpressed and differentially expressed in field-caught RPW populations and in laboratory-induced (*ind*) strains, which should be considered an indicator of metabolic tolerance to imidacloprid. The important findings of our work included the functional identification of *CYP345J1* and *CYP6NR1*; constitutive overexpression of these genes presumably allows insecticide molecules to be metabolized more efficiently, resulting in enhanced tolerance of RPWs in a date palm field. In the present study, we used three highly expressed P450s in the functional analysis; together with other differentially expressed P450s as well as Ests, GSTs and ATP-binding cassette transporters, our data help to explain imidacloprid resistance in RPW, though some components of this mechanism remain to be identified. Insecticide resistance in RPW is a major problem faced in date palm cultivation and is a major reason for the spread of *R. ferrugineus* around the world. Biochemical assays for detecting metabolic resistance due to P450s are increasingly being used in mosquitoes [40, 114]. Similarly, with the identification of P450s associated with imidacloprid resistance, the development of more specific diagnostic molecular markers is highly recommended for the screening of P450 overexpression in *R. ferrugineus* that confers metabolic resistance to imidacloprid. RNAi has the potential to be used to target upregulated molecular mechanisms of resistance and could be a practicable option for RPW management and needs to be studied in depth.

## Additional files

Additional file 1: **Figure S1.** P450s induced in the gut and fat body tissues of *ind* and *res* strains represented as the mean fold-change in expression compared to the respective *sus* gut/fat body tissues (2^-ΔΔCт^). P450s are arranged in descending order of the mean fold-change in expression from higher (green) to lower (yellow). (TIF 1586 kb)

Additional file 2: **Figure S2.** Cluster analysis of P450s in gut (A) and fat body (B) tissues of *ind*, *sus* and *res* RPW strains. The relative expression of P450s in each tissue compared to tubulin and β-actin expression (2^-ΔCт^) was used in cluster analysis, and expression levels are indicated with red (overexpression), green (low expression), and black (moderate expression). The major clusters identified are marked in the tree. (TIF 910 kb)

Additional file 3: **Table S1.** Lists of the oligonucleotide primers with amplicon size, Tm, min-max Ct and primer efficiency. (XLSX 20 kb)

Additional file 4: **Table S2.** *Rhynchophorus ferrugineus* P450 functional annotation and classification. (PDF 12 kb)

Additional file 5: **Table S3.** *Rhynchophorus ferrugineus* P450s nomenclature and blastx homology. (XLSX 15 kb)

Additional file 6: **Table S4.** Toxicity assay of Confidor® 350 SC against susceptible and field-caught adult RPWs over 4 days of exposure. (PDF 366 kb)

Additional file 7: **Dataset S1.** P450s identified in this study, FASTA format file. (PDF 497 kb)

## Data Availability

All relevant data are within the paper and its supporting additional files. The RPW P450 FASTA nucleotide sequence information is given in Additional file 7: Dataset S1. The P450 nucleotide sequence can be obtained from the Transcriptome Shotgun Assembly project DDBJ/EMBL/GenBank under accession number GDKA00000000. The RPW P450 contig names are provided in Additional file 5: Table S3 and can be assessed at the GenBank (for example, the GenBank acc. no. of *Rfer_c29199_CYP6BW4* is GDKA01029199.

## Abbreviations

*dsRNA control*: Universal negative dsRNA control
FB: Fat body
*ind*: Susceptible strain of RPWs treated with imidacloprid
LC_50_: Lethal concentration 50
*NFW*: Nuclease free water-injected RPWs
*NI*: No-injection RPWs
*n-trt*: Susceptible strain of RPWs injected with P450 dsRNA and treated with water
P450: Cytochrome P450-dependent monooxygenases
*res*: Field-caught strain of RPWs treated with imidacloprid
Rfer: *Rhynchophorus ferrugineus*
RPW: Red palm weevil
*sus*: Susceptible strain RPWs treated with water
*trt*: Susceptible strain of RPWs injected with dsRNA and treated with imidacloprid
