# Supplementary figures and images for: Global transcriptome profiling and functional analysis reveal that tissue-specific constitutive overexpression of cytochrome P450s confers tolerance to imidacloprid in palm weevils in date palm fields
Source: BMC Genomics. 2019 May 31;20:440. doi: 10.1186/s12864-019-5837-4 (PMC6545022; doi:10.1186/s12864-019-5837-4)

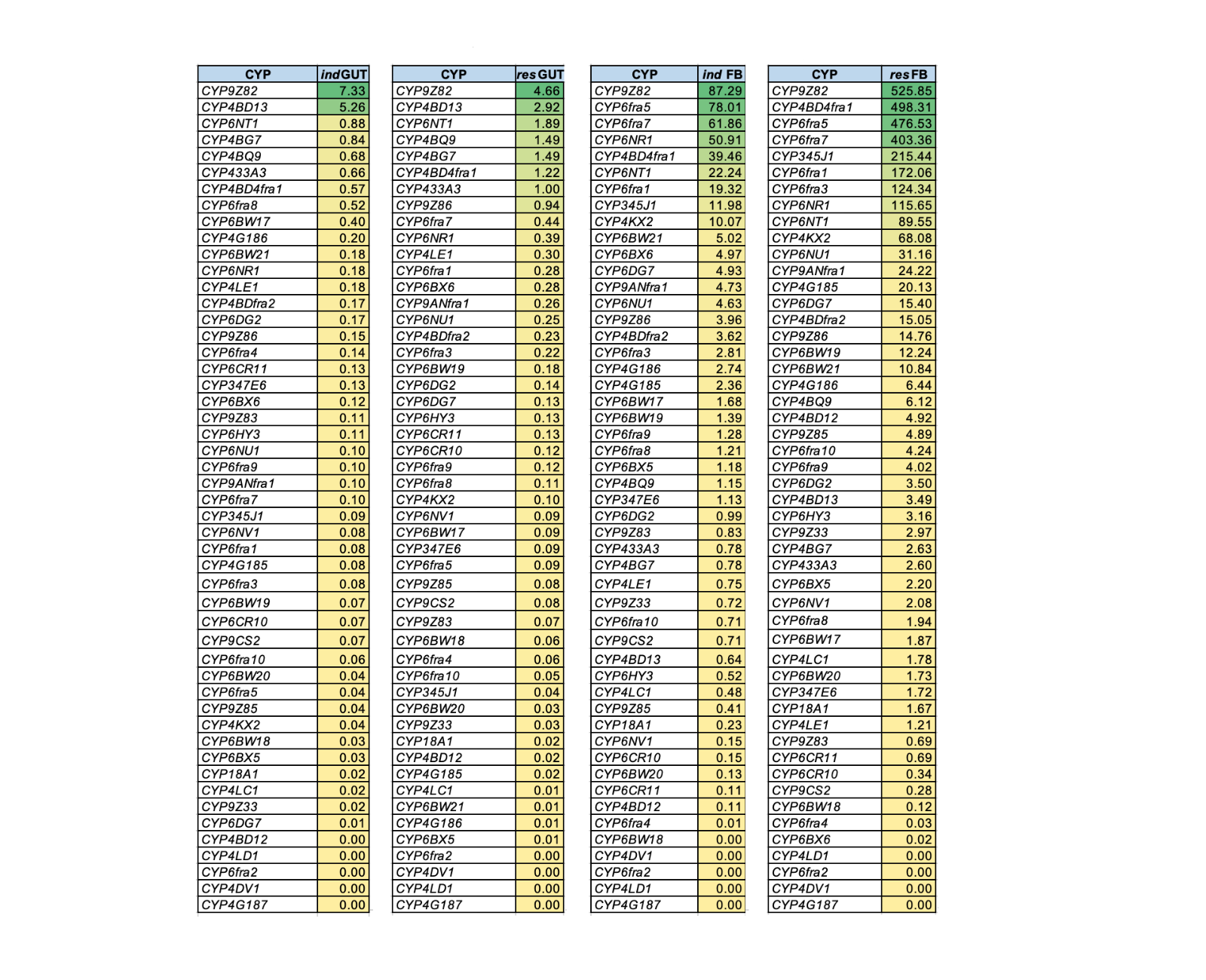

Supplement: Supplementary file 1 — Figure S1. P450s induced in the gut and fat body tissues of ind and res strains represented as the mean fold-change in expression compared to the respective sus gut/fat body tissues (2-ΔΔCт). P450s are arranged in descending order of the mean fold-change in expression from higher (green) to lower (yellow). (TIF 1586 kb) [file 12864_2019_5837_MOESM1_ESM.tif]

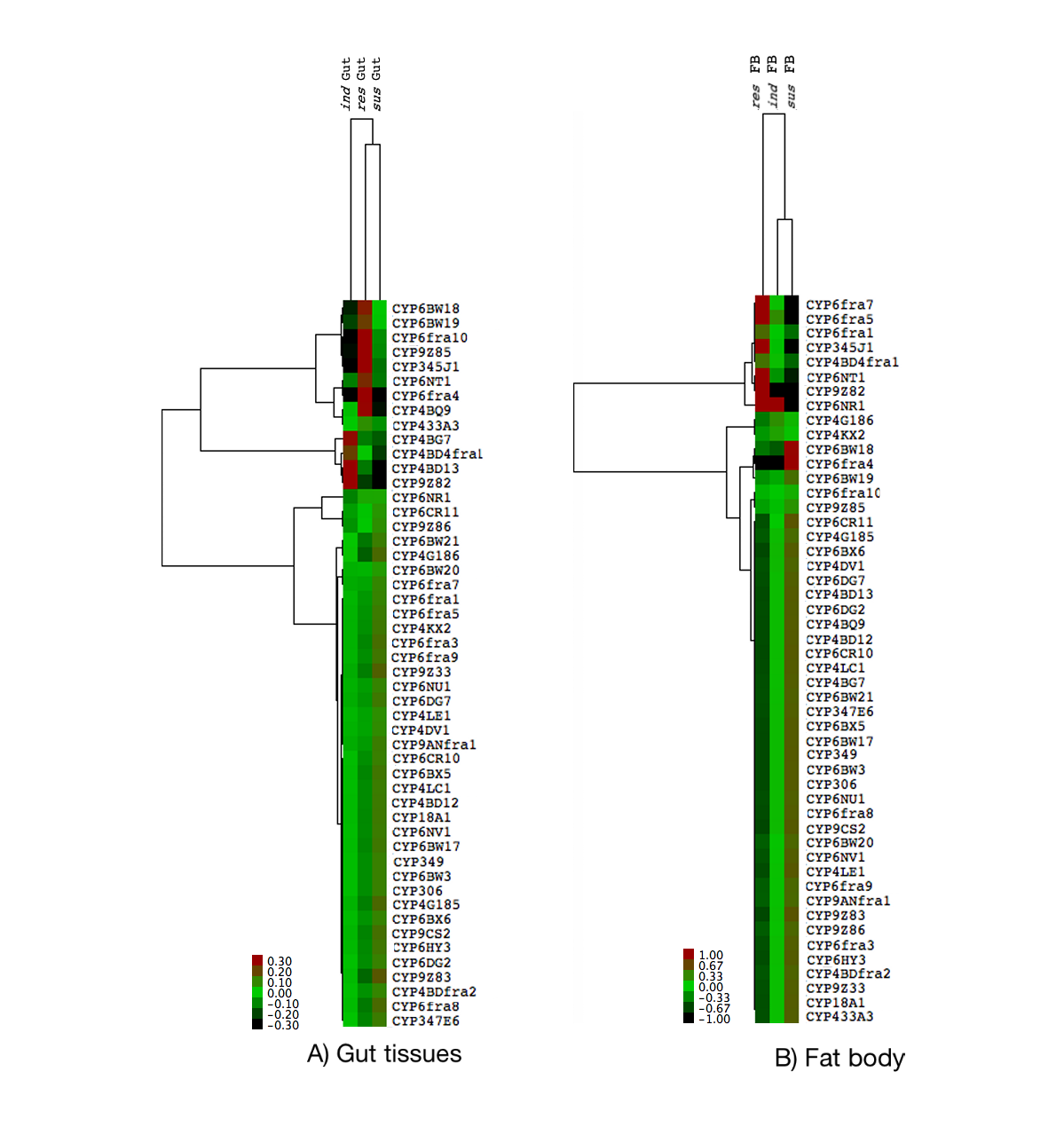

Supplement: Supplementary file 2 — Figure S2. Cluster analysis of P450s in gut (A) and fat body (B) tissues of ind, sus and res RPW strains. The relative expression of P450s in each tissue compared to tubulin and β-actin expression (2-ΔCт) was used in cluster analysis, and expression levels are indicated with red (overexpression), green (low expression), and black (moderate expression). The major clusters identified are marked in the tree. (TIF 910 kb) [file 12864_2019_5837_MOESM2_ESM.tif]
