# Supplementary material for: Global transcriptome profiling and functional analysis reveal that tissue-specific constitutive overexpression of cytochrome P450s confers tolerance to imidacloprid in palm weevils in date palm fields
Source: BMC Genomics. 2019 May 31;20:440. doi: 10.1186/s12864-019-5837-4 (PMC6545022; doi:10.1186/s12864-019-5837-4)
Supplement: Supplementary file 4 — Table S2. Rhynchophorus ferrugineus P450 functional annotation and classification. (PDF 12 kb) [file 12864_2019_5837_MOESM4_ESM.pdf]

Table S2. *Rhynchophorus ferrugineus* P450s functional annotation and classification.

	R. ferrugineus **NCBI SRA acc. SRX877682**	***R. ferrugineus*** **NCBI SRA acc. SRA045820**
Total number of reads	194,157,678	631,425
Total length of reads (bp)	19,609,925,478	324,204,057
Total number of reads cleaned	187,637,734	624,107
Total length of reads cleaned (bp)	18,764,192,058	288,373,233
Number of singletons	65,445,353	169,721
Total length of singletons (bp)	6,548,569,140	74,582,402
Average length of singletons (bp)	100.06	439.44
Largest singleton (bp)	102	845
Number of contigs	35,667	17,508
Total length of contigs (bp)	30,576,631	15,617,534
Average length of contigs (bp)	857	892
Largest contig (bp)	42,221	10,464
Average sequencing depth of contigs	342,021 X	12,105 X
N50 of contigs (bp)	1557	1014
GC content of contigs	38%	44%
