# Supplementary material for: Global transcriptome profiling and functional analysis reveal that tissue-specific constitutive overexpression of cytochrome P450s confers tolerance to imidacloprid in palm weevils in date palm fields
Source: BMC Genomics. 2019 May 31;20:440. doi: 10.1186/s12864-019-5837-4 (PMC6545022; doi:10.1186/s12864-019-5837-4)
Supplement: Supplementary file 6 — Table S4. Toxicity assay of Confidor® 350 SC against susceptible and field-caught adult RPWs over 4 days of exposure. (PDF 366 kb) [file 12864_2019_5837_MOESM6_ESM.pdf]

Table S4. Toxicity assay of Confidor® 350 SC against susceptible and field-caught adult RPWs over 4 days of exposure.

**Time (day)**	**RPW strain**	**LC_{50} (mM)**	**Lower - Upper limits**	**slop ± SE**	***r***	**χ^2 (*df*=11)**	***RR***
**day 1**	susceptible	6.30	5.24 – 7.53	5.40 ± 0.68	0.96	5.17	1.45
	field-caught	9.11	8.03 – 11.27	4.17 ± 0.66	0.99	2.37	
**day 2**	susceptible	5.66	4.49 –7.00	5.23 ± 0.66	0.94	5.92	1.40
	field-caught	7.92	7.22 –9.06	4.73 ± 0.66	0.99	0.42	
**day 3**	susceptible	4.65	4.36 –4.93	6.21 ± 0.69	0.99	1.07	1.45
	field-caught	6.75	6.22 –7.48	4.37 ± 0.51	0.99	1.48	
**day 4**	susceptible	3.44	2.76 –3.81	5.46 ± 0.44	0.96	7.94	1.73
	field-caught	5.97	5.53 –6.50	4.40 ± 0.47	0.98	2.99	

Resistance ratio (RR) = LC_{50} of field-caught RPWs/LC_{50} of susceptible RPWs
