# Supplementary material for: Global transcriptome profiling and functional analysis reveal that tissue-specific constitutive overexpression of cytochrome P450s confers tolerance to imidacloprid in palm weevils in date palm fields
Source: BMC Genomics. 2019 May 31;20:440. doi: 10.1186/s12864-019-5837-4 (PMC6545022; doi:10.1186/s12864-019-5837-4)
Supplement: Supplementary file 7 — Dataset S1. P450s identified in this study, FASTA format file. (PDF 497 kb) [file 12864_2019_5837_MOESM7_ESM.pdf]

Dataset S1. P450s identified in this study, FASTA format file (contig name_CYP name).

> RferU1_CYP6MS2

TAGACTATATAAGACCTGTATTCATAGACGGTGGGGATAGTTCTTGCGATAGTCAGGCCACGATGTTGTTGTATGT
AGTCTTGGTGTTAGTTGCTTTCTTGGTTTACGTAAAATTTAAACAATCTTATTGGAAACGCCGAGGTCTACATCAAC
TGGAACCAGAATTTTTCTTCGGTAACGCTAAGAAGGAACTGCTTGGTAAACGATCAATATTCCTTCTCTTCCGAGAT
ATTTACGAGAAAGCCAAAGTTCTGAAGCAGGATCACGTCGGAATATACTTCTTCCTGGAGCCAATTTACATCCCAA
CGAACTTGACCATCATCAAACAGATTTTGATAAAGGACTTCCAACATTTCTGGGGCCATGGCGGCTTCTCGCATCCC
AATGAGCGTCTGTCGATGAATCTGTTCAACTTGGAGGGCGATCCTTGGCGACATCTACGCACCAAACTGACCCCCA
CGTTCACGTCCGGTAAAATGAAGATGATGTTTGATACTTTGGTGTCTAAAACCGACGGTTTGGAGAAACTAATCGA
TGGTTACATCACCCGCAAAGAACCGGTTGATATAAAGGAAGCTGCAGCTCGGTTTACCACGGATATCATAGCCAGT
TGCGCGTTCGGTATTGAGACTGATGCCATGGAGAACGACCGGAATGATTTCCGAACATATGGAAGAAAAGTCTTC
AAACCAACAACGTTTAAATTGTTTCTTAGTAACACTGTACCGGCATCAGTTTTAGGTCCATTGGGTTTCAAACTATTT
CCTCGAGATGTAGACGCATTCTTTTCCGAAACTGTGTTGGATACCATGCAATACCGAGAGAAATATAACATAATAC
GTAAAGATTTCATGCACCTACTGTTACAGCTAAAGAATGGCGATAACAACAAGTCTGCAGACACTCAAAGTTTAAC
TAATGATGAATTAATTGCCCAGTAGTTTTATTTTCTTCCTTGCTGGCTTTGAGACATCCTCAACTACAATCACGTTCG
CTTTACTGGAACTAAGTTCGAACCAGGAAATTCAGGATAAGCTGAGGAAGGAAATCAACGAGGTTTTAGATCGAC
ATGATGGAAAAATAACGTACGACGCCATGATGGAAATGAAATACCTGGACATGGTCGTAAATGAATCGCTAAGGA
AGTATCCGCCAGTGGCTATCCTTCCTCGAGTGTGTAGCAAGAACTATGAAGTACCTGGGACTAAGGTAACAATCG
AAAAAGGCACAAAAGTTTCAAGTACCTGTCTGGGGCATACATATGGACCCTGAGTATTATCCCGAACCGGAGAAG
TTTAATCCCGAACACTTCACCCCAGAGAACAAAGCCAACCGACCAGAGATGGCGTTCCTTCCTTTTGGCGAAGGTC
CCCGAATGTGTATGGACTGATCAGTTTATTGAAGAATTTCAAATTCACGGTGAACAGCAAGACCCATACACCCATC
CAAATAGAGAAAGTTAGTTTTGTGTTATCTGTTGAAGGTGACGTTTGGTTGAACGCCTCAAGAGTATAAGTTTTAA
GGATATTTTTGATTAATAATACGTTATTTTGTAATCTATTTTGAAAAAAATAATCCTAAGTAAATCAGATTTTTAATT
TTTTATATTTGTTGTTAATTTTCATAGATTGTTTCATAGTAGATAGTAGATAAAAGTTGTATTAGGTCAAGTCATAAT
ATTTAGAAATATATATACTCGTTATCAACTATATGTGATTTGTATTATATTTATTTAAACAAACACTCTATGTTTGTTT
TAAATTAAATTCGTTAATGGTGTATGTGTTATGTAATGTATATTTTATTTAATTTTTGTTGTATTAAATGTATTTAAAA
TTTATTTATACCTGACAATTTTTAATCCTTCCTATCTTTATTTTATGGACCAAACTGTAATAATTTTGTCACCAGAAAA
ATATATTGAATAAAATATCGACAAA

> RferU5_CYP6CR10

AAGCAGTGGTATCAACGCAGAGTGGCCATTACGGCCGGGGGACTTCGTTCCAATTCACAATCCAAGTAATATTCG
GCATGCTAATTACATGACCACGCATTTATTACATTATTTTATTATTGTTTAGACATATTGTTTATTAGGGTTGGTTGA
ATATTTTTAATTATTTGATATAAGGAATCAAACAGGCAACAATAAGCGACACGTAGAAGAAAACTCAAACCGACTT
AACCACTGCGTCAACAAGCAGTATATTAAGATATGGCCTCGTACATCGTCTCCAAGCATGGTATAGGAAGTGGTTA
TCGCGATGCGGGAGTGATCGCGGAATTGTCATTGTGCTCTGTGCTTTTATACGAACAATGCTGCCGCTATACCTAA
TAATTTTCCTATGTGTGGCAATTGGAATAGTATTTTATTACAAAAGTCTATTCAATTACTGGAAAAAGAAAGGAGT
GCCATATTTAAAACCGACATTGCCATTTGGAAATGCCACTAAATTCTTTCTTAACCAGCAATGTATAGGAGAACTCT
TTATGGATGTTTATCTTCAAATGCGACGTCGAGGATGGAAACATGGTGGGATGTTTTTCTGCGGTAAACCGGTATA
CATCCCGCTGGATAATGAACTTGTTAAAAAGATACTGGTTTCCGATTTTATAAACTTCCCTAATCACGGATTGTATA
TTGACGAATCCGTCGACCCTCTATCGGTACATCTTTTCAATATGGAGGGTTACAGGTGGAAGAATGTAAGGGCAA
AGTTACCTTTGGCATTCACGGCAGCTAAAATGAGACGGAACGTTATCATAATGAATGAGACCTGCAAAGAGTTTTT
GAAAATTATAGATAAATACGAATCTGAAAACAAATCAATCAATATTAAGGATGTGCTCACTCGTTTCTCTATAGATG
TAGTCAGTGCGTGTATATTGGGTATGGAATCCAATACGCTAAAGAATCAAAACCCCGAGTTACTTCGAGAAAGTA

GAGCATTCTTTGACAATCAGTGGAGCAGAGTAAGCAATACTCTGGTTATTCTTATACCTCGACCTGTTTTATCCAAG
CTGAACTTCAAATTATTCACGAAAAGTGCCACCAAATACTTCTTAAACATGTTTAAAATGATTAAAGAACATAGACA
AACTGAAACTACCAAACGAAATGATTTAACTGATTTACTGCTGGACTTGGCCGATAAGAACAAGTCACATCCCGAT
TTTAATGGGGTTGGTACAATGGAACCATTAAACTTTAACGAATTTGCGGCACAATTGTATATTTTCTTTCAAGCTGG
GTTCGAAACTTCCTCGAGTACGCAAGCCTTTGCCCTATACGAACTAGCATCAAATCAAACAATTCAAGATAAACTAA
GGCTCGAAATTAATGAGGTTTTAAAGAAACACAATGGACAGTTCACATACGATTCAGTTAACGATATGGATTACTT
GGGAAGGGTTATAGATGAAACTTTAAGAAAATATCCGATCTTTCCCGTATTACCACGTGCTGCTACTGCTGACTAT
CAAGTGCCAGGAACCGATGTAATAATAGAGAAAAATTCGTTCGTACTAGTTACTAATTTGGGCGTCCACTATGATC
CAGAATACTATCCAGATCCGATGAAGTTTGATCCTGATCGATTTACAGCAGAAAATAAAGCGAAGAGGCCTTATTG
CTCATATATTCCATTTGGGGAAGGGCCAAGAATTTGTATAGGAAAACGATTTGGAATATGGCAAAGTAAAATCGG
TTTGGCTAACATAATCAAGAACTACAAYGTGACTTTAAGTAAGAAAATGAAACTACCAGCCAGGTTCGATACCAGT
GGATTGATCTTGAAATTACACGGGGATATTTGGGTCGATCTTAAAAGGATTTGTTAAAGTTTTATTTAATAGCTATT
AATATTTATAAGTGATTATAAAACGAGATAAATATATGTATATGTAGAGTAATATTTTTTAAATAAATTTAGCGAGG
CATTAAAAATAGCGTCTTGTTTTACATTAGAATAGATTGAAAACCTCACTATAGTTTTGTATTCACTGTTCATGAAAT
CATCAATTCTTTCTGTAAAATAAAATATTATACTACCCAAAAGAAA

> RferU10_CYP6BX6

ATTCTTGAATTGTAATATAAGGAACTTAACTGTATTAATCTGCTTTAGTTATTTTGAGTAATACCCCTCCTTCCACAG
TTGGAATGAAATTATTCACCACGTATTTGGGTGGATAAGGCGTGGTCTCGTGCAAAGAGAAATTAAAGTCTTTCAA
TAGCATGGCCAGTCCAAGTTTAGACTCTAACAGAGCGAACCTCAATCCTAAACACTGTCTAGGCCCATCACCGAAT
GGTATGTATGCGTGAGGGTTGATTTTGTGTTTATTCTCAGCACTAAATCTGTCCGGATCGAATTTATACGGATCAG
GGAAATATTCAGGATCCAAATGTAGACCCCAGACAGATATTTTCAAWGTAGTGCCTTTTTCAACTATCACCTCCGT
GTTGGGTACTTTGTARTTCTTCGTGCAAACTCTAGGTAGGATTGCAAGTGGTGGATACATCCTCAGTGTTTCTTTCA
CTACACTTTCAATGTACGTCATCTCTTGTACAGCTTCATAAGTGAATTTACCACCATGTTTCTTCCATAATGCTCTGG
CCTCTTCTCTCATTTTCTGCTGTATATCCGGATGCTGTGTCAGTTCCAATAAACAGAACGAGATTGTGGTGGACGAT
GTCTCAAATCCGGCAGTGAAGAATAACAAAACTTGACCAGCGAGTTCTTCAAATGATATACCATCTTCTGCAACAG
TTTTGTTATAAACATCATCATCGGTACTGTTAATTTCGGTAANTTTTCCCTTATTCTTTAATTGAATCATAAGATGCAT
AAAGTCCTTTCGCACAATTTTGTTGTCCTCTCTATATTTCAAAGTTTCTGTTACCACATTCCTAAAGAAATTCTCCAGA
TCGAAAAAGAATGTATGGTTTTGTCTGGAAGTAAGAGAAAGGAATTTTGCTATTTTTGTCCATATGGTTGGCCGTT
TACTAGTGGTACCGAACAGTTTCTGTCCGTGTTTGTAAAATTCCGAGTTTTCATCCGTCAATGAGTTACATTCGATG
CCGAATCCGCAAGAACCTATGATGTCAGTGGTGAACCTCATGGAAATACCTTTCAGTTCGATCGGTTTGTCCAACTT
AACTAAATCWTGAACTAAAGTCGATAAGATATCAGCTTTTTCTGTTAAAGTGTCAAACATCATCTTCATTTTCCCGT
TGTAAAAACTGGAGTTAGCTTTCGTCGCATATTCTTCCATTCTTCTCCTCTCATATGGAACAGGTTATTGAAAAACA
GCGTCTTCGGTATTTCGTTAAAGTGGAGATCAAAATACTCATAATCCTTCGTTGATGTTACTTAACTAAAAGTGGAC
GAACCGGCATCCGAAATCTCTGGATGCGATTGTGCTATTTCTGCCGCACAATGGCCGACGGGAGGGGCTCGCAGC
TATCCTAGTTCAAGACGATCAATGCCAAAATCGATTCCAGGCCCGGATAAGACCAAGAGCGACCGGCAGTGCCCG
CGTGATATTCTCAAGAACGGAACGAAGAAGACGCCGCGACCATCTGACAGTCGGTCCGCCGGTCCAAGGTCGGC
GCATACGTTTATTTTAATATTTCGCATCGCGCGTTGGCCAAAATAAACCGCGCCATTACCCATAAATAAGTGCACGT
TATTTAGTTGGTGAAGTGGCATCAAGAGAAGACTCATGGGAAAACATCATGAGTTGTTGGTCAATCTGCGACGGA
GTTCGCCCGACGAGTCTTGGGGTTTCAGCCTCGTGGGCGGCGCAGATGTTAAAACCCCACGGATAGTCAAGAAGG
TATCGTTCGGATCGCCGGCGCAGGGCGTACTTCAATGGGTCGACATCATCACCAAAGTTGGTAATTACGATTCCAG
AGATATTCGGCATCAGGATGCACAAAATTTATTCCGGAACGTTGGCAAACATGCCCTCGTACTCGTACAGATAGAA
AGTGGACCACGTTATAACACCAGCACTGGCTCTTCCAGAAACAGTTCCCACTACAGTCCCCTCTCGGTATCACCCAA
TCTATCGCCCAGAGGAGCCCCTTTAAGTCCCTCAACAGGATACAGTACCGGGGGTTCAGCCCTTACCCCAGTCTAC

ACACCACTAACCCCGATCGACAATAACTATTTCGATTCCTACGAATACGGTTCGAGGAGAGGGGGTCCTGGTCAA
GGTTACAGCCAAGGTCACTACAGCCAAGGTCAGGATCAGAATGGGGAAATTCACGTTACCAAGCAGCCATATCGT
ACTACGCCACTGGTACTACCCGGAGCGAAGGTGAAGAAGGAGCCCGGACCAACAGAGAGCTATTTACGTCACCAC
CCTAACCCCAGTGTACGAGCACCCCCGCAACATTTAGAACCAAAGGTAGCTCATTCACTTTTGGATAGAATATCCAC
AGGTGACCCAAACAAGCAGCTCGTACACAAACAGTTCAACTCACCGATCAATTTATACAGCGAAAGAAACGTCGT
AGACACTATCCACAGGCAAACCGGCGTCCAGGCCGCGCCAAAGCGAACTGTAAAATTCAATCCTGCCGAGAGTGA
AACTTTCAAGTATCTGCAGGAAGAGGAGCACGGAGGTTACGGAGGACCCGTCCAAGAAGTCAACGTACCTCCACA
AAGTAAAATATACGCGCCCACCAAGAAATCAGCCCATGTAGTTAACCAAAATAACCCTCAATACTTAAGCGCTATA
GGCCACGATCCGGAGGTCATCCAACAGAGTGGATCGTTCAAAAGGCTGATGATGTCAGTTCTTCCAGGAAGCAAC
TATTAAGCAACCTTAATACAGTTAATATTTCGGAAAAACACTGAGAAGTCTGGACAAAGTGCAGCAGTCAATAAAA
ATCGACGTATATTGCATGTTTTCCTTGTCTCTCGAAAACTTGCAACATACGTCAAAACATTTACGAAAATTATAAAA
AGAAAACAATCAATCGACGGATATTTTAAAAATATTCGAAAACAATAATATCCTATTTTTTAAATATCTCTTTCAAAT
TTGTGTCCATTTTTATACGTAGAGCCATTTGGGAGTCAGAATCCAGTACCATCTATTTAGGTACATAAGTTTGAGGG
GTATGAAAAAATTAATATAATCTTGTTAATTGATAAGGTAAACTAATATTAATTGTAAAAATATACTTTGTTAGGTT
TTTAAAGGTTTAAAAAACTCGGACAGCCATGACAACTGTCAAAAGCGGCAACCGTCAAGCGTTGATTGATAAATTG
ACAGTTCGACGGGCGGTCCAAAATTTTTGTTGTGTATTTTAAGTTATTTTTGTTTTTGTTTGTTAAATCTTATATTGA
AATTTTACAAACGAGTGAAATCTAGTCAAAATGTGGTTAGTTAGTAAAAGTTTAATTTCTATTTTAATTCGAATTGA
AGTAAATACCTTACATTATCATCTTTTACGTGGTGTCAGGCAAAAGTCGCCCGGAATCAAAGTAAACACTAAAAAA
TAGCTGTAAAATTATTTGTCGCATATAGACCTAGTGTTAAAGTTTATTATTACTATTATTATATAGAGAGAGCTACTT
AAATTGTTTAATTATGTTTAAAGGAAAATAAGGAGTGTTTTTAAAAATGTATTTATTTACTGAAGTTATCTATAAAT
AAAAATAAAGCTATATTTCTTTAAAAAAAAAGAAACACAAAAGACAAACATGTCTGCGCCTCTGTACCATGAATAT
ACCTTGGTCATTGTCAAATATCGCTAGACTGAAGTATGCAATGTTATGATTTGACACACATGTCATTTTATGATACT
GTAATCAAACTGCAAAGCTATACAGAAAGATATTATGCGAATAGTAAAACAGATCATGGATTTATACCTCTTTGAA
AATATCAAGTTTCGTGCCTGAAAACGTGCATTAGCTTAGTACGCACATTTACTTAATCAAAGAAAAAAGCCGCTAA
AAGCCATCGTTTGCTGATACACTTAACTAACCGTAAAACATGTAAGCGTAATCTAAAAAGACCGAATAAAGTCTGA
AGATGCGAAACCGAAACAATTACAAGATGACAAAACTAACCAAAACTCAAAAACAATTGGCAAGAGCATTAAATG
TGGCCCAAAGAACGATTACCAACCATCTACCATTCCCTGCCATCTCTAAAACCTGCTATAGCTCCAA

> RferU14_CYP6NS1

TGTTATTAGGAATATTTATTTTTCTTTTAAAATATCCTATCGGTCTAGAGTGAACAATCGAACAGGATACCTCGTACT
TTTTTAATTCGCCTCATAGAAATAATTTTAAAACATCATATCATAAGTACTTTTGTCTTTTGTCTTTTTGTTGCTGTTTC
TTTTTTTTCGTTGCTACCAATGTCGTATACGTGATATTTCTTACTGAGTCAATAGAGTGTAATCGACGATCCAGTGA
AANTAATGTTTTATCTTGTGATTTTTGCGGTTCTTGTCGGCGTTTTGTGGTATTTGAAGAGGGCGAATCAGTACTGG
AAGAGACAGGGAATCTACCAGGCCGAACCGGAATTGTTTTTCGGAAACAGTAGGCCAGTCGTCTTGCAAGGTAAA
CCATACTGTTATGCCTTACTGGAGAGATACCAGCAGTTTAAAAAAGCCAAACTGAGGTATGGAGGTATTTATCTAT
TCACGATCCCGACACTTATTGTTATCGATCCGGCCCTCATTAAACAAATGNCTTCTNGAAAGACTTCAANCCATTTC
ATGAACAGAGGCTTGTACCATACTCCAAAAGATGTTCTCTCCATGAATCTAGTGAACATCGAAGACGAACTATGGA
GAACTCTTCGCACCAAACTCACACCAACGTTCACCTCCGGTAAAATGAAGATGATGTTCGAAACATTGCTAGAAAA
AACGGTAGGGCTGGAGAGATCTTTCGGGGAATACGCCGATTCTAAGGAGGTCTGTCCGATTAGAGATATCTTGGC
TCGATTCACGACTGACATCATCGGTAGTTGTGCCTTTGATACCCTGGTGGTTGTTGGGAATGTTGAATTATAAAGA
AGACGGCGTTGAAGTGGCTAATTTCTTCAGTAACCTCGTCAAGGACACCATTAGCTACAGAGAAAAAACTGGAAT
GGAAAGGAAAGATTTTATGCATCTCTTGCTGGAACTTAAGAACAATGCATCCGGTCAAATACTCACCGAAAATGAG
ATTATAGCTCAGTGTTACATCTTTTCCTTGCCGGATTCGAGACTTCTTCCACAACCCTTACCTTCGCTCTGTCGGAAC
TGGCACAGCACCAAGACATTCAGTCGAAACTAAGGGACGAAATCGAGACCACCCTGGCGCAACATGGCGGTAAA

CTTACATACGAAGCCGTCATGAAAATGACTTATCTGGAGAAAGTTATCAATGAAACTCTGAGGAGATATCCACCAG
TTCCATTAACACAGAGGGTGGCTACATCGAAGTATCATATACCTGGCACTAACCACGTTCTGGATAAGGGTGTTGT
AGTTCAGATCCCTATCTGGGGCCTGCATATGGACCCGGAATACTACCCCAATCCAGAAGTATTCAATCCAGAGCAT
TTTAGTGAAGAAAACAAAGCTTCCAGGCCTGATTTCACTTTCCTACCGTTTGGCGAAGGACCAAGAATGTGTATAG
GGCTGAGATTCGGTAAACTGCAAACCAAGGTGGGATTAATAAGTCTGATCAGAAAATATAACTTTACTTTGAATGA
GAAAACCAAAATTCCACTGGAGATGGCACCAAGCGAGATTGTTATGGCGCTGAAAGACGACGTATGGGTAAACG
TACGAAGGATTTAATAATACTTGCCTGTATTGTAATTCAATTCATATCGAACGATATTTTTATATAAAATATATGAAA
TATACAGTGTGCCATCAAAAAAAAAAAAAAAAAAAAATCTTAAGTTACTCTGCGTTGATTACCACTGCTTAAAAAAA
AGTAAAAAAA

> RferU15_CYP6BW20

CAAAGGACAAGAACAAATAACGGAACTATTCGTAAACTGACGTAGCGCAGTAATCTGAAATATTACGGTATTACG
TACCCACCGACTGCAACGGGATGCAGTTTGAAACGACACAGCGACTGAAACGGTCGTGAGCGCAATAGCATGTTG
TTTCTGTTTATAAGTGTATTTTTAGTGTGCGTTTGGCTTTTTTTGAAATGGCGCAACTCCTACTGGAATCGCCGTGG
GGTCTTCCAAATAGAAGCCAGGCCCTTGTTTAGTAAAGTGTCTTCGGCGGAAACTATGTTGAAACAATATGCCCGC
GCCAAAAGCCTGAACCACAAATACGGCGGCACATATTTCTTATACGTACCCGTTTTTATTCCCATCGATCCCATTCTT
ATCAAGAATATACTGCAACGAGACTTTATACACTTCCATGGACACGGAACCTATCACCAACCGAAAGACGTCCTCT
CCATGAACCTATTTAACTTGGATGGAGAGAGCTGGAAAAGATTGAGAGCCAAACTTACACCCACGTTCACATCCG
GTAAAATGAAAATGATGTTTGACACATTATTAGAAAAAACCACAGGTCTGAAAGAAGGTGGTTGGTGAATACGCG
GATTCTGGACAAACCTGCGCTATAAAAGATATTCTTGCGCGATTCACAACAGACGTTATAGGAAGCTGTGCCTTCG
GAATCGAGTGTAACTCATTGGAAGAACCGAACAACGATTTTCGGAAATACGGCAAGAAGGCGTTCGAACCCAACA
TTCGCAAATTCTTGAAAGTGTTCCTCATCCCTCACTGGATATTAGCCAAAACCGGTTATAAATTCAGCGGCGAAGAC
GTGACCGAATTCTTCACCAACATGGTGAAAGAAACAATCCGTTATAGAGAGACGAACAATATATACAGGAAAGAC
TTTATGCAATTATTATTGGAGATAAGAAACCAAAAAGAAACGACGGACAGTGTCCTGACTAGTAACGAAATAATA
GCCCAGTGTTACGTGTTTTTCTTGGCCGGTTTCGAAACATCCTCCACAGCTATCACCTTCGCTCTATTGGAATTAAGT
CGGAACCTAGATGTTCAGGAAAAATTGAGACGCGAAATCTTNGCAGGTGTTAAATGAGTATAACGGTACGCTCTG
CTATGAAGCCGTTGGAGATATGAAATATTTAGACATGGTCGTACAAGAGACCTTAAGGAAATTTCCACCAGCGGC
ATCAATTCCCCGAGTGTGCAGCAAGAGGTACAAAGTCCCCGATTCAGATCTAGTCATTGAAAAGGGCGTACGAGT
TCAAATCCCCGTCTGGGGCCTGCACATGGACCCGGAGTATTACCCCGACCCCGAGGTGTTCAATCCGGAAAATTTC
AGCGAGGAAAACAGGAACAAGCGCCCGGATTTCACGTTCTTGCCGTTTGGCGAAGGTCCACGAATGTGTATCGGT
ATGAGATTCGGCCTGCTACAAACTAAAGTTGGCCTAATCAGCTTAATAAGAAATTTCCACTTTACATTGAACGAGA
AAACAAAAACTCCACTGGAGATGGAACGAGGCAGTATCGTACTCGTCGCAAAAGGAGATATATGGTTGAATGTTA
AGAGAATATGATTGCAACATACCTTCAGTAATTTTTACCAAAGTGTTAATGATATTTAGTTATTTTTTAAATAAAACA
TAGATAAAATACAAAAGAAACAGAACAAAAGACAAAACATGTCGGCCGCCTCGGTCTTAGA

> RferU24_CYP6BW21

TGACTTTTAGGAAAACGATGAGATGCAAATAACATCGCTGCGTGTTTCTGTGTAATTATATGATAACATAATTTGTA
TTTAAGTGGTACGGAACTATTCACTTTATAATTTAACAGTTGGTTGTGCTATTAGTCGGTACGAGAGAGTCGTCAGT
GAGAGAGCGCGAATTGTGCGAGATTTTTTTTATCGACTCAGTAGTTTTTACGAACAACAGTTAACAATGATTATAC
CTGTCTTGGCTAGTATTTGTGTACTAATACTGACTATATGGTTCTACATCAAAAATACAATGTCATATTGGTCCAGA
CGCGGTGTGATACAAATAGAAAACAAACATTTTTGGTCCAGGGAATTCTTCGGGACAACAATCCGTGATAAGTACC
AGTACGGTAAATCTTTAAATCAGAAATATATAGGGACCTATATATTCAGTTCGCCAGTCTTTTTCCCCATCCATCCAC
CTTTGATTAAAAACGTAATACAACACGACTTCAGCCATTTCCGCGGTCACGGTAACTACAACGAACCAAAAGACAT
CCTCTCGATGCATCTGTTCAATGTTGAGGGCGAGATATGGAAGAACTTGCGGGTTAAACTGACGCCAACCTTTACA

TCTGGTAAGATCAAGATGATGTTCGAAACTTTGCTGGAGAAAACTCACGGGTTGGAGAAGGTGGTGGGGGATTA
TGCTGACAGGAAGGCGGCTTGCGATATTAAAGACGTGTTGGGTCGGTTTACCACCGATGTGATCGGAAGCTGTGC
TTTTGGTATCGAGTGCAACTCTTTGGAAGAGCCGGATAACGATTTCCGATGCTATGGGAAGAAAGTTTTCGAACG
GGCACCATCTAGATTGTTCTATGTCATTTTTCATACCGCACTGGATACTGATGGCTTCGGATTTTAAGTTTCACGGC
AAGGAGGTCACGGATTTCTTCACCAATCTCGTGAAGAGTACTCTTAAGTACCGCCAGGACAATAACATAGTTCGAA
AGGACTTCCTACAATTACTGATACAAAATGATTATGTAACTGTGGACGAGATCATCGCACAATGCTTTGTATTCTTC
CTAGCTGGCTTCGAAACATCTTCAACAGCTATGACGTTCGCCCTGTTCGAACTGGCCAGCAATCAGCACATTCAGA
AAAAGTTACGAGAGGAAATCGAGGAGGTCTTGAAGAAATTCAATGGAAACTTAACTTATGACGCTGTGATTGAAA
TGAAATATCTGGATATGGTAGTTAATGAGACCTTAAGGAAATTTCCACCAGCGGCATCAATTCCCCGAGTGTGCAG
CAAGAGGTACAAAGTCCCCGATTCAGATCTAGTCATTGAAAAGGGCGTACGAGTTCAAATCCCCGTCTGGGGCCT
GCACATGGACCCGGAGTATTACCCCGACCCCGAGGTGTTCAATCCGGAAAATTTCAGCGAGGAAAACAGGAACAA
GCGCCCGGATTTCACGTTCTTGCCGTTTGGCGAAGGTCCACGAATGTGTATCGGTATGAGATTCGGCCTGCTACAA
ACTAAAGTTGGCCTAATCAGCTTAATAAGAAATTTCCACTTTACATTGAACGAGAAAACAAAAACTCCACTGGAGA
TGGAACGAGGCAGTATCGTACTCGTCGCAAAAGGAGATATATGGTTGAATGTTAAGAGAATATGATTGCAACATA
CCTTCAGTAATTTTTACCAAAGTGTTAATGATATTTAGTTATTTTTTAAATAAAACATAGATAAAATATAATGTTATA
TTTGTATTGTTATATATTCTTTAATATATAAGATTGGGGAG

> RferU25_CYP6NU1

GCACCAAAACACAAAGCAGTGGTATCAACGCAGAGTACTTTTGTCATGTTTTGTTCTGTTCTTTTAGTGCTATTATG
TCTCGTTGCTTTTTACGTGAAGTGGCGTTATAATTACTGGAATAGGCGTGGTCTCCATCAGCTGAAGCCCCAATTCC
CTTTCGGTAATTTTAAGAGTGAACTCATACGTGAAAAATCGTTTTTCGATGGAAACTTTGAATTGTATCAACGACTC
AAAGGCTCCGGTCACAAGCACGCTGGAGTTTATCTGTTCCTGGAACCGGTCTATCTTCCCGTCGATTTATCCATCAT
CAAACGTATTTTGTTCAATGATTTCTCCCATTTTATGAACCGTGGCGTCTACCATCATCCAAAGGATGTGTTGACTAT
GAACTTGTTTAGTATGGAAGGGGAACAATGGAGAGCATTACGGGCCAAGCTGACGCCTACTTTCACCAGTGGAAA
ACTGAAAAGTATGTTTCCAATATTACAGGCTAATATCAAAGTTTTGGATCGAGTTGTTGGTCATAACTCCGTTAACA
AGACACCAGTTGATATTAAAGACGTGGCCTCTAGACTAACAACGGATAACATAGCAAGTTGTGCGTTTGGTCTCGA
GTGCAATTCACTAGAAGAACAAGAAAACTCCTTCCGCACCTACGGTCGAAAGATGTTCGAGCCCCAACCACTGAA
GATGTTCATGATCGAGTTCCTTCCCACTTGGTTACTTGGCTCTCTTGGCTTCAAAGCTCAACGACCGGATGTTGAAC
ATTTCTTCTCCACTACCGTTAAAGACACCATCAAACTGAGAGAAGAAAACAACTTAATGAGAAATGATTTCTTACAG
TTGCTGCTGGAGATGAAGAAGGACCAAACCTTGACCGTGGACGAGATCACGGCCCAGTGCATTATTTTCTTCTTGG
GTGGTTTCGAAACGTCAGCCTCCGCCATCACCTTTACTTTGTTGGAACTGTCACGAAATCCAGGTATCCAAAACAA
GGTTAAACAGGAAATATCCGAGATTTTGAAGGAATTCAATGACGAAATCACCTACGATTCCCTAATGAGAATGAA
GTATTTGGATATGGTCATCAATGAAACTCTTAGGCTATATCCACCATTGCCTGTTCTTCCTCGTATCTGCTCCAAGAC
CTACCGGGTACCGGGAACTGAAGTGGTCATCCAGAAAGGAACCAGAGTAAACATTCCCGTCTGGGGCATTCACAA
GGATCCAGAATACTATCCCGATCCAGAGAAATTTAATCCCGAAAATTTCACCGAACAAAATAAAGCTGATAGACCG
GATACAACTTTCTTACCATTCGGGGAAGGACCCAGAATGTGCATAGCTATGCGATTCGGATTATTACAAACTAAAC
TGGCAATAGCTAGTTTGCTGAAGAGGTATAGGTTCACATTGAACGAAAACACGCATATTCCCATAAAGATGAGGA
ACGACACCTTTGTTTTGATGGCCGAAGACGACATTTGGTTGAATGTTTGTGAAGAGTAAACATGACAATTGTTTTC
GGAAAAGGAACGGTTCCATTTGGAGAGTATTAGTTATCAATTTCAACGAAAGCTTTTGTTGAATATTTTAATGTATT
ATTTATGCGTATTTATATACCGTANAATACAATTTATAATTAAACATAAATAATGGAAGAACAAAAGACAAAAGAC
AAAACATGTCGGACCGCCTACGGTCT

> RferU26_CYP347E6

AAGCAGTGTATCAACGCAGAGTATTTAGATACGTTGACATAGTAGGACGGTCATGATGTTGATTTTGGTTTTGCTG
GGTATTATCTTGGCCCTCTTATGGTATCTACGTAGAAATTCTACATGGGATAAGCGAGGAGTTCCTGGTCCAAAAC
CACTGCCAATCGTTGGGAATATCCTCAGACACTTTTTAGGCCAGAAAACACTTGGACAAATATACCGTCAAATATAT
AACAAATATGATAAATATCCATTTGTCGGCATCTATAGAGCGACAACGCCTTGCCTACTGGTGCGAGATCCAGAAT
TCGTTCAACGAATTTTGGTGAAAGACTTTAAATCTTTTCAAAATAACGAGATGCACGTAGAGAAAGATGTAGATGT
GTTATTTGGCAGGAATCCTTTCGTGTTACGAGGGAAGGAATGGAGGGACACAAGGCAGATGTTATCTCCAGGCTA
TACAAGTGGACGGATGAAGAATTTGTATCCTATGATCACTGAAATAGCAAAGACCATGGTGAATTTTATTGAAAAC
CACCCAACAGGCACAACCGATGGTATCGAAACTAGGCTCTTAACGAAACGTTTTACCCTCGACAACGTGGCTAAAG
CCGCTTTCGGTATAGATGGCAAATGTTTCGGAAGCTATGACGAAATGTCCGACTTTATGCAGCTAACTAATACATTT
CTCCAACCAGGATCTTTCCAGGCTATCCTGACCCAAATGATCCAAATATTTCCTGTCATAACAAAATTGCCTTTCATG
ACTTTTACTAATAAATCCACGGCGGATAAAATCATATCGATAATATCGGAAGTAAAAAAGCATCGATTACAAAATA
ACGTTCAAGCGAATGATTATCTACAGTTTCTGATCGAGCTTGGAAAAGAACACAAATTCAGTGACGTAGATGTGAC
CGCCCACGCTAGTACATTCTTTTTCGATGGCTATTTCACATCTTCCCTAGTTCTGTCTTATCTGCTGCTTTGTCTGGCC
ATGTATCCAGAATATCAGGATAAAGTGCGGGATGAAATAAAGGAAACATTGGAAAAAAATAATGGAGAGATACC
ATATGAATCGCTACATGACATGCCGTGGTTGAATGCTTGCTTATATGAGTCTACTCGTGTGTACCCCATTGCTGAAT
CTATGATGAAAGTTTGTACCGAAGACTTCGAATACACCCCAACAGATCCTAGTTACAAAAACTTAACCGTACAACT
AAAGCCGGGTGATGTGGTCATGGTCCCCTATGGTATTATGAGCAAAGATCCAAAATATTTTGTAGAGCCTGACAA
GTTTTTACCAGAAAGATTGCTGGAGAAAGATGAAATGTCAAACAAAGTATTCTTTCCATTTGGAGCTGGGCCTCGA
GTTTGCATAGGTCAAAGGATGGGTATCATGCAAATTAAGCTAGGAGCAGCTCATATTGTGAAGAATTTCCAGATC
AGTACTAGTCCAAAACTATGCCAACCCATTAAATATTGCGCATATAATTTTATGAATGAAGTTAAAGGAGGAATAT
GGTTGAGATATAGAAAAATAAATGATCACTAATACTAAAAAATATTTAATACGGATTTATATAATAGTATTTTCTAT
TATTGTAATCAACGTTATTTAGTTTTAAATTTAAAGTTCATGATTTACTAAATATATTAGATTTTTCTGTAGCAATTGA
ATAATTTAGCACTG

> RferU28_CYP6BX5

CGACACATCAACAAGTCATCGCACTGTTGTTTACGGTGGGGCTACATCACGGTAGTCAGCAAATTTCATTTAAAAT
CGCTACTCATTCCGATACGTTATCTGGTACCTCCGGGTACTGTTATTTAAGCGGAATGACAAGTCGGCTCGGACAG
TCCCGAAAATGATTTTAATAATAATAGCGGTGGCGCTCATTGCCGTTTATGTGTACAGCAAATATGATCGCACATA
CAACTACTGGAAACGAAAAGGGGTACCCCAACTGCAACCCGTGTTCTTTTACGGTGACGCGAAGGATTTCCTCGAC
GGGAAAATATGCGAAGCGGAGAATACCATGAACTATTACAAGAAATTCAAGGCCAGGGGTCTACGTTTCGGAGG
CGTTTATAACGAGAAACGACCGGAGTGGGTACCGGTTGACCCGGAACTCATCAAGACAATCACGACGAAAGACTA
CGAGTACTTCGACCTCCATTTTAGGAACATCAGAAAAACGCTGTTCTTCAACAACCTGTTTCACATGAGAGGCGAT
GAATGGCGCAACATGAGGAGAAAATTAACTCCCGTTTTTACCACAGGAAAAATGAAGATGATGTTTGATACGTTG
GCAGATAAGGCTGATGGGCTATCAGTACTGATGCATGACTTGGAGAAAAGACAAAAACCTGTAGAACTTAAGGG
GATATCGATGAGATATACCACCGATATTATAGGCTCTTGCGGTTTTGGTATCGAATGTAACTCCTTGAAAGACGAA
ACTTCGGAATTCTATGTACAAGGCCAAAAATTATTCAACCAGACCAGCGTCAAACCTACCTTGCTCCAAAAAATTGC
TCGATTCATCAACGTATTACCATCTACCGCACTTTATGCACTTGATGATACAGCTGAAAAATAAGGGAACCATTACC
GAAATTTCAAACGCAGACGACGATGTCTATGATAAGACAGTTAATGAAGATGGAATCGGATTTGAAGAACTCGCT
GGTCAGGTTTTGCTGTTCTTCACCGCCGGATTTGAGACCTCCTACTACAACAATGTCGTTCGCCTTATTGCAACTGA
CACAGCATCCGGATATCCAGGAGAAAATGCGAGAAGAAGCGAGAAGGATATTCAAAAAATTCGGTGGGAAATTT
ACGTACGAGGGTGTTCAAGAAATGAATTACATCGAATGTGTGGTTAAAGAAACCTTGAGGATGTATCCGCCGTTG
GCTATACTTCCACGAGTCTGCACAAGGAATTACAGGATACCAAATACGGATGTCATCATCGAAAAAGATACGTCGT
TGAGAATCTCTGTTTGGGGTTTACATACCGATCCGGAGTACTTCCCGGAACCGTTGAAGTTCAATCCGGACAGATT
TAGTCCCGAGAATAAGCATAAGATCAATCCTCATGCCTATATTCCATTTGGCGATGGACCACGCCAATGTTTAGGT

ATGAGATTTGCTCTGTTGGAGTCTAAGCTGGGGTTAGCGGTACTTCTAAAAGACTTCAATTTTTCATTACATAAGAC
CACGCCATATCCACCCAAAATACGTGGTCAATAATTATATTCCAACTGTAGAAGGCNGTGTGCTACTGAACGTCAC
CAAAGTTGATTAGGATAGTGAGCAGTGATTACTATGAAGTCGGAA

> RferU29_CYP6NR1

ATAATGTTAATAATAGTTTTGGTAGCAGGTGTGACAATCGGGTTTGTTTCATATTTAATATGGTTGGAGAATTACTG
GAAACGACGAGGAATTCACGGAATCAAGACGGACTTGCTTTTTGGTAATATCAAGAAATTAATAGTCGGGAAAAA
GCCCTTCCCGTGTTCTGTCTTGGATAGATACAGAACCCTAAAAGGATTTAAGTTGAAACATGGAGGTATATTCACG
TATACTAAACCAACTTACATACCAATCGATCCGGTGATCATAAAACACATCTTACAAGCTGACGCGAGACACTTTCT
CAGTAGGGGCTTGTATCACACGCCTGCAGACATCCTCAGTATGAATTTGTTAAATTTGGAGGGCGAGAGATGGAA
ATGTCTTCGAGCCAAATTGTCTCCCACTTTTACCTCGGGTAAAATAAAGATCACGCATCAAATTTTGTTGGAAAAAG
CCGAGCTGATGGCCGCTGTTGTCGGGAAATCCGTTGATGACGATCAACTTTGTGATATCAAAGATGTCCTGTCACG
GTACACTATCGATATAATTGGTAACTGCGCGTTTGGCATCGAAAGTGATTCCTTACAAAATGTTGACGACATCTTCA
TAAGATACGGGAAGAAAATCTTTGAAAGAAGTCGACTGAAATTGGTGCTACTTCAAGTTCTGCCTTGGAACCTCTT
GGCCTACCTCGGATACAGATCGGAAGGACGTGATATCAGCCGGTTCTTCTCCAATTTCGTGGCAAGGATGATCGAT
TACAGGAAGACTAATGCTATATACCGAAAAGACTTTCTTCAACTAATGCTCGACACGAAAATCGATGACCACGGTT
TAACCTTAGAAGAGATCACGGCTCAGGCATATATATTCTTCACTGCCGGTTTTGAAACGTCGTCGAATGCCTTACA
GTTTGCCTTACTGGAACTGGCAGGAAATCCGGAAATACAGGACAAACTGAGGGGTGAAATAGAGGAGGTTTTGT
CGAAGTATCACGGCCAGTTAGTGTATGAAGCTGTTCAAGAGATGGTGTATTTGGATAAAGTTGTGAAAGAAACCT
TGAGGAAATATCCCGCAACAGCCGCTCTACCCAGAGTCTGTACCAAAGACTACAAAATTCCTGGAACGGGCTTGTT
GATTGAAGAAGGACTTCGCGTTCAAATCCCCATTTATGGTATACACATGGATCCAGAGTACTATCCCCAACCCGAG
CAGTTTAATCCCGAAAATTTTAACGAAGAAAATAAAGCAAAACGATTGGATTGTAGTTTCCTGCCCTTTGGAGAAG
GACCTCGAATTTGTATAGGTATGAAATTCGGCCTGTTGCAAGTAAAAATTGGATTAGTAACGTTAATAAGACAATT
CGTATTCGTCTTAAATAACAAGACGATTTTTCCTGTGAACGTTAAAGACAGAGGCGGGCTTTTGACGATAAAAGAA
GACATTTGGTTTGACATCCGTCGAATATGATTTCCCTTTTCCCTTATTATAGACATTATTATTGTTAACAAGTGAAGT
CAGTTAATAAGTTATTTAGTATTAAAATTGCATTGGAATGTGAATTAAATGTGACTGTAT

> RferU31_CYP6NV1

GGCGGGATATATTGGTCTTTCAGTCCAGAGTTAGTTATCATCGATACTAACTTGATGAAGAATATTATACAAAAGG
ATTTCAGTTATTTCCAAGGGCGAGGTTTGTACACGCACAAGAATGATACCCTTTCGCAAAATATGTTTCGACTGGT
GGGTGACCCTTGGAAAAAGTTGAGGGTGAAGTTAACCCCGACGTTCACCTCCAGTAAAATGAAGATGATGTTCGG
CACCGTCCAGGAGAAAGAGAAGCGTTTGAACGACGTGATGGAAGAATATGCTGAGGATGGCGGTATCTTTGAGG
TAAATGACGTCACTCGTCGCTTTACCAGCGACATCATCGGATCCTGCGCGTTTGGTATCGAATCTGATTCTCTGCAA
TGCCCTAAGAGTGAATTAAGAGAATATGGAGACAAAGTGAATGATCCTAAATTACTGAGCATATGGTTAGAATCA
ACTCTACCCGGGACGATATTGAGCTACACCGGCTACAAATGCTACAAACATGTGTCCCAGTTCTACCACAAGGTGG
TAACGGACACAATAAACTATCGTATTCAGAACAAGGTGATTAGGAATGACTTCATGCATTTGCTGTTGCAACTGCG
TCAGAAAGGCTTCCTCGACGATGAGTCAAARACCGGACAGGAAACCGGTAGAATCACGGACGACGATATCGTCGC
CCAGTGCTTCTTGTTCTTCGTGGCTGGGTATGATACATCTGCCAGTACCCTGTCGTATGCTTTGTATGAGCTGGCAC
AACACCAAGACATACAGAACAAGATGCGTGAGGAAATTTGCGAGGTACTGAAAAAATACAATAACCAATTGACGT
ACGATGCCTTGAAGGATCTGGTTTACACAGAGAAAGTGATCTGTGAAACTCTGAGGAAGTATCCAGTGGTTCCCA
CCATCACCCGACGATGCAACCAAGCGTACAAAGTACCCGGAACTGATCTCACCATCGAGAAAGGAACTGGGGTTA
AAATATCAGTTTTTGCCGTACAAAGGGATCCAGAGTATTTTCCAGATCCAGATAAGTTCGATCCAGAGCGATTTAA
CGAGGAAGAAAAGGCTAAACGCCCCGAATATACTTATTTCCCGTTTGGTGACGGTCCCCGGGTTTGTATAAGTAAA
CGTTTTGCCATGATGCAAACGAAGGCTGGTCTAGTGGGCTTGCTCAAGAACCACAAGTATTCGTTCAGTACTACAA

CGCCGACAAACCTGAAGTTCAAGAAAGCCATCTTACTTCTGCATACCTCCCATAAAGTACCACTGACCGTTCAGAA
GATCTCACAGTAAATAATCAACAAAATCAAAATGTGTTCAATATAAATTTGAAAATAAAAATTGCTAGACTGAGGT
TTGGTACAGGATCGAAAAAAAAACAGAACAA

> RferU38_CYP6DG7

CTGATATCAACAGGATATGTGGTGAAACAAAATTAGCCTGTCGTTTATATAAATCTGATACCGTCAGTAGCGGTAG
ACGTTAAGGCGAATACAGTAAGGGCCACTTTTCAACTCACTCGTCTTCGGACGATTTTATTGCGTGAGACACCGCT
GTCTCAGTTAAATAAGACGGGAGAAGTGTGCGGAACTAAAATAATACGCTATGAGCTGCCTTTGTTGGGATGTGT
TGATTGTTCTTGCGGTGATATTTTTAGCTGTGTGGCTTTATTTCCAATATTCTTATCAATATTGGGCATCGAAAGGA
GTACCTTATTTACCGCCAAAATTTCCTTATGGAAACAATAACAGAATATTCTTAAAAAGCACGGCTTTTGCTAGTGA
AGCAAAGGATTGGTATGATGCGTTCAAAAAGCAAGGCCACAAATTTGGTGGAGTCTGGTCATTTTCTCGTAAGGT
TTTGGTACTGGTGGATCCTGAGTACATAAAGGACATTCTCACGAAAGATTTTCAATTTTTCATTGACCGTGATGTAT
TCTCCAACGAAAAGTACGACCCCAAAGGTACTCATTTGTTCAATATTTCGGAATCAGGCTGGAAACCGTTGAGACA
AAAACTCACGCCGACCTTCACCTCTGGAAAAATGAAAATGATGTTTGGCACTGTGTTGGGTTGCAGTAATTTCATG
ATAGATTTTATGAAGAAGTCAACGAGTTCAGGGCTTGATATTGACATCAGAGAAGTTTTAGCGTCTTTTACAACCG
ATGTTATATCGACAGTTGCGTTCGGAGTGGAAAGCAACAGCTTTGACGATTCCCTAAACGATATTCGTCGATATGG
CAGGACTCTATTCCAGAAACTCACTCCGGGACTAGCTCTTCGCGTATTTCTAGGAAGAAGTTTCCCGGAACTTGCA
AAAAAGTTGGCTACGAAAGGTATGCGTCAAGAAACGCAAGATTTTTTTGAGATGATAGTCAAATCTACCATTGATT
ACAGAAGNAAAAACAACGTCTCTCGACCTGATTTCTTACAACTGCTAATGGACATGAATCAAGAACCACAAAACAG
CGACAAATATGTTAGTTTTGATCAAATCGTAGCAAATACTGTACTATTCTTTATAGCAGGATTTGATACATCATCAA
CTACAATGACATTTACATTATATGAGTTAGCTCGAAACCAACAATTGCAAGNAAAAACTCGGACAGAAATCGTTAA
AGNTTTNAAAAAATATGATGGTCAAATTACATATGATTCTCTTCGCGAAATGGTATATTTGCAACAAGTAGTGGAT
GAAGCAATGAGGATATGGCCACCGCTAGTTTCGTTAGGGCGAATATGCACCAAAGATTACATCTCTACCCTCGGTA
TTGGGAGAGATCCTCGAATATTTCCCAGACCCAGAAAAATTCGATCCTCGGAAAGATTCAGTTCGCCTGAAAATAA
AGCTAAACTTCATCCCTATTCGTTCATCTTCCTTTTGGTGAAGGTCCAAGAAATTGCATAGGTTTACGATTTGGTCTG
ATGCAGTCTAAGATTGGCATTATACGTATTCTAACGAATTTTCGTTTAACAATCAGTCAGTCTAAGACCAAAATGCC
TTTAACGGTAGATCCGATTCGTTCCTCCGTTTGAAATCAAACGAAACTGCCTTTATTTATAGAGCTGAAAAACTGTA
AAAATAGTAAACTATTTACATTATTTATTGGATGTATTTGCAATAGATTATTTAATACAANACTATTTAACAGATATT
TATTTAAATATATTTTCAATATTATAAATATAAATATTATATTTAATATGAAAATAATTTAAATATATTTTAATATTAT
AAATATAAATATTATATTAATATGAAAATATAATTATAAATATTATTTAATATTTTAATATAATTAAATATATTTTAAT
ATATATTTAAATATACCAAAGGATATATAGGATTTATATGTTTACTTTTTTAGTATCCAAATTTTCATTAAATGCATC
CTAAAACTTCCTAATATTTAT

> RferU39_CYP6DG2

CAAAATCATTAGAAATAGATTCTCGATTGAATTTAATCATATTTTTCAGTACGAAATTATATTATCGTTTCTATTTAC
CGTCGAAGCTGCTTGAAAAACTATTGAAGAAGGTTACTCGAATACGGCCGTAGATAATTCGTTTACCAGTAAGTTG
CACACAATGGAGCTTAGTGTTCTTGATGTTTTGTTATTTATCGTTGCTACAATTGTAACGATTTGGTTTTATTTTCAA
TATTCGTACGGATATTGGAATAAGAGACAAGTGCCCTATTTGAAACCAGTTTTCCCATACGGTAACAGCACAACTT
TATTACAAAAGGATAGTCTCCCAGCGGGGGAGGTTTATAACTGGTACAATGAATTAAAAAATAAAGGTCACCAAT
TTGGTGGTGTGTGGTCATTCTCACGACCAGTTTTTGTCGTTCTCGATCCAGAATACATAAAAGACATACTGACCAAA
GATTTCCAACATTTTGTCAATAGAGAAATCTACTGCAACGAGAAACACGATCCACTCGGCACGAACCTGTTCAATA
TCGAGGAACAGAGCTGGAAGCTGCTAAGACAGAAACTGACCCCCACTTTTACATCTGGTAAAATGAAGATGATGT
TCAAGAGCATAGTAGATTGTAGCGATAACATGATTAAATTCCTGGAGAAAAAATCTGACGCAGACGAGGATATTG
ATATCAGAGAGGTGTTAGCTTCTTTCACGTCAGACGTAATTGGAAATGTAGCATTCGGAGTCGAGAGCAACAGCTT

CGACGATTCCAAAAACGATATAAGAAAATACGGAAAGGAAATATTCGAAACGTTTGATTTGTTACTTTTATTACGT
ATCGCTGCACAACGAATATGTCCAAACATTTCGAAGAAGCTAGGAATAAAGAGCATGCCGGATAGGATGATCGAG
TTTTTCTCAAATCTGGTCAAAACCACTATGGAGTACCGTACGAAAAACAACATCACCAGGCCCGATTTCTTACAACT
GCTGATCGATATGCACCAAGAAGGTGAAACTAGTGAAAAGTATGTTAACTTTGATCAGATAGTTGCCAATACTCTG
CTGTTTTTTATAGCTGGTTTTGATACATCATCAACGACGATGACGTTTACTTTATACGAGTTGGCCCGCTGTCCAGA
TATACAGGAAAAAAACTCGACAAGAAATCCAAAAAGTTCTCAGCAAACACGAAAACAAACTGACTTACGAAGCTCTT
CAGGAAATGACTTATTTGCAGCAGGTTGTAGACGAGGCCATGAGAATTTGGCCTCCTTTGATTACCTTAGGTAGAA
TATGCACCCGCGATTATAGACTAAGGGATTCTGATATCGTTATCGAGAAAGGTACTCCTGTAGTGATTTCCACTTTA
GGCTTAGGACGCGATCCTCGATATTATGCAGATCCAGAGAAATTCGACCCTGAACGATTTAGCAGTGAAGAAAAG
AATAAGAGACATCCCTATATACATATACCTTTCGGAGAAGGCCCACGCAACTGCATTGGTCTGCGATTTGGTCTTAT
GCAGTCCAAAATTGGCATCATACGTATTTTGACTAATTTCCGTATTCATATTAGTACTAAAACAAAAATGCCACTGA
CAGTCGATCCGTCAATTTTCCTGTTGAAGTCTAACGAAACCCTGTACTTAAAAGCCGAAAAAATGTGAACGGGAAA
AACAACATTTTATAGCCCAGCATTAACCTACATTAGATTAATGATCACAATAATTAGCTTTGAATGCATGCTAGATT
AAACATGAATAAGCGTTTTTTTTTGTTTTGTAATAACCACTGATAACAAAAGTAAGTGTTTTAAATATAAGCGGAAT
AACGACAACTATTTGTAATCTTATATAAACTTATATAGTTTGTCAATATATGTTTTGTACTAAAAAAAACAAAAAAA
AAACAAA

> RferU41_CYP6BW18

TGTAGAATTAAACGAATTTTTTTTTCAGGGCCATTACAAAGGTTAAGTTATAAAAAGCGTGCGCAATTTTTTGGTTA
AAGAAATCATCAAATAATCAAACAAATAACGAAATCATACACATGGAGTTAGTTTATTTTGATAATACTAAGATAA
TATATTAATAGATATTTTGCGCTGTATACTTGTTTCCTGCATAGGCTTGTTTGTATGGAGTACTAGTAGCGGTTGTG
TGTGTGATATTCAGTGATAATGTTGTTATATGTAGTGTTGGCGTTGGTCGCCTTCATACTCTACGTTAAATATAAAC
AATCTTACTGGAAACGCCGGGGTTTATATCAATTCGAGCCTGATTTTTTTGTTGGTAATGGTAAGAACGAGATATT
GGGGAGGCGATCGATTTTCCTTCTCTTCCGCGATATTTACGAGAAGGCCAGAGCTCTGAAACAGGATCATGTTGG
AATATATTTCTTCCTGGAGCCACTGTACATTCCCACAAACCTGAACATCATCAAGCAGATTATGATTAAGGATTTCC
CGTATTTCCAGGGACACGGAGGATTCGCTCACCCCAATGATCGTCTGTCGATGAATCTTTTCAACCTGGAGGGTGA
ACTTTGGCGAAATCTTCGAACCAAACTGACCCCCACGTTCACGTCCGGTAAGATGATGTTTGAGACTTTGGTATCG
AAAACCGACGGTTTGGAGAAACTTATCGATGGTTACATCACCCGCAAGGAACCGGTTGATATAAAAGAAGCCGCG
GCTCGGTTTACCACCGATATTATCGCCAGTTGCGCGTTTGGTATAGAGAGCGATGCCTTACACAACGACAAGAACG
ATTTCCGGGAATTCGGAAAGAAAGTGTTCAAACCGAACCCGCTAGTTTTGTTCCTCGCCAGTACTGTACCGGCATA
CATCCTAGGACCGTTGGGTTTTAGGTTTATCGCTCAGGATGTGGAGGAGTTCTTTATAGAAACCGTGATGAATACC
ATCCAATATCGAGAGAAAAATAAAATAGTACGTAAGGATTTCATGCATCTACTGTTGCAGTTGAGGAACACCGAA
GGCAACAAGGCAACAGACGCTCAGACTTTGACCGACGATGAATTAATAGCGCAATGTTTCGTTTTCTTCCTGGCCG
GGTTCGAGACATCCTCAACCACCATCACGTTCACTCTATTGGAACTGGGTTTGAATCAAGATATTCAGGATAAGTT
GAGGGCGGAAATCAACGATGTCCTGGATCAACATGATGGTCGTATCACGTATGAAGCCATGATGGAAATGAAATA
CTTGGATATGGTTATAAATGAAACGCTAAGGAAGCATCCACCAGCAGCAAGCATATCTCGTGTTTGCAGCAAAAA
GTATGAGGTTCCTGGGACTGGAGTAACGATTGAAAAGGGCACAAAAGTTCAAATTCCCGTCTGGGGCATACATAT
GGATCCCGAATATTATCCCGAACCGGACAAGTTCAATCCCGAAAATTTCAGTGTGGAAAATAAAGCCAAACGACC
AGAGATGGCGTTCATTCCTTTCGGGGAAGGTCCACGCATGTGTATAGGTCTTCGTTTTGGAGTGATGCAAACTAAA
ATAGGACTGATCAGCTTGCTGAAGAACTTCAAATTTACGGTGAATGAGAAAACACGCACACCTATTCAGATGGAG
AAAGTTAGTTTTGTTCTGTCTGTAGAAGGAGATGTCTGGTTGAACGCTTCAAGAGTATAACATAATTCATATATTTC
GGTATTTTCTTTATTATTACTGT

> RferU42_CYP6BW19

CAGTGGGTGCTTGATAAATAATGATGACCTCGATATATATAATTATATCGATACTAGTGTTGATTCTGTGCTATTTA
AGATGGAAATTTACTTATTGGCAGAGGACGCGGGGTAAAACAGGTGACCGAACAATTCATTTTTAGTAAAAAATC
ATTAGGAGAGTCTATTTTAATCCGATACAAGCAGGGCAAAGCAATGAAAGTGAAATATCTAGGGGCTTACTTGTTT
CACGTGCCCAGCTTGCTACCCATCGATCCCGGCTTGATCAAGGATATCATGATAAGAGATTTTGCGTATTTCCAAG
GGCACGGTACGTACCACGAACCCAGAGATATCCTCACGATGAACTTGTTCAGTTTGGAGGGTGAAAATTGGAGGC
ATTTACGAGCCAAACTTACCCCTACGTTCACCTCCGGTAAAATGAAGATGATGTTCGAGACATTGGTGGAGAAAAC
AGGGGGTTTGGAGAAAGTGGTCGGTATGTACGCTGATTCGGGAAAGAGTTGCGCCATTAAAGATATCTTGGGTA
GGTTTACGACAGACATCATTGGAAGCTGCGCCTTCGGCATCGAATGCAACTCGCTGGAAGATCCGGAGACGGAGT
TCCGAGTGTTTGGGAAAAAGGTGTTTAGATTTAACGCACTGAATTTCGTCAATATACTGGTGTTCCCCGCTTGGTTT
CTCGGCAGTATAGGGTTTAAATTTAATGGGCATGATGTCACAGATTTTTTCACGAAAGTGGTGTGCGATACCATCA
GCCACAGGGAGAAGAACGAAGTGGCCAGAAACGATTTTATGCATTTACTGCTTGAACTGAAAAACAAAGATCGGT
CTGTGACCGTAAATGAAATTATTGCGCAATGTTTTGTATTCTTCCTCGCTGGATTCGAGACCTCCTCAACGGCCATG
ACGTTTGCCTTGTTTTGCCTCAGCCAAAATCAAACTGTCCAACAGAAACTTAGAGATGAGATTAATGAAGTTTTGCA
GAAGCACGACGGAAACATCAGCTACGAAGCTGTCAAGGAGATGAAGTACTTGGATATGGTTGTTAACGAGACATT
AAGGATATATCCGCCGCTTCCGGGAATTCCCAGAGTTTGCAGCAAGGATTACCGGATCCCCGGAACGGATTTGGT
GATCGAAAAGGGCACGCGCCTTCAAATCCCCGTCTGGGGCCTGCACATGGATCCTGACTATTATCCCAATCCGGAA
GTATTCAATCCCGAGAATTTTAACGAGGAAAACAAAGCCAAACGCCACGAGTTTACCTTTTTACCTTTCGGCGAAG
GTCCCAGGATGTGCATAGGTCTGCGTTTCGGGCTGATGCAAACAAAAGTGGGATTGATTAGTCTCATTAAAAATTT
CCGTTTTACCCTCAACGAAAAGACCAAAACTCCCGTGGAGATGGAAAGAGGTAGTATCGTGCTGTCGGTGAAAGG
TGACGTATGGCTGGACGTTCAACGGATTTGAGACAATGCTCATTATTCTTTGTTATTATAAAATTCATATATTAATA
AACGTAACACGAAACAAAAAAAAAAAA

> RferU47_CYP6fra2

GGATGATCTCATGCTTCACCGATGTTTAGGATTTCTGTTTTATGTTGCGGCCCTAGTGGTTGTCACATTTTTCTGTAT
ATTTTTATATTTTAAATTTTCTTTTACCCATTGGTACAGAAGAGGAGTTCCGTACATTAAACCTAAAGTTCCTTTTGG
TAATAATAATAGCTTTGGAACTGGTGCATTTTCTTATGGGATCGAAAGCTTAGACTGGTACAGGGAAATTAAGAAG
AGAGGTCTGAAGTATGGAGGAGCGTGGAGTTGGGATAATCCCCTGTTAGTCATCGTTGACCCTGAATATATCAAA
ACAATTTTGGTAAAGGACTTTGACCATTTCACTGACCGTCATTTTTTCACAATCCGAAATACGACCCCAAAAATGAG
AGTCTCTTTGTGGTGGAGAAAGACGAGTGGAAATATATGAGACAAAACTTGAGTCCCACTTTTACTCCAGTCAAAA
TGAAAACCATGTTCGAGGGCGTTAAGAGGTGCACGGTACCATTAATTCAGTGTTTGGAGGAATCGGTCAAAAATG
GAGAAATCATCGACATAAAAGACGTTATGTCGGCTTATACTATAGACGTGATAGGAGCTAACATCTATGGACTTCA
GTTGGAAAGTTTCAAGGCTAAGGACTCGAAGTTTCGCGAAATTGGCCGAGAAGTTTTNCAACATAACCTGGATGA
AAGGGATCAAAGTGATATTTTTCCGCATGTATGAAAAAATTGCTCGAAATCTAGGCATCACCATATTGTCCAGAGA
CATCATTATGTCTTTCGACCAGATCGTCAAGGATACTGTTGAATACAGGAGAAAACGAAGTGAAATATCAAGATTT
ATGCAGTTGTCGATGGATTTGCATGATTCAACAAAGGACAAAGACAGACCGTATACATTCGATCATTTAGTTTCCA
ATATAATTGTTTTTCTTGGCTGGTTTTGATACTTCAGCCACTACCTTATCGTTCTGTTTATATCATTTGGCAATGAATC
CGGAGATTCAAGAAAACTTCGTAATGAAATCATGATAACTTTGAAAGCATGTAATGGTGACTTGACATTCGAGGCT
ATGAAAAATTTAGACGTATCTTAGGCAAATATTAGATGAAACTCTCCGATTATACCCACCAATAAATGCTTTGTCTC
GTGTAACTGTAAAATCGTATAAAC

> RferU55_CYP6fra1

ATGGTAGTAATTCGAAGTTTTCCATCTTTGGCTCGACTATTGGGTTTTGGAGTTTTCCAGATAATGTTACGAGTTTTT
TCGAGGGTGGTTAAAGAGACCATAGAGTATCGAGAGAAGAACAACGTTGAAAGGAAGGATTTCATGCAGCTGTT
AGTGGCGTTAAAAAGGAATGGATCTCTGGAAGCTGGATCGAATTTAAATGAAAATGAGTCAGATCTAGGCAAAGC

GTTAACAATGGACGAAGCTGCCGCCCAAGCCTTTATCTTCTTCTTGGCTGGCTTCGAAACAACGTCCACCACCACCA
GCTACTCTTTATTCGAAATGGCTAAGAATTCGGCTATTCAAAAGAGAGTCCGAGATGAAATATTAACAACCCTGAA
GAAATACGACGGGAAACTGACTTACGACGGGTTGATGGAGCTGAAATATTTGGACCAGGTGATTGGAGAAACTC
TACGGAAGTACCCACCGGCCCAGTGTTCTTACGCGTGTGTACCGAGCGTTATACAGTACCGGGTACCAAAACTACC
ATCGAGAAAGGCCAGACCGTTCTCATTCCTTGCCTCGGTCTGCAAAGAGATCCTGAATATTTTCCTGATCCGGAGA
CATTTGATCCTGAACGTTTCAGTGATGATAACAAGGGAGATAGTCGATTGGTACTTATATCCCATTCGGAGCGGGC
CTCGAATTGTATTGGTGTCGTATTTGTATGTGT

> RferU59_CYP6fra4

AATCTACATATATATAATATATAATAAAGTGGATTTACACCAGTTTTTAGTAGTGTAGCAAAATGTTTCTGATACTA
GTGGCCGTAACGCTTCTTGCCGCTTATATTTACACCAAATATGACAAAACCTACAATTACTGGAAAAGGAAGGGTG
TAGTGCAACTTAATCCCGTCTTTCTATATGGAGATGCCAAGCCTTTTCTTGATGGACAAATATGTGAAGCTGAAAAC
GCCATGTCTTACTATCGAAAATTCAAAGCTATGGGTGTACGATTTGGAGGAGTGTATTCAGAAAACCGACCAGAAT
GGGTACCGGTCGACCCCGAGCTAATAAAAACAATAACAACGAAGGATTATGAGTATTTTGATCTCCACTTTAACGA
AATACCGAAGACGCTGTTTTTCAATAACCTGTTCCATATGAGAGGAGAAGAATGGAAGAATATGCGACGAAAGCT
AACTCCAGTTTTTACAACGGG

> RferC1312_CYP6NT1

AAGTAGAAGCCTTTCCAAACTTAAAAAATGAATTCTACTCTAGTTTTACAATATTTATTAACCTATAGTGGGTTGATT
TTATTGCTTGTTCTATCGGGGATCTATTTATATTTTCAATTTTCTTTCACTTATTGGAAAAGGAAGGGATTTTCTTATA
TAAAACCCAAATTTCCTCTAGGAAACAATAACTGTATTGGCTTAGGTGCATTTTCTTATGGAATCGAAACATTGGAT
TGGTATAGGCAGTTGAAAGAAAGAGGTTTGAAATTTGGAGGAGCTTGGAACTGGGCCAATCCGGTGTTGGTTATC
TCTGATCCAGAGTATGTCAAAGATATACTGGTCAAGGATTTCTCCTATTTTACCGATCGAGATTTCTTCCATAATCCC
ATTGACGACCCAAAGAATGAGAACTTGTTTGTTGTGGAGAAAGACGAGTGGAGATACATGAGGCAACACTTGTCG
CATACCTTCACCTCTGCTAAAATGAAGATGATGTGCCCTGGAGTGCTGAAATGTACCGAACCCTTGTTGGATTATAT
GAACCAGTGCGCCGCATGTGGGAAAGATATTGACGCTAAGGATATCATGTCAGCTTATACGATCGATGTCATTGG
TTCAAACATATTTGGGCTGGAACTGGGTACTTTCAAAGCTGAAGACTCTAAGTTCCGAGAGATTGGTCGTGAAATA
TTCCGCATCAGTGTTATGAAAGGTGTTAAGACTATAATGACGAGAGTTTCCGAAGGCCTTGCTCGAACTATGGGG
ATATCGATTCTACCAGGAGACATAACTGACGCTTTTTCCGAAATAGTTAAAGATACCATCGATTATAGGATAAATA
ATAATATTAAACGACAAGATTTTATGCAAATGGTTATCGATTTATACATGGAAACTAAAAACAGCGAGAGACAATT
TACTTTAAATCACCTGATAGCTAACGTGATCGTGTTCTTTATAGCTGGTTTTGACACATCCGCTACGACACTTCATTT
CGCCTTATACGAGCTGGCGCTTCATCAGAATCTCCAAGACAAAGCCCGAGAAGAAATTGATAGAATCTTGAAAAA
ATATAATGGTGACCTAACATACGAAGCAGTACAAGAAATGACCTATATCAGGCAGATATTAGATGAAACTTTGAG
ACTATATCCACCGTTAAATGTTGTTGCACGTGTAGCAGTTAAACCATATAAACTAAAAAATACCGATTTATGGTTAG
AGAAAGGTAGTAGGGTGGTTATACCAGTCATCGGGTTTCAGAAAGATCCTGAATATTTTCCTGAACCAGAGCGTTT
CGATCCAGAACGATTTTCAGCAGAAAACAAAAGTATTATTCCTTCATATGTCTACCTACCTTTCGGTGAAGGTCCAA
GGATTTGTATAGGAATGCGATTTGGCAGAATGCAGAGCGCAATTGGTTTAATTCAAATCTTGAAGAACTTTAAAAT
CACGTTGAGTCCTTTAACACAAACGCCCATCAAGCCAAAACGGGGCGTATTTATTATGTTGCCCGATGATAGGATT
TATATTAAGGCTGAAAAGATATAACAATAATATAAGTAATATAATGTAATCAGATAAGTTATAATTAGTTACAGTG
TTTTATAATAATAGTACTAACATGAACTCGATGTGGTTCGCTTCTTATTATTAATCTGCAGGGGGTCGTTCTGTTAAT
TATCAAATATCTCTTAAGAAATGCTAAATAATAATATGGTCTAATAAGTAATTTGAATTTTACTTACCAAATCTAAAT
TAAGAGATTGGACCTAAATGGTTACCAAGTACATCATAATGTGATTAGCAAAGCGTGTGTTACGGGGCACTAAATT
GAGTAATCTACGCTCAGCATAGAGCGTAGAACATGGGGCGGCGTAATATATATTGCACATGGGTGTAGAAACAAC
TCAAAATCAGCGTCTGGGTGTTTTCGTCCGCG

> RferC18161_CYP6fra10

GTATGACATTTTCAAGGAACGAGGAGTCAGATATGGCGGGATATATTGGTCTTTCAGTCCAGAGTTAGTTATCATC
GATACTAACTTGATGAAGAATATTATACAAAAGGATTTCAGTTATTTCCAAGGGCGAGGTTTGTACACAAGCCACA
ACACGTAACTCATGAAAAAATCTATTTGAAAAATGTTTTAAATGTCAATTAACATAATTTCAAAAAACTTAAACCGT
T

> RferC18261_CYP6BW17

GTATAATCGAGAGAAAATGTTGTTTTACGCGGTTGTTGTATTAGTTGCCTTCTTGTGGTTTGCTAGGCGTCGACAA
AGTTACTGGAAACGAAGAAATCTGTATCAGATCGAACCGCAATTTATATTGGGGAATGCCAGGAGAGAGCTGATG
GGTAAGCGCTCGTTGCTGGTAATGTTTCGGGACATTTACGAGCAGGCCAGGGCTTTAAAACAGAAGCATGTCGGG
ATATACTTCTTCTTGGAACCTGTTTACGTACCGACGGATCCAACCATCGTCAAACACATTATGGTGAAGGATTTCTC
ACACTTCATAGGACACGGTGGGTTCGCTCATCCTAGTGACCGTCTGTCCATGCACCTGTTTAGTATGGAGGGTGAA
CCCTGGAAACTTCTTCGAACGAAACTCACTCCCACGTTTACCAGCGTTAAGATGAAGATGATGTTTGAGACGTTGT
CGGCTAAGACGGAGGGTCTGGAGACTTTGGTCGACAACTACATCGTTCGAGGGGAACCCATTGACGTCAAAGAC
ATAACATCAAGATTCACGACCGATATCATCGCCAGCTGTGCTTTTGGTATCGAGAATGATGCCATGGGCGAGGATA
AAAATCTCTTCCGCCACTACGGACGAAGAGCAGTCAAACTCAATACCTTACGACTATTCCTAACAAACACTGTACCA
TCTTCAGTACTGGGACAACTAGGCTTCAAAAGTAACGACCATGACATCGACCAATTCTTCTCCAATACCATTATGGA
TACCATAAAATATCGGGAAAGGTTCAACGTATTCAGAAAGGATTTCATGGACCTGTTACTTCAAATGAAACGCGGT
GAAGGGAAATCAAATACCGAACGTTTGACCGAGGATGAAATTATCGGACAATGTTTTATATTCTTCTTGGCTGGTT
TTGAGACATCGTCGACCACCATGACCTTTGCTTTATTGGAGCTTAGTTTACATGAGGATATTCAGGACAGGTTGAG
GACGGAGATTCTGGAGGTCCACGAACAACATGGGGGAGTGCTGACGTATGAGGCCCTGATGGAGATGAAGTACT
TAGATATGGTGATAAATGAAACGTTACGAAAATATCCACCAATAGCAAGTATCCCTCGCGTTTGTAGCAAGAATTA
CAAAGTACCGGGAACCGACGTAACCATCGAAAAAGGCACTAAGGTTCAAATACCCGTCTGGGGCATACACATGGA
CCCGGAATACTACCCGAATCCGGAGATATTCGATCCAGAAAATTTCAATAAGGAAAACAAAGCCACAAGACCGGA
TATGACCTTCTTACCATTTGGAGAGGGGCCCAGAATGTGTATAGGTCTTCGTTTTGGTATGATGCAGACGAAAATT
GGCCTAGTCAGTTTACTGAAAAAGTTCAAGTTCACCGTAAATGACAAGACCCAAGTACCAATCCAAATCGAGAAA
GTCAGTTTTGTGTTGTCTGTGGAGGGCGATGTCTGGCTCAACGCTTCCAGATTATAGATTAACAC

> RferC18569_CYP6fra5

AGAATGATGCCTTGAGCGACGATGAAAACTATTTCCGAAAATACGGCAGGAAAATGTTTAGACCCAGCACTTTCTA
CATGTTGATCTTCAATACCGTCCCATCGTGGATCTTAGGACCAATCGGTTTCAAATTCGCTCCTCCGGAACTGGACA
AATTTCTCACAGATACCATGTTGGACACGATTCAGTATAGAAATAAAAACAACATTCACCGTAAGGATTTCATGAA
TTTACTCTTGCAACTCAAGAGTGGTGAGAATAACGGCGATATCTCTCAGAAAGTGTCGAGAAAATTGACTGAAGA
CGAAATTCTGGCTCAGTGTTTTGGGTTCTTCATAGCCGGGTACGAGACGTCATCAACCACAATCACCTTTGCTCTGT
TAGAGTTGGC

> RferC21894_CYP6fra6

GTGCCTCTTTTGATCAGTATCTCAATAGTAATACTAACTACATGGTTCTACGTTAAACGCACTTTACAATACTGGAC
AAAACGGGGCGTGATCCAAGTAGAAAACAAACCCTTCTGGTCCAGGGAATTCTTCGGAACGGCGATTCGTGACAA
GTATCAGTATGGTAAATCATTAAAGCAAAAATACGTCGGCACCTATATGTTCAACTCGCCCTCCTTCATCCCCATCC
ATCCACCCTTGATCAAAAGCATAATGCAACACGATTTCAGCCATTTCCGTGGTCACGGCAACTACCATCATCCAGAT
CGGAAGAGCGTCGCGTTGAAGGCGAGATTTGGAAAAACTTGCGGGTTAAACTGACGCCAACTTTCACATCTGGCA
AAATCAAGATGATGTTCGAAACGTTGCTGGAGAAGACTCGCGGACTGGAGAAGGTGGCGGGGG

> RferC21995_CYP6CR11

ATTTTATAAACTTCCCTAATCACGGATTGTATATTGACGAATCCGTCGACCCTCTATCGGTACATCTTTTCAATATGG
AGGGTTACAGGTGGAAGAATGTAAGGGCAAAGTTACCTTTGGCATTCACGGCAGCTAAAATGAGACGGAACGTT
ATCATAATGAATGAGACCTGCAAAGAGTTTTTGAAAATTATAGATAAATACGAATCTGAAAACAAATCAATCAATA
TTAAGGATGTGCTCACTCGGTCAGTGCGTGTATATTGGGTATGGAATCCAATACGCTAAAGAATCAAAACCCCGA
GTTACTTCGAGAAAGTAGAGCATTCTTTGACAATCAGTGGAGCAGAGTAAGCAATACTCTGGTTATTCTTATACCT
CGACCTGTTTTATCCAAGCTGAACTTCAAATTATTCACGAAAAGTGCCACCAAATACTTCTTAAACATGTTTAAAAT
GATTAAAGAACATAGACAAACTGAAACTACCAAACGAAATGATTTAACTGATTTACTGCTGGACTTGGCCGATAAG
AACAAGTCACATCCCGATTTTAATGGGGTTGGTACAATGGAACCATTAAACTTTAACGAATTTGCGGCACAATTGT
ATATTTTCTTTCAAGCTGGGTTCGAAACTTCCTCGAGTACGCAAGCCTTTGCCCTATACGAACTAGCATCAAATCAA
ACAATTCAAGATAAACTAAGGCTCGAAATTAATGAGGTTTTAAAGAAACACAATGGACAGTTCACATACGATTCAG
TTAACGATATGGATTACTTGGGAAGGGTTATAGATGAAACTTTAAGAAAATATCCGATCTTTCCCGTATTACCACGT
GCTGCTACTGCTGACTATCAAGTGCCAGGAACCGATGTAATAATAGAGAAAAATTCGTTCGTACTAGTTACTAATT
TGGGCGTCCACTATGATCCAGAATACTATCCAG

> RferC23577_CYP6ABfra9

ATGTACTCCGGCTAAGAAATTACAAACCATCGGGAAGAAACGACTTCATTGACCTGATGTTGGAATGTAAACAGA
AAGGGACTATCGTCGGGGAATCTATCGAGAAGCGAAAGAAGGATGGAACTCCAGAAATCGCGTCCCTTGAGCTG
GACGACACGCTGATTGCGGCACAAGTGTTCATCTTTTTCGTGGCCGGATTTGAGACCTCCTCGTCTGCTACCAGCTA
CACGTTACACGAGCTAGCTTTCCATCCTGAAGTCCAAGTGAAGGTACAGGAAGAAATTGACACAGTTTTGGCCAA
GCATGATAACAAATTATCTTATGATTCAGTCAATGAGATGACTTATTTGCAATGGACGCTCAAAGAAGCTATGCGG
ATGCTGCCTTCGCTCGGGTACATAAGCAGACAAAGTGCGAGGCCGTATACGTTTCCGGAGCTAGATCTGTCCATA
GATGCAGAAGTGGCGGT

> RferC24987_CYP6HY3

CGGGTATTTGGACTTTGGTACCTTTTTCGATAACCACGTCGGTCCCAGGAACTCTATAGGTTTTGCTACATACGCGC
GGAAGGTTTGCTCCTGGTGGATATTTCCGTAGCGTTTCTTTTTTCCGATTTGAACCCTTCAGATTTCGTAACTTATAC
AGGCCGGAAAACTGCTTGTAATCAACTTAGTCTCGCCTTGTAGAATCTAAACAAACACGTTCACACATCCTGGCCG
AAGACCGACAATTCCTCACCCTCTTGTCCGATGCCCACAGAGTACCGTTCAAATTAGCCCTTTGGGCTGGGT

> RferC28096_CYP6AEfra6

CTAAAACAATGGAAAAGGATGCTGATAAAAATCCATTCACGATAATAGGTCACGATTTATTTTTAGAGACAAAAAT
TGGTGGTCTGAAAATGATTGTTCGTTCTATCTGGCCCGCTGTTTTTTACGGATTACGTCTCAGAAATTATACTGAGA
ATATAAGTTCATTCTTCCACAATCTCCTGACAAAAGTGTTCGCGGGACGAAACTATAAACCCACAACAAGGAATGA
TTTTGTTGATTTACTTCTTAATTTATATAACGAAAAATATTTAACTGGTGACAGCATAGAGAGCATGAAAGGAGAA
AACAAGAAAATATCTTTG

> RferC29183_CYP6ABfra10

ATCTCGAAATGATTCTGCCAATTATAGTAATTGCACTCCTCTCGTTATACTACTACAGCACACGAAATTTCAATTATT
GGCAAAAACGCGGCGTCAAACATGATAAACCAATACCAATTTTCGGAAATGACCTCGACCGGAATCTTCTACGGA
AAAGTATCTCGCAAATTGCAGAGGAAATGTACTGGAAGTATCCCAATGAAAAAGTTGTGGGATTTTTTCGTTGCAA
TGCACCTGAATTGGTGATCAGGGACCCGGAAATAATAAAACGTGTACTGGTGACGGATTTCGACTATTTTTATCCA

CGCGGGATCAATCATAAGGAGGTCGTGGAGCCATTGTTGAGGAATTTGTTTTTCGCAGACGGCGATTTGTGGCGG
CTTTTGCGGCAGAGGATGGCGCCATCTTTCACGAGCGGGAAACTAAAAGCAATGTTTCCTCTGATA

> RferC29199_CYP6fra3

AGTGGTGTCATTGGTTGCCCTTTTACTTTATATAAAACATAAACAATTGTATTGGAAACGACGCGGTTTATATCAAT
TTAAACCCGAATATTTCTTCGGCAATGCCAGGAAAGAATTATTCGGTCTAGGTTCCATTTTCCTGTTGTTTCGCGAC
ACTTACGAAAAAGCCAGAGCGTTGAAAGAAAAACATTTCGGAATATATTTCTTCTGGGATCCGCTTTATGTCCCCA
CCGACTTGAACATCATCAAACACATCTTAATAAAGGATTTTCAACATTTCCACGGCCATGGTTGGTTCTCGCATCCC
AAGGATCGCTTGTCGATGAACCTATTCGCTATGGAAGGCGAACCGTGGAGAGTGCTTCGAGCGAAACTCACGCCG
ACGTTTACGTCCGGAAGGATGAAGATGATGTTCGATACTTTAGTGGCGAAGACGG

> RferC30579_CYP6fra9

GAAAAGAAGTTTGTGGGGTTAAAGACGTGTTGGGCCGGTTTACCACCGACGTGATCGGAAGTTGTGCCTTTGGCA
TCGAGTGCAACTCATTGGAAGACCCAGACAATGATTTTCGATGTTACGGAAAAAAAGTCTTTAAACAAGCACCCTC
TAGGTTGATTTATATCATTTTCATACCGCATTGGATCCTAAGAGCTTGGGGTTTTCAGTTTCACG

> RferC33510_CYP6fra8

GGCCTTCCAAGTTAAAGATGTTCATAGAGAGGACATCTCTAGGTTCATAATAGGCGCCGTGCCCTTGGAAATATGC
AAAATCTTTTACCATAATGTCCTTGATCAAGGCAGGGTTGATGACTATCAATCCAGGCGAGTGAAATAAGTAAACT
CCAAGATATTTGATGTTCATCTGTTTGCTTTCCTTATATCGATTTAAGAGCCATTCTCCCAATGTTTTTTGCCCAGGA
GGAACTGTCCCATCATTTGTTTTACCCCGCGCCTCTGCCG

> RferC33511_CYP6ABfra11

CTATGTTTCACCTTATAATATCTCGAGCAGAAAAACTTCAAGCCATAACTGATGAAATAACAGAAAGAGATTATTAT
GACGCAAGAGAGTTGATGGCAAGGTATACAACAGATTTTATTGGTGAATGTGGTTTCGGAATAAATATGGATACG
TTATCTGATGAAAACAACCAATTCAGAAGACTCGGAAAAAGAATATTTCACAAAACGCTTAGGGATAAGATATGTT
TTGCATTAAAATTTATATTTCCAGAATTTGCAAAAAACTTGAACATATTAGCGCC

> RferC34724_CYP6fra7

GTTTGCTCCCAAAGATCTGGACAAATTCTTCTCCGAAACAGTGTTGGATACCATTGAATATCGGGAGAAGTACGAC
GTTAGGCGTAAAGACTTTATGGATTTGTTATTGCAGTTGAAAAATGGGAGCGATGACGGGAAACACCCGCCTGAA
TCCCAAAGATTAACCGTTGACGAGATAATTGCACAATGCTTTGTTTTCTTCTTGGCTGGATTCGAGACGTCTTCGAT
AACAATCACCTTTGCTCTATTGGAACTGGCATTTAGTCAGGATATTCAGGAAAAGTTGAGAAATGAAATCCTC

> RferU21_CYP345J1

AAACAACGCATGTAAAAAACAAACGTACAAAATACTGTAGTTCTGTACAAACAGTACCTAATTGCCACAAAATCGG
ACATTATAGAAAATATTATTGTTTACGCCGAATCTAGAGGTCGATTATTAGTTGTGTACGAACATATATAAAGGGA
ATAGATGCGATGTAAAATAATAGTTCGAGAAGTGAACTTGTGGTGCAACTATATAGTGAATTGCAGATTGGTTAAT
TAAAAGATTGAAAGATGGCGCTCTGGTTAATATTTCTTTGCTGGTCCACCTTAGTGACTCTCTTATTATATCTATACA
TGACTAGACACTACTCCTATTGGAAAAAAAGAAAAACATTCCACATTAAACCAACTCCCTTTTTTGGAAACATCATG
AAATCGATGACACTTCAAAAATCAATCGGCGATGTCATCAAAGAATTATATAACAGCACCGACGAAGACTATATGG
GAATATTCATTATTGATAAACCCGTTCTACTGATACGATCGCCAAAACTTGTTAAGGATATTTTACTCAAGGATTTC
GCCACATTCCCCGATCGGTGTGTTGTTAGTCCCGAGACACCCGTGTTTACATCTGGTAAACTCAAAGCCATGTCTAC
TCTGATAGCGAAGGAGGCCGAACAACTGAATGAATATATAGGGAAACATTGCAACAAACCTAACTTAGAAGCCAA

AGAGATTTGCGCCAAATACTCAACCAATGTTATTGCTATATGTGCCTTTGGTGTCAGCGCAAAGTGTTTCGAAAGT
GAAGATGCCGAATTTAGAAATTTCGGTCGTAGGATATTCGAGTTTTCAGTGGCCAATATGATTAGACATATAGGAT
GTTTCTTCACACCCAACATTATAAAGCTATTTCAAATATCTTTTGTAAAGGACGAAATCTTAAATAGGCTAGGAGAT
ATATTCACTGAGGTGCTACANNNNNNCATTTCCCAAGCAATACAATTCTTTGTAGCCGGTTTTGAGACAGTGTCCG
GTGTTTTAAGTTTTGCTTTATACGAATTGTGTATTAACCCGGACATTCAGGAAAAGTTGCGACAAGAAATTCAGGA
AATCACAGAAAAACAAGGATTAACTTACGACTCTATACAAGAGATGAAATATCTCGACATGTGTGTTTCAGAAACT
TTGAGAAAGTATCCGGTATTACCATTTCTGGACCGTGTCTGCAACTCTAACTATAAACTAGAAGGGACCGATCATG
TGATCGAAAAGGGACAATCGGTCTACATTTCCATGTTCGGGTTGCATTATGACCCGAAATATTTCCCCGAACCACA
CAAATTCCTACCGGACAGATTCCGGGATAAAAATAGCATCAATCAGGACGGCTTTTATTACATTCCGTTCGGCGAG
GGACCTCGAATCTGTATTGGAAACAGATTTGGATTGATGACAGTCAAAATAGGCTTGGCATCGATTTTAACGAAAT
ACAGATTATGCAGGAGCAGCAAAACACCAGTACCTATTAGATTCCAAGTGAAAAGTTTAATTGTGCAGTCAGATGT
TGGAATACCACTAAAATTTGAACCACTGTAATTTTTATAATACTCTGTCGAAATATGTTACTGTATCAGTAACAGTTT
TTACAGTACCATAAAGATATGTGACATGTGTAATCTATGATTATACTAGTTATTTTTAATACACACTGATAACACAG
TTTTCCTTAAACTTAAATATATGTTTTAATATTAAAAATCTTTTGTGATTCTTATTATAAATCAAATAATTTAAACAAT
GTTTATTGAATTATGTGAAAGAGTAAAGCAATGTGTCTTAATAAAAAACAAATTTCTCCTGCAAAAGAAACAGAAC
AA

>RferU43_CYP393

ATCGTATGGTATGTTGACTGTTGATGATGTCCGTTCGCGTGCGAGTGTCTAATAAACAGCAGTTAAAAACATGTGA
ATTAGTGAACAAATATGTCGTTAGATGATTTTTGCTACGAGGAGAAACTCCCATTTGTGAAATACTTAAACGAGAA
GGTGTTGTTATATTGTATTACTCTGCTAGCTTTCGCTGGTATTTTTCTCCTGATATGGGTCATACAGAACTATACATT
CTTCGAAAAAGCCAAAATATTACGTGGATCTCTTCCCAAATTGCCATACGGTAACCTAAACAATGTTGTCAATAAAG
ACAACACCATATACGAAATGTTATGGAAAAATTATAACAGATTCAAAAGAAAGCAGTGCGATATTGGTGGTGTGT
TCTTCTTCACTAAACCTGTAGTGGTGGTGGTCAAGCCTGAATACATACAGGAAGTTCTAGATAATAGTGATAACTTT
AAACAAGTATTTCCTGAACAGGTCAGTCAAAATATAGGTGAAATATTCACGTCGAACTCGGCAAATAGTTTTATTC
AAAAGTACCGAGAATCTTTACGCGGAGTGTTATCAGAAAAATTGCAGAACTATGACTTTAGTACGGTTCTGCGCAG
TCACCTGCTTGAAATGGCATCGCTGGCTTTTGGACTCAATTCACCTAGTCTCGTCGAAGAAATAAACCACATCTTGA
AAGCCAAAACTGACGCAAAACTAAAATACCATCTGGGGTTGTGCTATCCTTTACTCAAAAGTCGAAACAGCTCGTT
GTATCGAAATTTAAATTCACTATTCGTTGATATTACACAAGCCAGGAAAACAAATGATAATTCGTATGATGATATTA
TTCAATATTTTATGGATTTTCAACGGAAATCCTCCACAGAACCTAAAATAATAAGTCGAATATTGTTTGACTTATAC
GCAGATGTTTTAACACACACCTATTCAGTTGTTTTCGCTTGTTTTTGTGAATTGAGTAAGAATGAGGATGTCCAAGA
CGAGCTACTTGGTGAAATACGACGATACAATAAAAAACATTCAGTAATGGATATAGACAGTTTAAAGGAACTTGA
ATATTTGAATGCAGTTGTTCTAGAAACCTTGAGGAAATATCCACCAGTTCCTTTCATTCTGAAAATAGCCCAAAAAG
ACTTTACATTGAATAACGTACAATTCCCCAAAGGAGTTTTAACTGCTATATCAATCCTTGGTATTCATAGGAACCCG
GACAACTATACGAATCCAGAAGATTTCGATCCGGACAGATTTGCTGAACAAACTAACAGTGCCTTTCTCCCTTTCG
GAAATAAAATAGAAGAAAATGTTTTTAAATTGGTGGTATTATTTACCAAACTCCTCATCATTGACACCTTGACGGCC
TTGAAAGTTAGCAGGAAGAATGACTCGAACGCTGTGTTAAAGTTCGACCGCAATCTCATTCCTCTAAGACTTTCTG
AGTCTTATCTATTAACGTTTGAGAATCTTTAGTTGTAAATATGTTGAGAATTGAATAAAGAGACTTTTAAAAGTGTA
AAAAAAAAAA

> RferU2_CYP9CS2

GTCGACCTAGCATGTGTTATTCTTTATTTGTATCTGGTTAAGTGTTAAACACAAGTCCGTCGATGACGCAGACACTC
ATCTGCGCATCGCTTCTACTCCAAAACAAAAACAACATCCANCTGAATAGTGCATAATTCTAACCGTGGGTGCGTG
TATTGTTTCATTATTTTCAGCATCCATATTACTGTTTTGTTTATACCAGCTCGTAAKATATAACTAAGGACTCACCATG

TGGTTTATTTATGTTGGAGTGCTATGTTTATTATCGGTATGGTACTACATTAGGACTTAYCGCGTATGGTCTCGTAG
AGGAGTGAAACAAACCATACCATGGCCGTTCTTCGGGGATTCTGGGATAACTGTCTTCCAGCTAATGAGCGTCCCA
GAGATACTAAACAAAATTTACTACATGTATCCCAAAACCAGRTACACCGGATTCTATCAGTTTCAGGAGGCCCTTTT
GCTACTGAAAGATCCTGAATTAATAAAACAAATCACTATTAAGGACTTCGACCATTTTACCGATCATCGCACTGTTG
GATCTAGCGATGAAATTGATCCACTGTTCAACAAAAATCTCTTCTTTTTGAAAGGGCAAACCTGGAAGGATATGAG
ATCATTACTCTCTGGCTCATTTACCAGTAGCAAAATGAAAATAATGTACAATTTAATAGTCGAAGCTTCTGAAAATT
TCGTTGACTACTTTTGTAGTAAAAACGAAGATTTAGTCGAAGTAGAATTTAAAGACATATTTAGCACAATATACAC
GACGTTATCGCATGCTACATCATATTCGAGAATAACTAAGTTGATTCACTTACGGCAACCCCAAAATGGAATTTTAT
GCTATGGGGCCGTACCAACTAATAGCTAAACGTCCTCGAAAATTAAAATATTAGCGAAAGACGCTTCGGATTTTTT
NATAAACATCATAGACCAAGCCATTAGAATGAGAGAAGAAAATCATATCGTTAGAAAAGATATGATCAATATAAT
GCTTGAAGAAAGAGAAGGAAAAGCTAGGCAAGATGACGAGGAGGTTATAGATTCAGGATTTGCTGTTGTGGAAG
AATCTCAGCATATCACAAAACATCCTAAAAARCAGATGAATCTAACCAACTTGGATATTACAGCTCATGCCATGATA
TTTTTCYTAGCTGGTTTTGATACTGTGTCTACCGCTATCTGTAACTGCTGTTACGAACTGGCTATCAATCCAGATATC
CAAGACAAACTGCGAAATGAAATTAAGAAAACAATGAAAGAACATAATGAGAAGCTAACTTATGAATCAACTTTG
CGTATGAAATATTTGGACATGGTTATCACAGAAACCTTGAGAAAGTATCCAGGACTGTCTGCCACTGAGCGTGTAT
GCGTTAAACCATACACTATCGAACCAGTATACCCAGACGAACAGCCAGTTCACCTAAATGTTGGGGACATTGTCTT
CCTACCACCATATAGTCTCCATCACGATCCCAAGTATTTTGCGGACCCTGTAAAAATTTATTCCAGAACGATTTTCA
GACGATAATAAAACGTCGATAAACAAATATACATATGTGCCATTTGGGATCGGCCCAAGAGCCTGTATCGGGAGC
CGATTCGCATTACTTGAGATGAAAACCTTGTTAATACAATTGGTAAACAAATTTGAAATTGTTCCTGTTCGGAAAAC
GAAAATTCCTATGGAATTATGCAGGAGCACGATGAGCGTTATGTCCARAGACGRGTATTACGCGGCTTTTAAACTA
CTAAAATGAATTAGCATATTGTATTATTCCTTCTATGTAAGTTATAACTGTATTAATAAAAGCCACCAATAAATTTAA
GAAAACTCACAAAAAAAAATTAGAAATTGAAATCCCTC

> RferU11_CYP9Z33

AAACATCACCAGCTGACTGTTTATTCTATGAAAATAATCGACACATTAACCGTGTTCCGTGCTGTAGCGGCTATTTT
TACTGGTTATATAGTTAAGTACTCTTGCTAAGAAGATGTTTTGGCTTGTGTTAGCGGCTATACTTGCCATCTTGATTT
GGTACACTCTCGATAAGCCTTTGAGATATTGGTCGGACAGGGGCGTTAAACAATCGAAGCCATGGTTGATCTTTG
GAGACAGCTGGGTGAATGTATTCCGAACAAAAAGTTTTTGTCGAGTACATAGAATGGGTGTATAATATGCAACCT
AACAGCAGATATTCCGGTATCTACCAATTTTTCACCCCACTTTGATATTACGCGATCCGGATTTGATCAAACAAATT
ACCGTTAAAGATTTTGACCATTTCACTGATCACAGTAAGTTTGTCGATCCTGAAGCTGATCCCTTATGGGGCAAGA
ACTTATTCGCCTTAAAAGGACAACGATGGAGAGAGATGAGAGCAACATTGACTGGAACTTTTACTAGTAGCAAGA
TGAAGGCAATGTTTAGTTTAATGGACGAAGCAGCCGATACTTTGTTAAGTTTTATGGGGATAAAGGCGGCGATAA
AATTGAACTGGAGATGAAAGAAACGTATACCAGATATACTAGTGATGTTATTGCTACCACAGCATTTGGTATTAAG
GTGGATTCCCTAACGCAACCAGAGAACACGTTCTACCTGATGGGTAGGAAGATAACAAACTTTTCAGGAATTTTAA
TCAGCCTAAAGTTTTTTGGATTCTTTCTGGCACCATCGCTTTTCAAGAAATTCAAAATATCTATGCTGGATAAAGCT
GCGTCCAAGTTCTTCCGCGCTACCATTAATGAAACTATCCAAATGAGAGAAGATAAAGGTATTGTACGCCCTGATA
TGATAAACATGTTATTGGAGGCAAGGAAAGGTATCAAGCAAGAAGATAATAGCGATACGATTGATGCTGGTTTCG
CTACAGTTCAGGAATCAACTGATATAACAAAATTCCGCCAGCTTAAACAATTAACAAATGATGATATAACATCTCAA
GCTTTGATCTTTTTTNTCGCTGGTTTTGATGCCATATCAACAGCTATGTGTTTTGCATCTTACGAATTGGCTATGAAT
AAAGATGTTCAGGATAGACTGAGAGAAGAAATAAAACAAACTTATGAGGAAAATAATCGTAAACTAACATATGAG
GCCTTATTAAAGATGAAATATATGGATATGGTAGTGTCTGAATTGATTAGAAAATGGCCGCAAGGAGGATTGTTA
GATCGTATTTGCACCAAACCATATACTATCGAAGCAGTAAGCACCGGACGAAACGCCCGTAACAATTCCAGTTGAT
GGTCTATTGTGGATTCCCGTTTTTGGTATACATAGAGATCCCGCATATTATCCGGATCCCGAGAAGTTCGACCCAG
AAAGATTTAACGACGAAAATAAAGTGAAGATCAAACCGTATACATATATTCCGTTCGGATTGGGTCCGAGGAATT

GTATTGCTTCCAGATTCGCTCTCTTGGAGACCAAAGTACTTGTTGTTTAAAATAGCTGTTGAATTTCGTAGATTAGC
TTTTCTAGATAAGATGCATTTACCATTGAGATTGTCTAGAGGACTCAATATTTCTGTTGACGGTGGATTTTGGTTCA
ATCTAAAACGAATTTCTAGTTAAATTTGTTAATTATTTATTTAGATGACAATGTTACTGCTAATTACTGATAAAGAAT
TTTATATAGTACGTTGTTAATATTTATTTGAAAATAAAAAAGATGAAAATCTAAAAGGATTTTGAGAAATAGAATA
GAATAAAAATTATTAATTAAATGAACCAATAAAAAAGATCAATAAAAAATAATCAATAAAAATACTATTTTTAAATA
AACTAATTTATGTATATTATCAGCATATTATTCTCGTTCTATATCTGTTATTTTATAAGGTTGTTATTTTCCGTTTCTTT
AGCGTTAGTTATGTAAAAAGTTTAATGGTGTAAATAGAAATAGTAGAGAAATGTTTACCTTTTTCGCTTTTTTATTC
TTAGCTGCATTCGGCCTCAAGCCAAGTTTTTGCTTGAATTGTATTTTTTCCTTTCAAAAAAAATATTTTATAGACTGT
TCCC

> RferU12_CYP9ANfra1

GTTATTCAATACTGAATTTTCTTTTCACTTATCTTATCAATTTTGTAATATTATATTGCACAATATGATTTCATTAAATT
AATGTAATTGAAAAATGCAACATATAATGCAAATATAAACCGCAGTTCTGAGTAATGACTAAAAATATATTGTTTAT
AATTATATCGTTCATTAAAAAAGTAGAATAATAATTTAACATGTTATACTATTTGATATTAATAGTGATATTATTTAT
TTATTTCTTATATTCTACTCTATATTCGAAACCGTATTCCTACTGGGAAAAAGAGAGGAGTAAAACAAGGAAGGCC
ATGGATTCTCTTTGGAGATAGTTGGCCATTAATTTTTCGTTTGAAGAATATAGCGGAAAATGCTTCTTATTATTATG
AAGAAATGAAAGATGTTAGATATTATGGTATATATCAGTTTTTAAAACCTAAATTAATAATACGCGATCCTGAATTG
ATAAAATCTGTTACTGTTAAAGATTTTGATCATTTTACTGATCATGTTGCTATGGTAGAGCCAAAAGCTGATCCGCT
ATTTGGAAAAAATTTATTAATGTTGAAAGGAGACGATTGGAAAAGTATGCGATCGATGTTAACGTCATCATTTACT
AGCAATCGCATGAAAACTATCTACAAATTGATTTCAGAAGCTGATGAAAACTTTATTCAATATTTTTTGAATCAACC
TGGGGATATCATCGAAATTGAAGCAAAAGAAACTTTTGCAAGGTATACCATAGATGTGATTGCCACTACATCCTTT
GGTGTTAAAGTTGATTCTTTAAATGATAGAAATAACGAATTCTTTCGCATGGGAAGTGAATTGGCGAATATCAGTT
TCACGTCATTTCTTAAAATCCTACTTCGGCAAAATTTACCAACATTATATAAGATGTTTAAAATAGACAATGTTCAGA
AAAGAAGGTAAACGATTTTTTTACAGCTCTGATTAGTGATACAGTATCAACAAGAGAAAAGGAAAATATATATCGT
CCGGATATGTTACAAAATTTAATCGATGCCAGGAAAAAAAAATGGAGAGAAAGAGAGCTTTACCATTGAGGATATC
ACTGCCCAAGCGGCTATTTTCTTCTTTGCCGGTTTTGACTCGGTTTCTTCAGTAATGAGCTTCACTAGCTATGAGTTA
GCGCTTAATCAAGATATTCAAGACAAACTGCGTAAAGAAATTTTAGACATTTCTGAAGAAAATGATGGGAATATTA
CCTATGACGTCATTAAGCAAATGAAATATTTGGATATGGTAATTTGTGAGGTATTAAGAAAGTGGCCTCCTTTTGTC
ATTATAGGTAGAGAATGTACTAAGACATATGTGATTGATCCAGTTTACCCAGGAGAAAAGAAAGTAGTAGTACCT
AAAGGAACAGCAATACAAATTCCATTATACGCAATTCATCACGATCCAAAATACTATCCCGATCCTGAAAAATTTAT
TCCAGAGCGATTCGGCACAGAACAAAAGCATCAAGATAAATATTTTCCATTTGGTCAGGGTCCAAGAGCATGCTTG
GGATCAAGGTTTGCCTTGATGGAAATAAAAACAATTTTTGTTCACATATTGAAAACATTTAAATTGGTTACAACAGA
AAAAACTCAAATTCCTCTTAAACTAGTGAGGAAGTTTTCTGTGAATGCTCGACCAGAAAATGGTCTTTATATTGGTT
TACAGAAGTTATAGTTACATATTTACATTATTATATATTAAATGCAATTAAGTTAACTGATTAGAGCCAGTTAACTTT
ATTTTTTATATTATACAATCAAGGGAATTGTCAGGTCTAGTTATTTAAGCCCCACATTTTTTATATCTTACTTTGGTG
GACTTTAAAACTAATTATAAATGTTTGGGATTTTGATAAGTTGTTTAA

> RferU13_CYP9Z86

AAGCAGTGGTATCAACGCAGAGTACGCGGGGATACACAACACACATATCCGCATCATCGAAATTAATCGTTGCAT
ATTAGCTGTGATTTGTTTTGTAGTGGTTATACATACTTACATACGTTGTAGTAAAGCAGTGCTAACAAAGAAGATG
ATCACGCTGGGGTTGGCTGCAGTAGTTGCCATCCTGATTTGGTATACTCTTGATAAACCTTTGAAGTATTGGTCGA
ACAGAGGTGTTAAACAATCAAAACCGTGGTTTATCTTGGGAGACAGTTGGGTTAATTTATTACGAACAAAAAGTTT
TTGCGGATATATTGAATGGATGTATAATATGCACCCCAACAGCAGATATTCCGGTATTTACCAGTTTCATACACCGA
CGCTGGTTATACGTGATCCAGAGCTGATTAAACAAATCACAGTTAAAGATTTTGACCATTTCACAGATCACAGGAA

TTTTGTCGACCCTGAATCCGATCCATTATGGGGCAAGAATTTGTTCTCCTTAAAAGGGCAACGATGGAGGGAGATG
AGGGCGACGTTGACTGGGTCTTTTACCAGTAGTAAAATGAAGGCAATGTTTAGTTTAATGGATGCAGCAGCCGAT
ACTTTCGTTAAGTATTATTGGGATAAAGGTGATGAAAAAATGGAGCTGGAGTTGAAAGATACGTATACCAGATAT
ACTAGTGATGTCATTGCTACTATAGCTTTCGCCCTCGAGSTAGATTCACTATTACTCAACCAGAGAACACATTCTATC
CCCATGGGCAGAAAAATAACAAATTTTTCAGGGTTTGTAACGAGTCTAAAGTTTTTTGGATACTTTATGATACCATC
ACTTTTCAAGAAATTCAAAATATCCATGCTGGATAAAGGCGCATCTAATTTTTTCCGCTCTGTCATTAGTGAAACCA
TTAAAGTAAGGGAAGATCAAGGTATTGTACGACCTGATATGATAAATGTGCTACTGGAGGTTAGAAAAGGTATTA
AACAAGAGGATAATAGCGATATAATCGATACTGGGTTTGCTACAGTCGAGGAATCAAGTGATATAACAAAATTCC
GTCAACTTAAAAGTTTAACAAATGACGATATAACGTCTCAAGCTTTGATCTTTTTCTTTGCTGGATTTGATGCAATAT
CAACAGCCATGTGTTTTGCATCTTACGAATTGGCTGCTAATAAAGATGTTCAAGATAGACTGAGAGAAGAAATAAA
ACAAACTTATGATGAGAATAATGGCAAACTAACCTATGAAGCTTTGTTAAAGATGAAGTATATGGATATGGTAGTA
TGTGAATTGCAAAGGAAATGGCCGCAAGGGGCACTGTTAGACCGTGTTTGCACAAGGCAATATACCATCGAAGCA
GAAAGACCCGATGAATCGCCTCTAACCATTCCAGTTGGGCAGCTACTGTGGATTCCTGTTTTTGGAATACACAGGG
ATCCTTCATATTATCCAGATCCAGAGAAGTTCGTTCCAGAAAGGTTCAGCGACGAAAACAAAGCCAAAATCAAGCC
GTATACCTATATTCCGTTCGGATTGGGCCCGAGGAATTGTATCGGTTCCAGATTCGCTCTTCTCGAGACCAAAGTTT
TATTGTTTAAAATGCTATTGAACTTTGAGATCAGCTTGTCAGATAAGATGCCCGTACCATTGAGGTTAAGCAAGGG
ACTCAATATTTCGGTAGATGGCGGGTTTTGGTTCAATATAAAACGAATTTCTAGTTGAGTTTGTTGATTTCAATGAA
AATATTACGATGTATCTAGTTTAATGCTAATTGATAATAAAGAATGTTTGTTTTGTAAAAAAAAAAAATTTATTTATTT
ATTATTT

> RferU19_CYP9Z82

CATGCGATTTCGCAATATTTTTCAACCTCAATACTGCCGCTACATCTGTTGGATGCCGCCGACTCGTTGACTCAGAA
CGAAACAGATTACAAGCGTTCGATGGCTTTCTGCGCATTCAACTCGCACAAAAGATAAAACAAAATCATGAACTAG
TGGAATATAAACAAAATCACCAGCTGAGTGCTTGTTGTGTATAAGTGATCGACGTGAAGGTGTGTTTTGTTTAGTG
GTGATAATATCATATAATAACACACAGTGAAGTGCTATAGTTCANNNNNNNNNNNNNNNNGTCAAACAATCGAA
GCCATGGGTGTTTTTCGGCGATAGCTGGGTGACTATCTTCAGGAGACAAAGCTTCAGCGAATTCGTCCAATGGGTC
TACAATATGCATCCTACCAGCAGGTATTCTGGTTTTTACCAGTTTTACACGCCCACCTTAGTTCTGCGTGACCCCGA
ACTGATTAAGCAAATCACCGTCAAGGATTTCGATCACTTCACTGATCACCGAAGTTTTGTTGATCCCGATGCTGATC
CATTGTGGGGCAGGAATTTGTTCTCTTTGAAAGGGCAGAAATGGAGGGAGATGAGAGCAACGTTGAGTGGTTCTT
TTACCGGTAGTAAGATGAAGACCATGTTCAATTTGATGAACGAGGCTGCTGATAATTTCGTTCAGTATTTCTTGGA
CAAAAATGAAGATCTAATCGAACTGGAAATGAAAGATACTTATACGAGATATACTAATGATGTTATTGCAACCACT
GCCTTTGGTATCAAGGTTGATTCATTGACCCAGCCAGAGAATACTTTCTACATGATGGGCAAAAAAATGACGAATT
TCTCCGGAATTCTGACCAGTCTGAAATTTTTTGGTTATTTTCTAATACCATCGCTTTACAAGAAATTGAAAATATCCA
TTTTGGATAAATCGGCTTCGGAATTCTTCCGAACTATCATAAATGAAACCATCCACACCAGAGAAGAACAGGGTAT
TGTTCGCCCTGATATGATAAACATGTTATTGGAAGCCAGGAAAGGCATCAAACAAGATGATAACGATACAGCTCA
GGATAATCAAGATACCGGATTTGCTACTGCTCAGGAATCACATGATATAACTAAATTCCGCCAACTTAAAAACCTG
ACAAACGACGATATCACCTCACAAGCTCTAATCTTTTTCTTCGCTGGCTTTGACACGATTTCAACGGCCATGTGCTTT
GGTTCGTACGAGTTGGCAGTTAATAAAGACATACAAGATAGATTGAGGGATGAAATTAGGACAACTTATGAGGA
GAATAATGGCAAAATTACATACGAGGTCTTGTTAAAGATGAAGTACATGGACATGGTTGTGTCTGAGATATTACG
GAAATGGCCTCCAGCTATAGGATCTGATCGAGCATGTACTAAACCATACACTATTCCAAGCCGATTACCTGATGAG
CCACGACTCACCATCGAGAAGGATGTAATGATCTCTATTCCAATAATAGGATTACACAGAGATCCCGTTTACTATCC
CAACCCGGATACATTCGATCCTGAGCGTTTCAATGGAGAGAATACGATTGAAGAATTTGCTTATATTCCATTTGGA
TGCGGCCCAAGGAAATGTATAGCGTCCAGGTTCGCTTTAGTGGAACTGAAAGCTTTGTTGTTTAAATTTCTTCTACA
TTTTGAAATTTTGCCTGGCAAAAAGTTACAGTTGCCTGTAGAATTGGATAAGTCGTCCTTTAATCTATTAATAAAAG

GAGGATTTTGGTTTATTTTAAAGAAAACAACACCATAATAATAAGTGTATAATAAATTACTTCAGACAGCTAATATA
TTTTTTTAATTAAGAAGTATTTGTTTGTTATATTATATTTAGATTTTATTTATATTTTAGGTATAACTATTGGTTCATTT
ATAATTTGATTTTGTATATTTTGACAATTATAAAAATGTTATTAAAATTAGTTTAAGCATTTAAGCTGTGACATTAGT
GTTAGTACCTAATTTTTTTTTACTTTAAATAAAGATATCTTGAAGCTTTAAAGAAAACAGAAACAAAAGACAAAAAC
ATGATTGGCCGCCTCGGATCATAAAAAACAAAAAGTACGTATGCTGATGCTTCTGATTCTAATCTAATTCGATTTTA
TCTATTACCCTTTGTGTTTGTTTTATTTTGTTTATTCTCTTATACAGAATATGATTATTCATATATATGACGCAAGA

> RferU20_CYP9Z83

AAGCAGTGGTATCAACGCAGAGTACGCGGGAGAACGAAACAGATTACAAGCGTTCGATGGCTTTCTGCGCATTCA
ACTCGCACAAAAGATAAAACAAAATCATGAAGCAGTGGTATCAAAGCAGAGTCCCATTACGGCCGGGGGTTGTGT
ATAAGTGATCGACGTGAAGGTGTGTTTTGTTTAGTGGTGATAATATCATATAATAACACACAGTGAAGTGCTATAG
TTCACACTAACATGATTTGGATCATATTGGCAGCAGTGCTTGCTGCCCTCATGTGGTACGTCTTTGTTAAACCTCTG
CACTATTGGTCAGACCGGGGTGTCAAACAATCGAAGCCATGGGTGTTTTTCGGCGATAGCTGGGTGACTATCTTCA
GGAGACAAAGCTTCAGCGAATTCGTCCAATGGGTCTACAATATGCATCCTACCAGCAGGTACTCTGGTTTTTACCA
GTTTTACACGCCCACCTTAGTTCTGCGTGACCCCGAACTGATTAAGCAAATCACCGTCAAGGATTTCGATCACTTCA
CTGATCACCGAAGTTTTGTTGATCCCGATGCTGATCCATTGTGGGGCAGGAATTTGTTCTCTTTGAAAGGGCAGAA
ATGGAGGGAGATGAGAGCAACGTTGAGTGGTTCTTTTACCGGTAGTAAGATGAAGACCATGTTCAATTTGATGAA
CGAGGCTGCTGATAATTTCGTTCAGTATTTCTTGGACAAAAATGAAGATCTAATCGAACTGGAAATGAAAGATACT
TATACGAGGTATACTAATGATGTTATTGCAACCACTGCCTTTGGCATCAAGGTTGATTCATTGACCCAGCCAGAGA
ATACCTTCTACATGATGGGCAAAAAAATGACGAATTTCTCCGGAATTCTGACCAGTCTGAAATTTTTGGTTATTTTC
TAATACCATCACTTTACAAGAAATTAAAAATATCCATTTTGGATAAATCGGCTTCGGAATTCTTCCGAACTATCATA
AATGAAACCATCCACACCAGAGAAGAACAGGGTATTGTTCGCCCTGATATGATAAACATGTTATTGGAAGCCAGG
AAAGGCATCAAACAAGATGATAACGATACAGCTCAGGATAATCAAGATACCGGATTTGCTACTGCTCAGGAATCA
CATGATATAACTAAATTCCGCCAACTTAAAAACCTGACAAACGACGATATCACCTCACAAGCTCTAATCTTTTTCTTC
GCTGGCTTTGACACGATTTCAACGGCCATGTGTTTTGGTTCGTACGAGTTGGCAGTTAATAAAGACATACAAGATA
GATTGAGGGATGAAATTAGGACAACTTATGAGGAGAATAATGGCAAGATTACATATGAGGTTTTGTTAAAGATGA
AGTACATGGACATGGTTGTGTCTGAAATTTTAAGGAAATGGCCGCCTGTTATAGGCACTGATCGTGTTTGTACAAA
ACCATGCACCATATCGAGCCAGTTACCTAACGARCCACTACTCAACATTGAAAAAGAAGTGATGGTATCTATTCCG
GTATTGGGACTGCATAGAGATCCCGYTTATTACCCCAATCCAGAGATATTTGATCCCGAACGCTTCAATGGAGAGA
ACACAATTGAAGAGTTTGCTTATATTCCATTTGGTTGCGGTCCAAGGAAGTGTATAGCATCCAGGTTTGCTTTGGT
GGAATTGAAGTCTTTGTTGTTTAAGTTTCTTTTAAAATTTGAGATTTTACCTGGCAAGAAATTGCAGTTACCTATAA
AATTGGATCAAGCGTCATTTAATCCACTAATAAAAGGAGGATTTTGGTTTATTCTAAAGAAATCGACACAATAATAT
TATTGTAGTTTAGTGTATTTTATATCTGTAGTGATTTTCTTAGTATGGAATAAATTATTTCAGGTAGTAAAAAGAAG
ACAAAAC

> RferU33_CYP9Z85

CAGAAGGATAACGAATTTCTCCGGACTCTTGACTAGTTTAAAATTTTTGGGATATTTACTAATACCAACAGTTTACA
AGAAATTGAAAATATGTATTCTGGATAAAATTGCGTCGGAGTTCTTTCACAGAATAATCAATGAAACTATCCGGAC
TAGAGAAGAAAAAGGTATTGTACGCCCGGATATGATCAATATGCTGCTGGAAGCAAAAAATGGCATTCAACAAGA
TTATCAAGATGTAGTTCAGGATTATTCTGATAATACTTTTGCTACTGTTCATGAGTCAAATGATATAACTAAATTCCG
CCAACTGAAAAGTTTAACCAATGAAGATATCACGTCACAAGCTGTAATCTTCTTTTTTGGTGGATTTGATACTATAT
CAACGGCCATGTGTTTTGGATCTTATGAGTTGGCGATCAACCAAGACATTCAGGATAGGCTGAGGAATGAAATCA
AAAGAACTTTCGAAGAAAATAATGGTCAAATTACGTACGATGTATTGTTAAAGATGAAGTATTTAGATATGGTGGT
ATCTGAGATCTTGCGAAAGTGGCCACCAGTATCTAGAACAGATCGTGTTTGCACCAAGCCCTACATCATCGAAAAT

AAATCTCCTGGGNAACCCAAGCTTTATATCGATAGCGGCATTACCATTTCCATACCAATCATGGGCTTTCATAGAGA
TCCACTTTATTATCCGGATNCCAGAAAAGTTYGNATCCTGAACGATTCGACCAAGCGGATAAAATTATGGATTATC
CGTACTTTCCTTTTGGATGCGGTCCCAGAAAGTGCATCGCATCCAGGTTTGCTTTAATCGAATTGAAGGCGTTATTG
TTTAAGCTCGTTTTACATTTTGAGATAGTACCGAGCAGTAAACTACAGCAACCCATTCAAATAGCAAAAGGTTCGTT
TACTCCTGCAATAAAGGGGGGATTTCTATTTAATTTAAAAAAGCTTTCTCTCTGAATATTTTTACAAAAAATTTGTTT
TATTAACTGGCGTATCCCACTGTAAGTTTAGAAATATTAGTGAATACATTAATTAATAGTTTGTATTTGAAATCGTT
GAATGTAATAAATGGTTATGCAAAAGAAACAGAACAAAAGACAAAACATGTCGGCCGCCTCGGTCTCTAGAT

> RferU48_CYP9Z84

CAGAAAGATAAATGGCAATTATGTGTTATACTGGTGGAATAAAAACATCACCAGCTGACTGTTTATTCTATGAAAA
TAATCGACACATTAACCGTGTTCCGTGCTGTAGCGGCTATTTTTACTGGTTATATAGTTAAGTACTCTTGCTAAGAA
GATGTTTTGGCTTGTGTTAGCGGCTATACTTGCCATCTTGATTTGGTACACTCTCGATAAGCCTTTGAGATATTGGT
CGGACAGGGGCGTTAAACAATCGAAGCCATGGTTGATCTTTGGAGACAGCTGGGTGAATGTATTCCGAACAAAAA
GTTTTTGCGAGTACATAGAATGGGTGTATAATATGCAACCTAACAGCAGATATTCCGGTATCTACCAATTTTTCACC
CCCACTTTGATATTACGCGATCCGGATTTGATCAAACAAATTACCGTTAAAGATTTTGACCATTTCACTTATTCCCTT
AAAAGGACAACGATGGAGAGAGATGAGAGCAACATTGACTGGAACTTTTACTAGTAGCAAGATGAAGGCAATGT
TTAGTTTAATGGACGAAGCAGCCGATACTTTTGTTAAGTTTTATGGGGATAAAGGCGGCGATAAAATTGAACTGG
AGATGAAAGAAACGTATACCAGATATACTAGTGATGTTATTGCTACCACAGCATTTGGTATTAAGGTGGATTCCCT
AACGCAACCAGAGAACACGTTCTACCTGATGGGTAGGAAGATAACAAACTTTTCAGGAATTTTAATCAGCCTAAAG
TTTTTTGGATTCTTTCTGGCACCATCGCTTTTCAAGAAATTCAAAATATCTATGCTGGATAAAGCTGCGTCCAAGTTC
TTCCGCGCTACCATTAATGAAACTATCCAAATGAGAGAAGATAAAGGTATTGTACGCCCTGATATGATAAACATGT
TATTGGAGGCAAGGAAAGGTATCAAGCAAGAAGATAATACCGATACGATTGATGCTGGTTTTGCTACAGTTCAGG
AATCAACTGATATAACTAAATTCCGCCAGCTTAAACAATTAACAAATGACGATATAACGTCTCAAGCTTTGATCTTT
TTCTTCGCTGGATTTGATGCGATATCAACAGCTATGTGTTTTGCATCTTACGAATTGGCCATGAATAAAGATGTTCA
GGATAGA

> RferU6_CYP4G185

GTTCACTCGAACATAGACAGCATAGCCCACGTTTTTAAAGAGAATAACTATGATTGGGGCGCTTGAGGCTGTCTCG
ACTTCACCAATTGTTCTTCCGAACTTTTACATCACTATCCTGCTAGGAGTGACGTTGGTCGTTGCTTCGCTTTATTTA
TGGTCTCAATCGCTGCGACAGGCCAAATTGACGAGCAATGTTCCCGAACTTCCAGGCCGGACGCTGTTATCCACAG
CGTTAAATCCAAGCGATTTCCTGAAGAAAATGCTTGAGGCTATACATGATTTGAAATGTGATGTGGGAAAATTCGG
GATGGGTCCCTATTTCTTCATGGGACTGAAAGATCCTGTAGATGTAGAGCTTATTCTGGGTAGCCAGGAGCACTTG
GAAAAATCGAGGGAGTACGGCTTGTTCGAACCATGGCTAGGAGATGGTCTCTTAATCAGCAAAGGCGAAAAATG
GAGGACCCACAGAAAAATGATCGCCCCAACTTTCCACTCGTCCATATTGAAGTCCTTCTTCCCTGTTTTCAACAAGA
ACGCCAACAGGTTGCTGGAGAAATTGGAGAAAGAGAAAAACAAAATTTTTGACGTGCATGATTACATGAGTGGTG
CTACCGTGGATACTCTTCTAGAAACTGCCATGGGCGTTGAAAAGACAAACAAGGACAATACTGGGTTTGAGTATG
CCAAAGCTGTTATGGATATGTGTAATATCTTGCATCAAAGACATTACAAAATCTGGCTGAGGCCCGATTTTCTCTTT
AAATGGACGAAAATGGCTGCCGAACAATCAAAACTATTAGACCTTATTCACAGCTTAACTAGAGAAGTAATTAAAC
GTAAGAAGGTCGACTATTTCGAAAGGGAATCTAAGGGGGAGAAGAGCTTGTACAAGGAGGCTGTGCGCGCTGAA
AATAAGGCCTCGAAGAAAAACGCCAAGACTGAAAATATTTCTTATATCCATGACGATTTGGAGGATATTGAAGAA
AATGATATAGGAGAAAAGAAGAGGTTRGCATTCCTCGACTTTATGATTGAAGCTAGTAAGACGGCCGGTAACAAC
CTCACCGATGAGGAAATCAAGGAGGAAGTTGACACAATTATGTTTGAGGGACATGATACTACGGCAGCTGCATCT
AGTTTCGTGCTTTGTCTGTTGGGAATACACAAAGACATACAACAAACCTGTATAAATGAGTTAAAAGAAATTTTCC
ACGATAGTTGGGAGAGGCCAATCACTTTTAACGACACATTGCAAATGAAATATCTTGAACGAGTAATCATGGAAA

CTCTGAGAATGTACCCTCCAGTGCCACTAATTTCAAGAAAAGTAAATCGGGATGTTAAGTTAGCCTCAAACAACTA
CATCATACCAAGCGGTACAACCGTAGTTGTATCACAATTCATGACCCACAGACACCCCAGATATTGGAAAAATCCC
GATGTCTTTAATCCAGATAACTTCCTACCAGAAAACTGCCAAAACAGACACTACTATGCCTATTTTCCATTCAGTGC
TGGACCTAGAAGTTGTGTGGGACGTAAATATGCCATGCTGAAACTGAAGGTGATAATTGCCAGTGTCCTGAGGAA
GTATCAGATAACGTCCCCTAAGGCAGAATCCGAGTTCAGTCTGCAAGCTGATATCATTCTGAAGCGTACAGATGGA
TTTAACATTAAAATTGAAGATCGAGTTTTCGCTTAACAAATAGAAAGTACATAGTGTATTATAAATAGTCTATGTTT
AGGAACATTGTATTGTAAAAAGTGGAATAAATGTTATGTTGTAAGCAAAAGAAACAGAACAAAAGACAAAACATG
TCGGCCGCCTCGGCCTCTAGAAT

> RferU7_CYP4BD13

AAGTGTATTTACCGGTGTTGAAGACCTATCTGGATGAATACGGAGATACTGTACTTATTCACGATGGACCATTTAG
TCGGTTATTAATCACCGTTGATTATGAATTGACTGAATATTTGTATTCCTCTACGGTAGAAATCAATAAATCTGATC
AATACAAAATTCTGCATGGATGGCTTGGAGATGGACTTCTGACAAGTTCTGGTAATAAATGGAGAAGCCGAAGAA
AAGCAATTACGGCTTCTTTTCACTTTTCAATCCTTCAAGAGTTTATTCACATATTTGAGGAAGTCGGCATGAAACTA
ACTAAAAAAATTAGTCTGAAATACAAAACTAAAAGAGCATAGATGTGACTAACGTGGTATCGCTTTATACTTTGGAC
GTAATATGCGAGGCAGCCATGGGAGTTCAACTAAACGCTCTGGATGACGATACGTCCGTTTATGTCAAAAACTTG
AAAGATCTATGCGCTATATTAGCAAGAAAAATATTTTCACCTCTTGATTCTAAATTATATCCCCTTACCTGGATGCAC
CACAAGGAAAAAAGCATTTAAAAGTAATTCATCGATATATCGAAGATGTGATACGACAAAGAATCGAAACAAAGA
AGCAAGAAAGTTTAAATTATCACGAAGATAACACTAACGATAAAAGTCGAAAAAAGCTGGCTTTCTTGGACCTTCT
ACTAGAAAAAAAGAAAAAACAAAAGTACTCTGCGTCAATACCACGACCTAAGGGACGAGGTTAATACATTCATGT
TCGAAGGACATGACACTACTGCAACAACAATTTCGTTTTGTCTATATATGTTGGCAAACCATCCATCTGTACAAGCA
AAAGTATTAGAAGAGCAGCACGCCATATTCGGAGAAGATTTGAAGAACGCAAGAGCTTCATATTTAAACCTTAAT
GAAATGAAATATTTGGAGATGGTTATTAAAGAAACGTTACGGTTAATACCTCCAGTTACTTTTATAGGACGGAAAC
TAACAAAAGATTTAGAATTCAAGGGCACGTTATATCCGAAGGACTTGAATATTTTCTTAATCATTTATTATGTTCAG
CTAACTAGTGAATACTTTGAAGAACCTGAAAAGTTTAATCCTGATCGATTTGCTTCTTATGATAAAAAGATGCCCTA
TGCATATACACCATTCTCAGCTGGCTCCAGAAATTGTATAGGACAAAAGTTCGCTATGTTGGAGATGACATCAGTT
TTATCTAAGATAGTGAGAAAACTTTGAGTTACTACCAGCTATTCCATCCCATAAACTTTCTCTAGCCGCAGAGATTT
CTACTAGTGTCTAAAACAGGAATTAAAAATTTCGATTAAAAATAGAACATAGAACATACACATTACTTAACAAAATT
ACCCTAGAATAAAATTTAATACCTTTATTACCTTATTTTAAAAACTTTTACACCTGTAAACAACATTAATTGTCTTTTT
TACTTACATTTGTCCTAGTTTACATTGAAAATATACCCCTATTGGTAAAAG

> RferU22_CYP4BG7

TCGGCCTGCGGTGCATCATGACGCTTCTCGTTCTCGTCGCCGTCGCAGGTTTTTTATACCTCCTGAACCGGATCCTG
CGTTATTACAAGACGTCCAGGATTCTATCGAAAGTTCCCTCTCCCCCGGGACATTGGCTTATAGGAAACATGGATA
TAATATTAAAAATCAATCCAGATGATTTTTTCGATTTATTGAGGGATTTCAGCAAAAAATATGGATCCATCTACCGC
CTCAGTACCCCATATATTACAACAATCAATATTTCCGATCCTGGCGATCTTGAGATGATACTCTCACAACCCAAGCA
TATGAGCAAGAGTAAAGTGTATGGTTTCCTGCAAAATTGGCTTGGAGAAGGATTATTAACCAGCTCAGGACTGAA
ATGGCAAAAAAGGCGAAAAATTTTGACCAGGGCATTTCATTTCAACATTCTTCGATATGTTTTTCGAGACGTTTTTA
ATGACGAAACTCGACAAGCTGACCCAACAAATTCGCGATATAAACAACGAGAACACGGTACCGGCTTACATCGAC
GTGGTACCACTAATAAGCCACATGACGTTGAAAGCAATGGGTGAAACCTCCATGGGCATGAAGAACATAACCGAC
ACCGAGGTGGTACTCTACAGGCAATCGATTCATAAAATGGGAAAATTGTTGTTGGAAAGAATCGGCTCCCCCTTG
ACCCGATTGTCCTTTATCTACAGGCTGACGAGGAATGGTCGCGAAGAGAACAGAACCATACACCAGCTGCATCAA
TTCACTGATACCATCATCAAGCAACGAGAGAAGCAGATCCTGAATTTGGACAGACTGGAGCTCGCGTCTTCGTCAT
ATTCCGAAAGGAAATTGATGAGAATGCTGGACCTGCTACTGCTGGCAAAAATCGATGACAACAGCATCGATTACG

AGGGTATTAGAGAGGAGGTGGACACGTTCATGTTCGAGGGCCACGACACCACAGCGGTCGCTTTAAGTTTCCTGT
TGCATTCTCTGGCAGGATGAAGTACGTAGGGAGCTGAGGGAGGTGCTGGGAGACAAGCCCCATCCCACCTACGC
CGATCTTCATCGTCTGGAGTACACCGAGAGAGTAATCAAGGAATCTCTGCGTCTATACCCCAGTGTCCCGTTCATAT
CCCGAATCGCCAGCGAGGATATCCTCACCTCGACGGGATACTTCCTCCCCAAAGGCACCGTCCTCCACATGCACAT
ATTCGACCTGCATCGCAGTTCGGAAATATATCCGGACCCGCTGAAATTCGACCCAGACCGTTTCCTGCTGGATAAC
TGCTCGCAGCGCCATCCGTTCGCCTACCTGCCGTTCAGCGCGGGGCCCCGTAATTGTATAGGTCAGAAGTTCGCGC
TGTTGGAGCTTAAAAGTTGCCTGTGCGGAGTGCTGAGAAAATACAGACTAGAGCACGACGGCAAGAACGAGTTC
AGGTTCGCGGCAGATCTGGTTTTACGGACAAAGGACGCCATCAAGATTAAATTTCTACCGCTGAGTAAGGTTAAA
CTCGAGAGAACAGTACAAAACGAAAACCTAATAGCTACCTAATAAAACTTAATAGAATAATTATTAGAACCACTGG
TTTTTTCGACCTGTGGTAATCGTACATTGCTTTTTATATACGCCCAGCGCCATCTATGAAACGTCCCCTTAACTCCTC
GGTAAACAGGACTCTTGATTTTAGTACCCTTCCTTGAGCTCACCAGTTTCTCATGGAACACAACTGATCAATAACAA
AAATTATATAATATTCAAGATGATGAATGGTGAGTAAATTTAAGAAATATATTATTAAATGTCACCTTAGTTCATTA
CAATTTCG

> RferU27_CYP4LC1

TGAAGATCTATGAGGATGACATTGGAGATTTTTAGTACCTTTGTTATGTTAGTTCGCTAGACTTGCTAGTACGTAAA
ATTGTATCCATGGAAAAGTGTATAGTTACGCCGATTTCTTATATCTATTTCGTTATCAGGAGAATATTTCAAAGCAT
AACAAGATACATCAAGTGTGTTGCACTAGCTTTACGTCTTCCCGGACCTGAAGGCGCGCCCATCGTGGGATTTATA
CCAAAACTTAGGGACAACAAGTTTTTAGAAGATCTGGGTACTAATGCATCCAACTATTTCGGCCCTATATTTCGAAT
ATGGATATTGTTCATGCCTTTCGTTTTTATCTACGAACCGGAGGACCTAAAAGCGGTACTGGCACATGGAAGAACG
TCAGAGAAAAGCTTCTTTTATAGGATCATGCATACGTTCATTGGCGATGGCTTGATTACCAGTAATGGGGAGAAAT
GGAAACGTAATCGGAAATTCATTCAGCCGTACTTCCAAAACAACTTCCTACAAAACTATTACGAAATATTTTCCCGA
TGTTCTCTTAAGTTAGTACAGAATGTAAACAACCGGCGTCAAGTCAAGGTAACGGAACTAATCAATGATTGTGTAC
TGGGTATTCTGCATGAGGCCATCTTAGGGGTTCCATCGGGAGAGACAAAGAACAGTCCATTTAGAAAGGGTGAGC
TGCTACTGCTAAAAAGGATATTGAAGCCATGGTTATTGCTTGATGGCGTGTTTAAATGCTCTGGAATGTCCAAGAG
GGAGAAGAAGCAGCAGTCTGCATTGCATCAGTATGTGAAAAGGTTGCTGGATCAACGGCTGGATGACCGAAAAG
ATAACCAAATCAATTGGAGTACCTGCCTCTTGGATTACTTCATTGATTTGATGGAAACAACTGATTTTACTCAGGAA
GACCTTCTCAACGAATGTGTAACATTTATGCTGGCGGGCCAGGACTCGGTAGGAGCAACGATAGCGTTTTGTTTAT
ATTACTTGGCAAGGAACCAGAGTATCCAGGAAAAGTTATTCCAGGAGATCGATATAGTAACGCGAAGTAATGATT
CAGGAAAGATTGACATGGATCAGGTGATGAGGATGCCTTACTTGGAGCAGTGTATCAAAGAGACCCTTCGGCTGG
CGCCCAGTGTTCCTGTGATCTCCAGGGTATTGACAAAAGACGTGGGTTTAGCTAACTACGTTTTACCAGCCGGTTT
GAATATATTCATATCACCATTTATGACTCACAGATTGCCACACATCTTCCCGAATCCTGTAGTTTTTGATCCAGATCG
GTTTTCCGAAGAAGGATTGAAGAACATTCCTCCATACGCTTTCATTCCTTTCAGTGCTGGTAGTAGAAATTGTATAG
GGTATAAATTCGCTTATCTCGAAATAAAAAATATTATCGCGATGTTGGTAAAAAACTATCGCTTAACGCTGATATCT
GGTAAGGATAACCTATCGTTTAGTTACAGAGTCACCCTGAGAGCAAATTCAGGTGTAATGTTGGAGTTCGAACGT
AGAGCTTAAGTATTCGGAATGTTTCATATAATGGTTTAAATTTTTCCAATAATACGGGTGTTCAATATGCGTATAGA
GATGAATAAACTTTATGGAATTAGGAAAAAAAGAAAAAACAAAAGTACTCTGCGTTGATAACCACTGCTTCGAGA
TAGACTTAGA

> RferU30_CYP4KX2

GGAGATTTGTTTATTATTGGTCATTTATTGGAAGTTACATGTTCTCTAGAACAAATGTTTTTGTTGTTAAGAAGATG
GAGTAAAACTGAACACATGAAACCAATGTTTATTGGTAGGATTGGACCTTATTTGTTTCTAAATACTGCAGATCCG
GATATCATTGAGGATATTATATCAGCATCCAAACATCTGGAAAAATCTACTTTATACAGATTTCTTGACCTTTGGTT
AGGAAAAGGATTACTAACGAGCACAGGAACCCTGTGGCATACAAGGAGGAAAATACTTACGTCTGCATTTCATTT

TTCGATTTTGCAAAATTTCATAAGTATATTTAACGAAGGAGGTGATAAATTGGTTGAAACTATAAAACAGGACGTC
GAAAGATTCTGTTGATGTTGTACCGTTAGTGTCTGATGCCACTCTACATGGTATAAATAGTACATCGATGGGGGCT
AAAATGGAATCCATCGAAGGTGGAATTGAGTACAAGAATAATGTGGGAAAAATTACGTATTATTCTGCCTGGAGG
TGTGTAAGACCTTGGTTATTTAACAACATATTTTATTACTACTTTACGTCTAACGGACGTAAAGAAAGACAACTCCT
GCGTTCGTCACACGCTTTCATCGATGACTTGATCAAACGGAGGGCTGCTGGTTTCCAAGTTTTTGACGTTGACAAC
GAAATATCATTCGGACAGGGTAGGAAGAAGTTGGCCATGCTAGACTTGCTGTTGAACGAGAAAATTAAGAACAAC
GCCATAGACAACCAGGGTATTAGGGACGAGGTTAACACGTTTGTTTTCGAGGGCCACGACACCACTGCTTCTAACT
TATCTTTTATGTTGATGCTTTTGGCTTGCCATCGTGATATTCAGGATCAAGTGTACGAAGAATTACAACATATTTTG
GGTCCAGAGTTATCCAGAGAGCCTACATATGCCGATTTGCAAGAAATGAATCTTATGGAAAGGTGCATCAAAGAA
AGTTTAAGGTTGTACCCTAGTGTCCCTTTTATTGGACGTATAGCCAGTGAGGATATTGTTACAAAAGCTGGAATCA
TACCGAAAGGATTATCGATTAACATTCACATATATGATGTCCATAGGGATCCTCAATATTGGCCAGACCCAGAGAC
GTTTGATCCATCTCGGTTTTTACCGGAGAATTGTCGGAACAGGCATCCGTATTGGTATCTACGCTTTGTACGTGCG
GGACCAAGAAATTGTATAGGACAACGATTCGCTATCTTAGAAATTAAGTCGATTTTATGTAAAATATTCAAGAACT
TTATTCTGGAACCGATTGATACCCCAGAATCTCTGGTACTAGTTCAAGAGTTAACTTTAAGATCCGTTAATGGTATA
AAAGTTAAATTTGTACCAAGAATAAATAAGAACAAGTAATCCATATTTTATTTATGCATTATTTGCTTTTATATGAAT
TGTTTTATGTACTATGAATGGCAACTTCTTTAATTAATTGTTTCATTGTGAATAATTGTATATTACTGTGATTAATAA
ATAGTACAATCTAAAACCAAAAGAAACAGAA

> RferU35_CYP4BQ9

GTGGGGACATGCTTACTTATAGACCGATTACTTGCATCAAGTTCCAGCTGAAATGTTCTTTAATGTATTTTGTGCTC
TGATCGCGACCATAGTGGTATGGTGGGTGAATATCTGGAGGAAACACAGAGTACATAGATAAAAAACTTCAATGG
ATAGGAAAAGTAGCAGGCATTCCCTTATTGGGAAATACAATGGAATTCAAGGATCCTACAACAATGCTATTAAGG
ATGGAGCAACGTCTTCTGGAGAATAATGGTTTATGTCGTATTCAAATACTCAGTAGGACTATGGTTCTGATTTCCG
ATTACAATTTTGTGGAATGGTTGCTGACGTCAACGAAGATTTTGGACAAAAGTATCGATTATCAATTCTTGTATCGA
TGGCTTGAAGGTGGAATTCTAATTGCCGATGCTCCCGAATGGAGACCAGGCAGAAAAATTTTAACACCGGCCTTCC
ATTTTTCTATCCTGGAAAACTTTGTAGCCAAATTCAGCAAGCCTACTACAATTTTGATTGATATATTAAAGAAGGAG
TCCGACAAAGATTCAGTTAATATCCACCCGATCATAGCTAATTTTACGTTAGATATTATATGTGAAACAGCCATGGG
GGTCGAAATAAACGCTCAAGAAAATCCTCAGTCGAAGTACGTCCGTTCAGTCCACACCATGTGTAAAATTGTCGTT
GAACGAGCTTTTAATCCTTTGAAGACTAACGATTTTCTTTACAATTTTACATTGGATAATATTGCCGAACGAAAAGC
TGTCAAATATTTACATTCAACGGCTGATGCGGTAATCAAGATGAAAAGAGAAGAACAGCARAAGGAAGAAGAAG
AAGAAAATGATGACGTTGGCGGAAAGAAAAGGGTGGCGTTTTTAGATCTTCTCTTGCGCCATAGAGATGAAAATG
GCCAACCATTGTCCGATAAGTGTATACGGAAACAAGTTAATACAATAATGTTTGCTGGCCATGACACAACGGCTTC
AGCATTATGTTTCACTTTGTTCTGTCTATCTAATCATCTGGACGAACAGGAGAAAGCATTGAGGGAAATTAAAGAA
GTTTCTCATGAAGAAGATACATTTACTTACAGGAATTTGCAAGATATGAAGTATTTAGACCTGATAATAAAAGAAG
CCATAAGGTTATATCCGTCCGTGCCATTTTATTCGAGACGACTGACTAAAGATGTTGTTTATGAAGGTTCTAATGTG
CTTGCCAAGGATATAACATTGATTATATGCGCACATGCCATTAACAGAAACCCGAATGTATTCAAAGAACCTGAGA
AGTTTATACCTTCACGATTCCTGGAAACTAATATTAAACCATTTTCCTTTTTACCATTCAGCGCAGGTCCTAGAAACT
GTATAGGTCAAAAATTTGCTATGCTTGAAATGAAATATGTGCTAGCAAAGATACTCTTGAACTTTGAGGTGTTACC
AGCGAACCCGCCACACAATCCGATCATATCCGCTGAGACTGTATTAATATCGAAAAACGGCATCAAACTTCGTTTC
AGGAATAGAAAATAACGTAGATTTGATTGTTACCTTTGTCTTAACAGTACTGATTGTCTTATTTTATTTATGTTTATT
TCAGTATGAGCATTGTGCCAAATAATATTAATTATTAGTTGTATATTTGCAAAATTTGGCTAATGAAACAAAAACAT
GAAAGTGGGCGGTGATAAAAACTGGAACAACCTAGTGTATTTTTTAACTTTGTATTTGTCATCGTAGGATTAGATA
AAAGCCAGTATTTTTTCTCTTTTCTGCTTTTGGTATATAATTGAAGAATTATTTGTTTATAATATATAGTGTTAGGAT
ATAGCAAAATATTTGACTGCATCAGCCAAACAAACTTCCTTCCTAATGCCCCTTCTTAACTTATCCTACGTGGCATTG

AACTTAATATATATGAGTTATAAATGTTATGATATATTGTTAATATAGTGAAATTGTTACAATATGTTTCTGTAATTA
TATTTAATAATACTATATATAACAATATATAAATATTACGAAATTTTATATCAGTATAATTTGTTTTGTTCTGTCGTTA
GACTTGTAAGCATATAACATCCACTCTAAAGATATCAGATGTAATCATTAAGGTGGACTTCAAGTTGACATTTATAG
CCAACGTAAAGGTACAAACTACTCTAGTTATCGCCATATAGCGTTTACCAGCATCACCAATAATAAGAGTAGAATA
AGATTCGTATAGGTGCTTTGATTAGTACCATATATTACTTAAAATACGAGGTATCAACGTAGACAAGTGTTAAAAA
AACGAATTTAACTTATAAATTCGATGATTGCGATATTTCTCTACATAAAATATTTCTTTAATTTGAAAAAATCTACTG
CCGAAGCCCATCAAATATTTTTAGAAAATTATGGTGATGCTGCTCCAACATATCCCAATTCTTAGTTTTTAACGACAT
AAGAATGTTAATAGCACCAAAAATTTCGAAGGTAATGAATTCTACGACTTAGACTTATGGGAGGAACAGGCAGCT
AAAATTTTCAAAGAATGTCCAGACTGTTTTTCATTTGTTGAATCAACTGTTTCTAGATGTTTCTAATATTCAGTTGAT
CAGTGCAATATCACGTGGCTTGGCTGGTACAAAGTATCGAAATGCTGAAGAAGTACTAAAATTGATCGATGATTG
TATAGCTGCCAAACTAACATCCTTCTTTCGAAAAAATATCACCATGTTGCCAGGAAGATGGAAAAAGTTGTGGAAA
ATGATATTACTATATTTTGATTAACATATATTGCGTTAGTTATTATTCTCTTAGCAAAGAACCTTTTTATAGTAAGAA
TTTTAAGATAAGAATATATACTATAGCTAACA

> RferU40_CYP4G186

ATTTCCACGAGTTGATTCTAAGATTGCCAGTGTATAAAACAAACTTGCGGTGGTCTCAGTCGTCAATTGATGTTGCT
CTAGTTGAGTGGTGATTGTCTGGTAGTTTTTTACTCAGAATATACCTATCTCAGACATGTCGGCAACACTGTCAAGT
CCTGACCAGATGGTCCCTACCGGACTCTCTACTCATACTTTTCTCTATTTGCTAGTACCAGCTCTAGCTTTATTCTACA
CATATTGGCGAATAAGCAGAAAACATATGCTGGAACTAGCTGAAAGAATTCCGGGGCCGAAAGGTTGGCCGATC
ATTGGAAATGCTTTGGAATTTGTAGGGACACCAAATGAAATATTTACACGTATATATAGACGATCGTTCGCATATG
GTAAAACAATCAAACTTTGGGCAGGATCCAGATTAATCATCTTCCTCACCGATCCTGATGATGTTGAAATTATATTA
AGCAGCCATGTTCACATTGACAAATCACCAGAATATAGATTCTTCAAACCTTGGCTTGGAGACGGTCTTCTGATCTC
TACTGGCCAGAAATGGAGAGCACACCGAAAACTCATAGCACCTACTTTCCATTGGAAACCGTAAAGAAAATGGAG
AAAGAAATAGGAAAGGAGTTCGACTGTCACGATTACATGTCTGAAGCCACAGTAGAAATTCTTCTAGAAACGGCA
ATGGGTGTCAGCAAGAAAACACAAGGACAAAGCGGTTACGACTATGCAATGGCTGTTATGAAACTGTGCGAGATT
CTTCATCTTAGACATGCCAAAATGTGGTTCAGACCAGACATCATCTTCAACCTTACCAACACCGCCAAATATCAAAA
GAAACTAATCAACGTGATCCACAGTTTGACTCGTAGAGTTATTCAAAAGAAGAAGGCCGATTTTGAGAAAGGTATT
CGTGGTAGTACTGCCGAACTTCCGGAAGAACTGAAAACCACAAAACCTGAAAGGAATGTGTCATCGAAAACAACA
ACCCTCGAAGGCACATCATATGGCCAGTCTACCGGCTTTGAAAGACTTGATGATCGAGGCATCCCAGAACGGCGT
GGTCATCAATGACGAGGAGATCAAAGAACAAGTGGACACTATAATGTTCGAGGGTCACGACACGACGGCTGCTG
GGTCCAGTTTCTTCCTTTGTCAGATGGCTGCCCATCCGGATATCCAAGAACGAGTTGTGCAAGAACTAGACGAGAT
TTTCCAGGGTTCAGACAGACCAGTCACCTTTGCTGACACCTTGGAAATGAAATATTTAGAACGTTGTTTGTTGGAA
ACTCTAAGATTATTCCCACCGGTGCCAATCATTGCTCGACAACTTCAACAAGATGTGAGGCTTGCGTCTGATCCCAA
GCTAGTATTGCCAGCAGGAGCAACCATTGTCGTTGCTCAATTCAAAGTTCACCGCTCACAAGAACAATATGGGCCA
AATGCTGATGTCTTTGATCCTGACAACTTTTTACCAGAAAGAGCTTCTAACAGACATTACTATGCTTTCATTCCCTTC
AGTGCAGGACCAAGGAGCTGCGTTGGACGCAAATATGCAATGCTCAAACTGAAAATCCTCCTCTCCACTATTCTGC
GCAACTACAAAATCAAATCGAACTGGACAGAGGACGATTACAAGTTACAGGGTGATATTATTCTTAAGAGAGCTG
ACGGTTTCAAAATTTCGCTGGAAAGACGCAAACCAATGAGTGCCACCGCCTAACCAAATCTAATTATCAATAATAT
TGATTCTTGTAGAAATGTAAGGTAGGTTAAGTTGCAATGATGTGAGTAGAGCACTATTAAATATGTATTTGTAGTA
TACCTAATAAAGATCGGGTTTTAATATAAAACGGTGTCAAAGAACAAATAAAGTTCCAAAATAAAAAAAGTG

> RferU46_CYP4LE1

ATTTACGGTCCGATATTTCGAGGCACCATTGGTCCTACGCATATAGTTTACTTATCCAAGGCTGAACATTTAAAGGT
AATATTGCGGAGTTACACCAACATTAAGAAGGGAAGATCATACAACGCTCTTAAACCTTGGTTAGGAGATGGATT

GATATCTGGCGGCGGTGAACACTGGAAATTTCATAGAAAAGTGATCAGGCCGGCATTCTATGTCACAGTCTTGGA
TAAATACATGGACGTATTTTCCAATAACTCCAGTGAACTGTTGGATATTTTTGAACAGAAAGTGGGACAAGGATAT
TTCGATGTATTTCCTCTTATGACCCAACTTTCCTTGGAAACCATAGCGGAGACTGCTATGGGTGTTAAACTGAACTT
TTATGAAAATCCCAAGTTGGAGTATCCAAATGCAATTTTGAATTATCTGTATGTTGCTATCAAACGTATGTTCAATC
CACTGACGCGCAACGAATTCTTGTTCAAACTGACGAAAGATGGAAAGTACTGCAAGCAACAACTGGATATCATCA
ACAGCTTCAATCGAAAGATTATCAGAGAACGACGAGAACACTACGAGAATCACAAAGACGAATACAGATTGGATG
AAAACAGTAAGACGAAGATGGCCTTCCTGGACCTGTTGCTGGCCTGTCAGGAAACCAGCTCGTTCACCGATCAAG
AAGTGGAGGAAGAAGTCAATACCTTTATGTTCGGAGGTCAAGACACCACAGCTTTCTCCAATACCTATACTCTGAT
GGCACTAGGAAATAATCTAGATGTACAGAGAAAAGTTCAAGAAGAGATTGACTCGATTTTCCACGGCGACGAGAG
GCCCGTAACCAGCGAAGACTGTAAAAAGATGGAGTATATGGAGAGGGTGATCAAGGAGAGCCTCAGGTGCTACA
CGATCGTTCCGTTCATTAGTCGCGAGCTGGAAGAGGAGGTGGAAATAGACGGTTTTCATCATTCCCAAAAGGCGT
GGGCGTGCTGATGTCGCTGTACGACCTGCACCAGGACCCGGAACAATTTCCCGAACCCGAAAAGTTCGATCCGGA
TCGGTTCCTCCCGGAAAACGTAGCCAAAAGACATCCGTTCGCTTTCGCTCCTTTCAGCGCCGGACCAAGGAACTGC
ATAGGTCAAAATTTTTGCATTCCGCAGCGTAAAGACCACCCTCACTCATATCTTGAGACGGTATAACGTGAAATGT
CTTGAAAAAGCCC

> RferU49_CYP4LD1

GCAGAAGTTGATAAGGATAGTGTGGAGATCTCGAAGCTAGTTTCCGTATATACACTAAATGTGATTTGTGAGGCA
GCTATGGGATTACAAATCACAGATAAAGACGAAACCCATCTAAAATACATTAATTCTATTCGAAATATTTCTAACAT
TATTAGCTGTCGATTTGTTTCGCCTCTAACTAAAGTTTATCCATAACTTACATTGACATATTATATAGGTAAAAAGAA
GGAAAAATTGAAGACATATATTAATAACGAACCAGAAGTCAAAGAAACTCTCACAGAATCTAAAGAAAAACTCGT
TTTTCTTGATCACTTAATAAAAACCACAATTAATGGAAGACCCTTAAGTGACATAGAGCTTAGAGATGAGGTTAAT
ACATTTATGTTTGAGGGGCATGATACAACGTCTTCAGCAATCTCTTTCTGTTTATATAATTTATCGCTTCATTCTAGT
GTACAGGAGAAAGTTTTGCAGGAGCAAAAGTCCCTTTTCGGTGCAGACCTTAAAAAGGCTACACCAACGTATAAC
CAATTAAACGAAATGAAATATTTGGAAATGGTAATTAAAGAAAGCTTACGATTGTTTCCATCTGTTCCATTAGTAGC
ACGACGATTGTCTTCGGATCTTAACTACAATGGAACAGTATATCCCAAAGGATTAAACGTCCTTCTTTATACGTATG
GTATACACAGAAATCCAAAATATTACACTGATCCAGATCGATTTATTCCAGAACGTTTTGAAAGCAATAATGGAAG
GTTGCCATTTTCCTATCTTCCTTTTAGTGCTGGACTTAGAAATTGTATAGGGCAAAGATTTGCCATGTTGGAACTTCT
CTCGGTCATATCAAAAGTGATACGCAATTTTGAATTGAAACCAGCCAATCCATTCCATGAAGTTGAGCTTTCGGCC
GAACTAGTCCTGATATCTGTAAATGGTATAAAAATATCTCTTTCTCGGAGAAAATAAACACTTAATAGCTGTACATT
TGTTAGTTATGTTATGTTGATTTAAGTAGACTAAATTGTTATTAATTAAAATTGATAAAATAAATGATGTTCTTATCA
CTTTGTTTTAACCTTTCGTATTAGATGTGCTGTTTAATTAACTCTGGTGAACACGCTGAGTACTTATGTTAAGT

> RferU53_CYP4BDfra1

GTTTTTACTGCTTCCTTTTCTATTAATTTTCGGTGCTATATTTTTATTACTGATTAAAAATAAGCGAAAATCGAACAA
GTATTTAAAAAATGTCCCGGGCCCAAAACCATTTTTTCCTTTCGGAAATATGTTGGATTTTGCTGGTGGTAGTAAAG
TGTATTTCTGACAAGTAAAGTGTATTTACCGGTGTTGAAAACCTAATCTGGATGAATACGGAGATACAGTACTTAT
TCACGATGGACCATTTAGTCGGTTATTAATCACCGTTGATTATGAATTGACTGAATATTTGTATTCCTCTACGGTAG
AAATCAATAAATCTGATCAATACAAAATTCTGCATGGATGGCTTGGAGATGGAATTCTGACAAGTTCTGGTAATAA
ATGGAGAAGCCGAAGAAAAGCAATTACGACTTCTTTTCACTTTTCAATCCTTCAAGAGTTTATTCACATATTTGAGG
AAGTCGGCATGAAACTAACTAAAAAATTGAAAGAAATACAAAACAAAAAGTGCATTGATATGACTAGCTTGCTAT
CGCTTATACTTTGGACGTATATGCGGTAATATTTAACGAATAAGACTAATTTCGGCCGTTTATGTCAAAACTTGAAA
GATCTATGCGCTATATTAGCAAGAAAATATTTTCACCTCTTGATTCTAAATTATATCCCCTTACCTGGATGCACCACA
AGGAAAAAAGCACTTAAAAGTAATTCATCGATATATCGAAGATGTGATACGACAAAGAATCGAAACAAAGAAGCA

AGAAAGTTTAAATTATCACGAAGATAACACTAACGATAAAAGTCGAAAAAAGCTGGCTTTCTTGGACCTTCTACTA
AG

> RferU58_CYP4BDfra2

TTAATTAAAGAGTTACTAGTAAGAATTAATCATTATAGCGTTATCATTCTTAGTAGTAGGACAAGTTGTATAAAACC
AATACTATCTATATAGATAGTAGTATACATAATCATAGATATTTAAAACTCAAATTAAACATCGAGAACAACATTAC
AGTAATATTGGAATTTGTGCAGACATACCATGATTGTCATTATTTTAGTGTTAAGTGTTATTGTGCTTATATTGTGG
AGTATTTGGAGTAAACATCATATTAGGAAAAAATATTTTCAAAATGTTCCCGGTCCTAAGCCTGTACCGATTTTCGG
GAATACTTTGGATCTTATAGCAGGACCTAAAGTATTTCTAAAARCTATATCTGGTTGGATGGACATATTTGGTGAA
ACAATATTAGTACATGACGGGCCTTTAAGTGTTCTACTTATGACTATGGATTATGATCTTACTCAATTTATATACAAC
TCTTCTGATCACATAGACAAGGGATACCAATACACCTATCTTCATGGTTGGTTAGGCAATGGTCTTCTTACATCGAC
TGGACTTCATTGGAAACTGCATCGAAAAGCGATAACACCATCTT

> RferC402_CYP4DV1

GTGACAAAACAATATTTTCTATCTACAATTGGTTTATCTCCGCACAAAAGGTTAATTTGTCAGTACGAGTCAATATA
TTAACGAGCGCGAAAGTTAAAATTTACTATTAATGACGTTACAACGAGTAGATATAAATTAAATCCTAAAACATTC
GTCGTGCTGTAAACAGTTGCGATCATTCATTCAATATCGCAATAATGCTGCTCGTTTTTCTTGAAGTTATTTTCGCG
GTACTTGTGGTGTATTTTAGTCTCAAATTCTGGAGAGTGTATAGGATTAGAAAGCAACTAGAATGGGTTCCTTCGG
CTAAACGTTTGCCATTTATTGGAGCATTACTTGAAATGGGAAATGACTCAACAGAGTTGATTTATAATTTCACACGC
CTTACTGCAAATCCTAAAAAGAGTTGTTACATCGAGATAGGGTTTGATTGTGAGGTGTTAACTAGAGATCATGATT
TTACTGAGTTCCTTCTCAGTTCGAACAAATTGATCAACAAGTCCTGGGAGTATCAGATGTTCGAGGATTGGTTAGG
TACAGGTCTATTAACAGCTGGAGGTACAAAATGGAAGAAACGAAGGCGTATCATTACACCAGGTTTCCATTTCTCG
ATTTTGAATCAATACATTGAATCGTTTGACTTAAATGGACGTAAGCTAGTGGAAATATTGAAAAACGAAGGCACCA
AACCAGATGGCGTCGATATATATTCATATATAACCAACTGTGCACTGGATATTATTAGCGAAAGTGCTATGGGCGT
GTCGGTAAAGTCACAAGAAGGACAAAATCAACAGTACGTCGTCGCCGTAAAGGATATCCTATCCTCGATTTTGGAT
CGATCATTTAGTCTTATTAAATCGAACCCTTACTTATTTCCTTTTACTCCGGATTATTGGTACCAGAAACGTTTGGTG
AAGATTCTGCATTCCTTCACTAATAACGTCATAGAGAGCCGACTGAAGAGTCTACAAGAAAATGGCGGAGTG

>RferA_c4897_CYP4G15

GTCTTACGCAACGGAGAGCGTGGTGCAGTCCACAACATGGGCGGCCACGAGTTTGTTCTACATGCTGTTGGTACC
AGCTCTGGTGTTGTGGTATGCCTACTGGAGGATGTCACGGCGGCACATGTATGAACTAGCTGAGAAGCTGGAGG
GGCCCGCTGGCTTGCCGCTCTTGGGAAATGCCTTGGAATTCACTGGCGGTTCTGCTGATATATTCAAGAACTTGAT
AACCAAGAGTGCGCCGTACGACAAAGAGGCAGTGGTGAAGATCTGGATCGGGCCTCGGCTGCTGGTGTTTCTGT
ACGAGCCTCGCGACGTGGAGCTCATCCTCAGCAGCCATGTCCACATTGACAAGGCGGACGAGTACCGGTTCTTCA
AGCCTTGGCTTGGCAATGGACTCCTCATCAGCACAGGTCAGAAATGGCGGTCTCATCGCAAACTGATCGCGCCGA
CATTCCACTTGAACGTTCTCAAGAGTTTCATCGATCTATTCAACGCCAACTCGAGAGCTGTTGTGGACAAAATGAA
GAAAGAAGCCGGAGTATTCGATTGCCATGATTACATGAGCGAGTGCACGGTCGAGATATTGTTAGAGACCGCCAT
GGGTGTCAGCAAGAGCACTCAAGACCAAAGTGGATTCGAATACGCGATGGCTGTTATGAAGATGTGTGACATTCT
GCACTTGAGACATACCAAGATCTGGCTGAGACCAGAAATGATATTCAATTTCACTAAATACGCAAAAAGTCAGACG
AAACTGCTCGATATCATTCACGGGTTGACTAAAAAGGTCATTAAGAGGAAGAAGGAGGAATTCCAATCGGGCAAA
AAGCCTTCCATATTAAATGAAACTACAGAAGTGAGCACCAATGAGCCTACAGCCAAAACAACGTCCGTGGAAGGT
TTGTCATTTGGACAATCAGCTGGTCTTAAAGACGACTTGGACGTAGATGATAACGATGTTGGAGAAAAGAAACGT
CTGGCCTTCCTGGACCTTCTTCTGGAAAGTTCTCAGAGCGGTGTTGTGATTTCTGATGAAGAAATTAAAGAACAAG
TTGACACTATTATGTTCGAGGGTCATGACACAACAGCAGCTGGAAGCAGTTTCTTCCTCTCTATGATGGGGATCCA

TCAAGATATCCAAGACAAAGTGATTGAAGAATTAGACCAGATTTTCGGTGATTCTGACCGTCCAGCCACATTCCAA
GACACGCTAGAAATGAAGTATCTCGAGAGATGCCTGATGGAGACGCTTAGGATGTACCCTCCAGTGCCTATTATT
GCTCGACATCTTAAACAAGATGTTGTTCTTCCATCCAACGGAAAGAAAGTACCAGCTGGTACCACGATAATTATCG
CAACGGTAAAACTCCACCGTCGGCCGGACATTTACCCCAATCCCGACAAATTTGACCCCGACAACTTCCTGCCAGA
GCGCTCAGCCAACCGCCACTACTATGCCTTCGTACCCTTCTCTGCTGGTCCCAGGAGCTGTGTGGGACGTAAATAC
GCCATGTTGAAACTGAAGATCATCCTGTCCACTATCCTGAGGAACTTCCGGGTACACTCCGACCTCAAGGAAGAAG
ACTTCAAACTTCAAGCAGACATTATCCTCAAGCGAGCCGAAGGGTTCAAAGTTCGGTTGACGCCCCGCAAGAGGA
CGACCAAAGCTTGCTAAACAGTTTTTAGGAAGCGCACTGATTATGAGCTTTAAGTGTTGACGGAAACTCTGTATTT
TCATGAAATTCTTCGAAAATTTTAGCACTTTATAGCATAGATTTATGAACACTGTATTTAAAAGTAACTATTTGATAT
ATAGTTT

> RferC9515_CYP4BD12

GTACATGTTGTTGTTTCTTCGCCTCGCTAAAATATTCAGATTAATTTTGAATGAACACTGATTTCAAACGATGTCGCT
AGTGTTAATGTTTGGTTTTTCCATTGTCGTCTCATTGTTGGTAATATTATTGATAATTAGGCGACCGAAACGTACCA
AAAAGTACTTGGAAAATGTACCAGTACCAAAAACAACGTTTATATTTGGAAATGCGTTAGATTTTATGAAAAATCA
AGAATATTTGTCTGTCCTAAAAAAGTATTTGGATGATTATGGAGATACTGTTGTAATCAGAATTGGTCCTTTCAGCA
AGTTACTGATTACTGTTGATTATGAATTTTCTGAATTTTTATACAGCTCAACAGTCGAAATAAATAAATCTGACCAAT
ACAAAATAATGTACGATTGGATTGGAAAAGGGCTGCTAACAAGTGCAGGATACCAATGGAGAGCTCATAGAAAA
GTGATAACACCATCTTTCCATTTTTCGATCTTGCAAGATTTCGTGGAAATATTCGAACAAGTGGGCAATAGATTAGT
CGATAAGCTAATGGGCACAGAAGGCCAAAAAAGTGTAGAGGTCTTTAATATAGTATCCCAATATGCACTTGATGT
GATATCTGAAGCTGCAATGGGAGTAAAATTAAATGCGTTGGAAGATGATACATCTATATATGTGAAGAATGCCAA
GGATCTTTGCAAGATTTTAATCAAGAAAGCATTTTCGCCTATCAATGAGAAGCTATACCCACTTACTTTCATGCACT
ATAAAGAACAAAAACATTTAGATGTTATCCATCGTTATGTCGAGAATGTTATACAGCAAAGAATCCAAGAAAGGG
AAGCTAAACTAATAGTTAGTGATGAAACTAAAAGTAACACTGGAAAAAGAAAAAGATTGGCATTCTTGGACCTAT
TGTTAGAAGCTAGAATCGATGGGGAACCTTTATCTTTAAGGGAGCTTCGTGATGAAGTTAATACATTTATGTTCGA
AGGTCACGATACAACTGCCACTACAATATCATTTTGTCTTTATTTGCTGGCAAAACATCCATCAGTTCAAGAAAAAG
TACTAGAGGAACAGTATGCCTTATTAGGAGATGATTTGAAGGGTGTCAAAGCCACATATGCCAACCTCAATGAAAT
GAAATACTTGGAGATGGTTATTAAGGAAACACTGCGATTATACCCTCCCATTCCTTTTGTTGGACGAAAGTTAACA
AACGATGTGGAATTTAATGGGACGATATATCCAAAGGATATAAATATTTTGTTGTTCACGTATTATGTCCACCGAC
AGGCCAAATATTTTCAGGATCCTGAAAAATTTAATCCAGAACGTTTTGCATATTCTGAAAGGAAGATTCCTTTTGCT
TATATTCCCTTTGCAGCAGGTTCTAGAAATTGTATAGGACAAAAATTCGCTATGTTAGAGATAACATCGGCTCTCTC
GAAAATCGTTAGGAACTTTGAACTGATTCCTGCCATTCCCAAACATGATCTTGAAATCACTACTGAACTTGTTCTAG
TTTCGAAAAATGGCATGAGAATTGCACTTAAAAAGAGATTTTAATGTATAGCATAGATCGGAAGAGCGT

> RferC18014_CYP341Sfra1

TAGTGCTATACAAATTTTGAATTAAATAGTATATTGGATACGTCATATTAATATAGGGATTTTTCGAATATGTTGTG
GCAAATATGTTTGATTTTGATCGGGATATACGCGATGTTTCGTTGGAGAAATAGAGAATTTAATAAGATTGCAGAA
TTACTTCCAAATAAAATGAAACGGGTGCCGATTTTGGGACATGCTGTACATTTCCTGGGAAATTCTAAAGAACGTA
TGCAAGTAATGAAGGCAATGGCAGAAAATGCGATGAACAATAAAGAAGGGATGACTAGTGTATGGCTCGGACAT
AAATTTTTTGTTATAATTGCAGATCCGGAAATTGCTGAAGTGGTTTTAAAAATGTGTTTAGAAAAAGATAACATGA
TGAGATTTACAAGTGTGCTAATCGGTAATGGCAGCATTTTCGCACCAGTTAAAATTTGGCGTCCTAGACGGAAAAT
TTTAGCTCCAACCTTTGGAACGAAAACCCTA

> RferC26591_CYP433A3

ATCAAACTACCTGCAATGCTAAACTTCAGCATTTTTATACACGGTTTCCATCACAATCCGCAATATTACCCAAACCCA
GAAATATTCGATCCTGAACGATTCGCCAATTCGAAAAATATTGATAAAATGACCTACTTACCATTTGGAGTTCAACC
CAGACAGTGTTTGGGAAAGAACTTTTCTATGTTAGAAATGAAATGTGCCTTATCGAAAATAATACGAACTTACGAG
ATTATAAACATCGCAGAACATCAATTGGAGATCAAACCAAAACTTGTGTTATTTTCTATATCTGGTATATGGGTGAA
GATTCGAAAACGGTATAAGAAAGTATCAAAATTAGGTTTAAATAAAACTACAGAG

> RferC32960_CYP341Sfra2

TAAAGGAGTCACTGAGATTATATCCGCCAGTGCCAGTTGTTGTCAGAGCGGTGGACAAAAACATTACTTTACCGTC
AGGAATAACTCTGACTAAAGGCGTGTGCGCCCTGATCCATATTTGGGCAATTCATAAGAATCCAAAGTATTGGGGT
CCAGATGTAACTCGGTTTAGACCAGAAAGGTTTCTCGAGGGCCCATTGAAACACCCGTGTGCCTTTGTGCCTTTCA
GCAATGGATCTAGAATATGTTTAGGTTATCAATACGCCATGATGTCAATTAAAACAGTCATGGCACACCTGATACG
ACGGTATAGAATTTTACCAGCAAAAGTACCGAACGACGTCAGAAAAGCGATGGAATCTGATGATGATGACGCTCT
ACCTTTAACCTATGAACTTATGATAAAGCATGCAGGCAATTTTGAATTACAATTAGAATTGAGGACTTAATAGGTCT
TCGAAAGGTTTTTATAAACCACCCAATCGGCT

> RferC35523_CYP433A3

GGTAGTTTGATATTACAATATTGTAGATCTTTGGTGATTCTTTTTCCAACTGAAGGAACAATGCTGTATAGTCGTAA
AGTTTCCTTAATCACCATATCTAGATAAACCATTTGTTGAAGGACTTCATAATCAATTTGTGGACTTTCCTTTTTAAA
AAGTTGCTGTTGCTCATTTAAAAGCTTTTTCTGTATGTCACTGTTTTTACCAATTTCATAAAGGGCCAAACATATTGC
GGTTGAACTAGT

> RferU3_CYP412B1

CATAGGTTTGCAGTTAGTGTGTTAACTAACGTTCGGTACTCTTACTGCTGTTGTTCAAGTATTTTACAAATAAAAAG
AAACATGATGAACTGGAAAAATATAGCTTTTTACACTGTACAATTTGAAATAACAATCTTACTGTCCGTAGTAATAT
TCTTTGTAGCGCTATATGGAAATNNNNNNNNNNNNNNNNNNNNNNNACCTTTTGTTGGAGCTGCATACAATTT
TATGGTTAGACCTGAATACATTTACAGTATATTCACAAAATTACAAGAAAAATATCCTGATGGAGGAAAATTTTGG
TTTGGATCGAAACTAGTCTATTTTCTACATAAACCGGAACATATAGGGAAGATTCTTGGAAGTCACAAATTAGTAA
AAAAGGATGAACTCTATAGGCCAATAATTGAATGTATAGGTGAAGGTCTTTTAATAGCACCAGTCGAAAAATGGC
GCAAACATCGCAAAATGATTATGCCATCATTCCATCAAAAAGTTTTGGATCAGTTTGTTGTAGTATTTGCTGAAAAA
TCGGACATTTTATACAACATCTTGAATAAACATGTTGGGATAGAAATAAATATGTATGATTTGCTTACAAAATGCAC
ATTAGATACAGTATGTGAAACCGCAATGAGATTACAACTAAACATTCAAATAGAAAACAATAACTACAGTTACATA
TTTGACAAAATAATGTTGCTGACTTATCTGAGGATTTTTCAAATATGGAACCACATTTATTGGATATGGAATTTTTT
GGGCTATGAGAAAAAATTACAATCGTATCTGAAAGTATTTAATAATTTATCGAATAAAGTTATTCAAGAAAAGCTA
GAAGAAAGACAAAACAATGTTGAGAATAAAAATGAGGTAATACTCAATGGAGGAACTAAGAAGCGTATGGCATT
TTTGGACTATATAATGGAAAATGAAAGCTTTACAGCCACCGAACTTAGAGATGAAGTCAATATGTTTCTTTTTGCA
GGAACCGATACCACATCTTCTACGCTGTCTTTTGTATTTGCTATGCTAGGAATGTTTCAAGATGTTCAGGACAAAGT
TTTAAAAGAAGTATTAGATGTTATTGGACCTGACAGATATGCAGAACCTCAAGATCTTCCAAATCTACAATATACA
GAGAGGGTAATAAAGGAAACATTGAGGTTATTTCCTGTGGGAGCTTTTTTTGTTAGATCAGTAACGGAAGATACG
GATGTTGGTGACTTCGTAATACCAAAATTCTCCACTGTTTATTTTGGTGTAGTTTATATACACAGAAATGCTAAATA
CTGGCCTAATCCATTGAACTTTGATCCAGACCGATTTTTACCCGAAGAAGTAGCAAAAAGACATCCGTGCTGCTAT
ATCCCATTTTCCTATGGGCCTAGAAATTGTATAGGAAAGAATTATGCCATGATGAATATGAAAGTTCTTGTATCTAC
AGTTTTAAGAAAGTTCAAAATACATTGCGCATACAAACGAATAGAAGATATTAAATTGAAAACCAATATAGTGCTG
AGATTCAGAGATGGTGCTAAAGTATCAGTTACACCACGTATTAAATAGAGCTAAAATATTCAATCAAATTTTAAAT

AAGGCGTATATGTAATAATAAATATAAATAAAACAAGAATAGTAACTGTAAATAAATAACACGTGTTATACAAGAC
TTATAAAGGACTAAATATTTTTACATTAAAACTAATATAGCATTTTAAAAAAA

>RferU8_CYP349

GTGGCAATTAAGCCAGCAAATGGAAGAGGAAAAGAAAAATAAGGATATATATTATGAAGCAGCAGAAGGGCGTA
AGCTTCTAACGTTTATTGACATGCTTCTGAAGGTTACTTATGAAGATAACAAAAGTTACTTTACTGATGAAGAAATA
GTAGATGAAACAATTACTTTAATGGGAGCGGGCTCGGACACTACCTCTAACACTAATGCATTTGCATTATCTTGTTA
GGCATGCATCAAGATATACCGGAAAAAGTATTAGCTGAAATCTTAGAAGTGGTAGGCCTGAGAAGAGATGTATGT
CTGTGGATGATTTGGCACATTTTAAAATTTTTAGAAAGGGTYATTAAAGAAACACTTAGAGTATTTCCAGTTGGTCC
CATTTTGGCTAGGACTTTGGACGGTGACATTAATGTTGGCGATCATGTACTTTCGAAAGGCGGATCGATCTGCTTG
GGAGTACTAAGATTACACAGGAGTGAGAAATATTGGCCTAACGCCTTGAAATTTGATCCCGATCGTTTTCTTCCAG
AAAATGTTGCAAAAATCCAACCAGGCTCATATGTACCGTTTTCTTACGGCCCCAGAAATTGTTTAGGGCTCAGATA
CGCAATGATGACTATGAAAGTTATATTAGCAACAATCTTACGCAGATATAAAGTATTTACTGATTATAAATCTATAG
AGGAGATTGATATAAAATGCAGTGTTATGACCCGCCCAATTAATGGTTATAAATTATATTACGAATCAAGAAAATA
GTAATAGCCTCCTTCGAGATGGTGTTATTTTGGGATGGTCCTTAAAATGTTTGATGGAAATAGTAAATACATAATA
ATTAGCGCCTAGTTTTAAAAATTTTGTTAATAATTAATACAATGTATCTTTTTACAAAAGAAACAGAACAAAAGACA
AAACATGTCGGCCGCCTCGGTCTCTAGAAT

> RferC23468_CYP15A1fra

ATTGTCTTTGTTGAGGGTAATCAATGGAAAAATCAACGAAAGTTTTCTCTACATAGTCTCAGGCAATTCGGTTTGG
GACGTAAAATCATGGAGGAACAAATTCGAGAGGAATGCATGTTATTGATCGAGAATTTCAGCAGTCAATGTAAGA
AACCGATATTGATGGATGACGCCTTTGGAATTCCCATTTTGAATGTTTTGTGGATAATGATTGCAGGGAAACGCTT
TGATACTGATGATAAAACG

> RferC30851_CYPfra

GACTTTTCAGTCAAAAATCAATACCAAAATGGCGACGAAATAGAAAATTTATTACACCAGCCTTTAATTCACAAATG
TTAAATAGTTTTATGGAAGTATTTGTTCAACGATCTAATGAACTAATTGAATTTATGAGACAGTATGAAAAGAAAA
AAGATGTTGATTTATATGATTTCCTTTCCCGATATGCATTAAAAACAATTTGCGACACTGCAATGGGAGTTAACATA
GACACAGAACCAGAAGGGGACAGTTTTAACCATTGGATAGAGAGAGCCCTGGAAATATTAATAGAAAGGATATTT
AATATATATTATCAAATTGATATTATTTTCTGCAATTCAGCATTAGGCAAAG

>RferC4152_CYP410

ATTATCTAAAATGTATACCGTCCTGACCATTTTACTCTGTGCCGTGTTATATTGTTTAATTTATATTTTATTAAAATTA
TATGCCAAATGGGATATTATCAAATTCTATATGAAACATAATGGTCCCAGATATTTTCCATATTTCGGAAATATCGT
TTTTTCTGGAAATAATGTACTGGCAACATATTTGAAAGTCAGACGTGATTATGGAATGCCTTACGCTATCTGGATTC
ATGATGATCTTTGTTATGCTACAGATGATCCTAAAGAAATTAAAGTGATTTTAAACCATCCAGATTGTTTCAACAAA
GCGACATTTTACAGTAAAGTAGTTGAATCATGTCTTTTCGATGGGTTTGCTTTAATACCTATATCAGAAATTGATAA
ATGGAAGTTATACAGAAGATTTGCGGCGAAAAGCATGCGACCGGCAATGTGGAAAGACTTTTTGCCAGTTTTCCA
TGAATACAGTCAGCGTCTAAACCGGTCCTTCAAAAAATCCAAGCAATTTACCAATTTCGACGATTTTTTCGCTAGCT
TAAACAACATCGTTTTCCAAACCTTTATGGTAAACTTTTTGGGAATGGAAAATTACCATATGAGAGACATCGATAAC
TTCACTACAGGAATTCAAAAACTTTTAGCAGTTCTCGGACGAAGAACCACTTTTCAACGTGTTATTCCCGATTATTTC
TGGAAATATCATACAGAAAATGGCAAGGATTTCTGCAATATAATCAATACCATTCGAAACGAACAATGATACTTTG
AATAGTACCAAATTTCTAAATAGCTATCTACAAGACGAAGGTACCGTATTTACAGACGAAGAGATATTCCACCAAG
TATATTTCCTGGCAGCAGCAAGTACTGAAACATCAGCTTTAAGCGTCTGTTTTATTTTGTTGCAGCTTGGTATGGAT

CAAGACGTACAGGAGAAAGTACATCAAGAAATAATTGATTTACTTGGTACGGATAAAGAAATTGCTCTTCAGGAC
TTGAACAAACTTAAATATACTGAACAAGTAATATTTGAAACTCTAAGAATCTATCCAGTATTGCCTTTCTTAGGACG
CCAAGCTACATCAAATATACGTTTAGATGGAAAGATTATTCCCAATGGTACTAATATCATTGTTTCACCATATATGA
TACATAGAAATGAAAAATATTGGCCTGATTGTGAGAAATTTAATCCTGAACGATTTAATGCAGAAGAATCAAAAAA
TATTGAACCTGGCTCATTTGTACCATTTCTCTTGGGACCTAGGGATTGTGTCGGTCGCCACTATGGCATGACGATGA
TGAAGGTATTTTTAACAAACATAATAAGAGAATTTAGAATTGTTGCGAGAAAACAAAGCATCGATGATATGAAACT
GAGTTACTACATTACGATGAAGCCAACCAGCAATGTCGATTTACAATTCATACAAAGAGCTCCAAATCATTAATATT
TTGTTTCTATCAATAAATTAAACATATGAGACTATTTTTACTAAAATGAAACATGTATAACATTATTGAAGAATGCAT
TTCAACACTTTATTATGTACATAATACTATAATGTTTATTTGTTCAGCGCTAGACCTATTTTCTGTCATCCACTTGATG
GTGTTTGGTCCTGACTAGTTGGATTCTGGACTTTTAAAATTTACTAGGTCGTGCCAATTCACAATTATTTTTATCAAT
TTGAAAATTTACCAAATTTCCAACTATACTGAATGACATTCTGTAACATTTTGCTATCAAATAAAGACGTAAAGTAA
TCTGGAAAATAATATATGACTTCGTAAAAATAGCATATTGCATAGACCGATAACTTCAGGCTCCGCCTATCCCAAAA
ATGATCCTTCCTAAATTTCTTAACTTATACAACCTACATTATATCCGAACAGGTTTCCTTATATATTAAATTAATCACG
TCAAATAGATAATCACATCAAATAGAGGAATAAAAATATAAAAAACAAAAAATGAGTAATAAAATCCACTACTCAC
CTACTATAAAAAGAGAAACAGAACAAGATATGACGGAAAGGGTTTTCCTAGCAGTCCTCTAATATCCTCAGTGAAC
AATATACCGGGTTGGTAAAGTATTAAAGAATTCAAAATTATCTTCAAACCATCCAGGTAGATGCGAAAGTTAAATT
CAGAAACCAATAGTTCAGATATAAACGACCGCATAAGTCTCAAACTAAAAAATTGTTGTTTGCATTTCAATAAATG
GAATAAAATATAAGTATATGCTTTATAAGAGAGCTTTGGTATGAATATATAATAGTTACAGTATATCGAAGAAGAG
ATTCTGGACGCAAT

>RferU4_CYP306a1

CAAAGAAGAAAATTACCACCAGGGCCCTGGGGTCTTCCGCTAGTCGGATATCTACCGTTTCTCGATCCCAAGGCTC
CCCAAGTAAGCCTCACCGAACTCGCTAAAAAATATGGTCCCATGTACAGCTTATTGCTTGGTAACGTGCGTACCAT
CGTACTCAGCGACCCTAAATTAATCAAAACCTTATTCGCTAAAGACGTAACGACAGGAAGAGCACCGCTGTATCTC
ACTCATGGCATAATGAATGGTTACGGTATAATTTGCGCCCAAGGCGATTTGTGGAAAGACCAAAGGAGATTTGTC
CATAATTGCCTTAGACATTTCGGCGCAGAGTACGCGGGTTGGGCCTAGAGATAATATGGAGTAAGCTCATTATTAA
ACACGTTAAGGAATTTGTGCGGTATATCAAGACATTAGATACAAAACAGCCTATAGATCCTTTGAGACCACTAAGA
CACTGCTTGGGATCAATTATAAGCGAAATTGTGTTTGGAAGGAGCTGGGAACGTGGTAACGAGACATGGGAGGA
GCTTCAAGAACTTCAAGAGGAAGGATTAAAATATATTGGTATAGCTGGTCCATTGAATTTCTTACCATTCTTGAGGT
TTTTGCCGATGTTTAAGCGACCAATGTCTTTCCTGATCTAAGGCCAACATAAAACGCACCGATTTTACCAAAAACTG
ATCAACGAAGAAAATGTATTGTTAAATAAAAGTAGATCCGCAGACCTTGACACCAATAGCTACACCAACTTGATAC
AAGGTTTTCTTATTGAGAGAGAAAAAAGACAAAATACTGACGACGTGCATAAATATTACAACGAAAAGCAATTCC
ACCATTTACTGGCCGACGTATTCGGCGCTGGATTGGATACTACTCTGGTCACCCTCAGGTGGTTTTTATTATACATA
GCAAGGAATGCAAAAATACAATCTCAGTTAAGAGATGACTTAAAGACTATTTTAAACGACCGTGATCCAGCCATTA
CGGCCGGGGAATCCCTTCCATTTGTGGAAGCCTGTCTATCGGAAACTCAAAGAATTCGATCAGTTGTTCCAATTGG
TATTCCACATGGCGCTTTGGATGATATTAAGCTAAATGGTTACGTTATACCAAAGGGTGCCATGATTGTTCCTCTAC
AGTGGGCCGTACACATGAACGAAAAAGTATATCCTGAGCCTGAAAATTTTAATCCAAGTCGTTTTATCAATGAAGA
AGGACGATATTTCAAACCAGAATATTTTATTCCATTTCAGAATGGAAAAAGAATGTGCATTGGCGACGAGTTAGCC
AGAATGATGATGTTTTTGGTATCTACTACTATTCTGAAGACGTTTCATTTAGATCTTGAAGATCACAAGACGTGTAT
GGAAGGTGAAAGTGGAATCACTCTATCACCACAACCATACAAATTAATATTTTCAGAAATATAATATTTTTTATAAG
AATATACCTTCACAATTATATTAAAATAAATTGTTTCAACGTTTAAACAAGAAAAAAA

> RferU23_CYP305F1

GCCATCGTATCGCGCTGACTTACGGACGGAAAAATCGTACAATGCTTACGATAATACTATCTATTGTTCTCGTAGTT
TTGTTGCACGTGTGGTGTTTTTTCAGTGAATGCAAGAAACCAACAAATTATCCTCCAGGACCTCCGTGGTTACCTTT
CCTCGGCAGTCTATTTCAGTTGAGGTCGTTAAGTAAAAAAGCGGGCGGGCAGCACGTGGCTTTGATGCAACTGAC
GAAAAAATACGCCTCGGATGTGATAGGATTGAAATTAGGGTCGGAGTACGTAATAGCCGTATGTTCGTACGCCTC
CGTGCGGAAGATCTTAACGTCGGAGGAATATGAAGGACGTCCCGACAATTTCTTTTTCCGATTAAGATCGATGGGT
ACCCGGAAAGGTATAACGGGAACAGATGGTGAATTATGGAAAGAACAACGAGCTTTTCTAGTCTCTCACCTCAAA
CAGTTGGGCTTTGGAAAGGACAATGTAGAACAGATGGTTAAAGATGAAATCAGAGATATTATAACAAGAATGGA
ACATAACTTAAACAATGTCAATATTGCAGAGGTTCTAGCACCATCAGTATTAAATATTTTATGGTATTTAACAAGTG
GCAAAAACTTAAGAGGTAATCAGCGTGTGACTAATTTGCTAGAACTCTTGCACAGAAGATCTAAAGCATTTGACAT
GTCTGGAGGTATGCTCAATTTATATCCATGGCTAAGGTTTCTTGCCCCAGATAAAACGGGGTTCAGCTTGTTGCAA
AAGATCAATTCAGGTATTAGCGACTTTATTAGCGAAGCTATTGAGGAACATAAGAAGTCCTTGATTGATGAGGATA
ATAGTGATGTAATTTATGCATATTTAAATCAGATGAACAAAGGACCGAATGGAAAAAAGTATTTTACAGATGATCA
GCTGGTAATGGTTTGTTTAGACATATTCTTGGCTGGATCAACTACTACCACTAACACAATCAATTTTGCATTGTTTGC
TATGACCCAATATCCTGATATACAAGAAAAAGTTTATAAGTGTATTGAAAACCAATTTGAACCTCAATGTGAAATCT
CATATAGTGATAAACAACGAGTTCCATACATTGAAGCAGTATTATTAGAATGCCAAAGATTTTTTCCTGTGGTGCCA
GTGATTGGACCAAGAAGAGTCTTGAAAACATGCCAACTGGATAACTATCAGATCCCAAAGGATTCCACTATATTAA
TAAATATCTATGCTGTTCATCACGATCAAGAATATTGGCAAGATCCCGAAGAGTTTAGACCTGAAAGATTTTTGGA
TAACCAAAATAAACTGATTGGCCACGAAAGAGTTCTAACTTTTGGATTAGGTAAAAGAAGATGTTTAGGCGATGCC
CTAGCGAAAACGTGTGTTTTTTTGTTCTTTGTGACGATTCTGAGAAAGTTTAAATTGGAAGCTATAACTAAACAGA
GCGAATTAGATGACAACATAGCAGGGATCAACATGAGTCCACAAAAATATAAAGTCAAACTTACACCAAGAACAG
TTAATCAGTAGGATTAATAGCAAGATTTTTTACAATTATGTTATAATCAGTATGAATAATGATATTAATATTTATTTT
TAATAACAATTGTATAATATTTTATAAAAGTTTTAACTTATTATAAGAGCAATATTATTTTCGTGATAGTATTGTGTA
ACTCACTGTTTAGTATTAGTAGTAAAAAGTTATTAGCATTAAATAAATAACATAGTTTTGTAATGTCTCCCTCACTTT
GTAAATATTTATCTATTATAACTTTTATATTTCGGAATTGTTTTTATCTAACTT

> RferU36_CYP18A1

CTCAAGAACACTGTTTAAAAAATAGCACGGTTGCACTCGCAAAAAGAAACAAAAAAAGAGTGAGAAAGACAGAC
AAACAAATAAATATGTTCGTGTATAGTGCCTCGATACTGTGGAACGCGTTAAATGAAGACGCGACCAGGAAGATC
CTGCTAGTGTTCGTCGTCGTGTTACTGATCGTCCGATCGCTTCAATCCTGGCTGGAATGCAGGAGTTTGCCGCCGG
GACCGTGGGGATTGCCGGTTTTCGGTTCTCTGTTGAAAATTAAAGGTGATCTTCATCTTTTCTACCGCGACCTGACG
CATAAATACGGTTCGTTGATATCCGCGAGACTTGGCTCGCAGTTGATTGTTGTTTTGAGTGATTACAAAATGATCC
GTGACGCGTTCAGGAAGGAGGAATTCACGGGCAGACCCAATACCGAGTTCACCAACATCTTAGAAGGTTACGGCG
TGATCAATACCCAAGGAAAGCTGTGGAAAGACCAACGACGGTTCCTTCACGATGGTTTACGCCATTTCGGTATGTC
CTACTTGGGATCGCGCAAAGCCCAAATGGNAAAAAGAATTGTTAGCGAAGTCGACGATTTCCTTACTATTCTTAAA
GCAAGCAAGGGTCAGCCGATAGATCTCAATTCCGTCCTGGCTGTATCCATTTCCAATGTTATATGCGACGTGCTGA
TGTCGGTAAAGTTCTCCCATAACGACGCCAGATTCATCAGGTTCATGGAGCTCATTGAAGAGGGCTTCAGACTATT
CGGTTCCTTGGAGTACGTGNTTTTTATCCCGATCCTGAAATACTGGCCCGGTCACAGTTACACCCGTCAAAAAATC
GCCAAGAACCGTGAAGAGATGGCTCAGTTTTTCCAAGAAACAATTGACGAACACCGAAGGACATTCGATCCAAGT
CATCTTCGCGATCTTTTGGATACCTATCTGTACGAAATCCAAAAAGCAAATGAGGATGGAGTTGGACATAATTTGT
TCGAAGGAAAAGATCATGATCGTCAAATGCAACAGATCATGGGTGATCTCTTCTCTGCTGGCATGGAAACCATTAA
GAGTTCCTTACAATGGGCTGTCTTATTTATGTTGCACAATCCTGAGCATATGAAGGCCGTTCAAGAAGAACTGGAC
CAAGTCGTCGGTCGTCATAGGCTACCAAAGTTAGAAGATTTAGCTTATTTACCAGTGACGGAATCCACGATCCTGG
AAATTCTTCGGATCTCTAGCATAGTTCCAATGGGAACCACTCATGCCCCAATTAGGGATATAAAACTGAATGGATT
CCTATTGCCTCGTCACGCCCAAATCGTGCCGCTGCTTCACGCCGTTCACATGGACCCCACTCTCTGGAAGGAACCA

GAGAAATTCGACCCATCCCGATTTATCGACGGAGAAGGCAAAGTACACAAACCCGAGTATTTCTTGCCATTCGGAG
TGGGCAGGAGGATGTGCCTGGGGGAGGTTCTTGCCAGGATGGAGATCTTTTTATTCCTGTCCTCCCTGCTACATTC
TTTTGAAGTTAGTGCACCCGATGGTGACACCCTGCCAACCCTGAAGGGTATCGTCGGAGTCACCATCAGTCCTACA
CCATACAAAGTGTGCCTCAAGCCGAGAACGATTGCATGGGAGAACGAGGACGTGAACCTGCGGCCAGCAGGAAG
TCACTAGACTTCTTGAACAGTGTTCAAGAGCACGGTCAAAGAGTGGTTCCTAAGTGAGGCACAGTACTTAAGCAG
ATTCAACAATATATAAAATGGGACGTTTTCGTTCGAAACGATCCTTTATTTGATCGGCTGTGCATTTAAAGTGTTCA
TATTCACCGCCTTTAGTATTAGATATATTGTAGTACAATTGCTAGTCAGGCATGTTCATCAGTTTTACCTAGTTTTGC
AATTCCATACAAGCCATAGATTTTTACCATTTTGACTTGTCATTGTTGATTATTANTTTTTTACTTTATTCGTAACCA
TATTTGTGTGGGGTTGTGAGTGCTACTTTGTTACCGAAACGAAACCATATATTTTTAGATTTAGACTTTATTTATTGT
AAAGTAACGAACCGTGGTCGGTTTCTTATAGAAACCCTCCGTACGATATCAACAATTTATACACGCAGATTGTATCA
TCCATATTCATCATCGTAAATATAATTAACGTTGTAAATATTTTGTTCCGCAATTGTGCTTAAAACAATTTTTGTTTTT
TTTAGAGAGCAAGACTTTATATTAAGATTTGTTATTTTAATGAGTTTTAATAAAATATAAGTTAAAAATAAAAGAAA
CAGAACAAAAGACAAAAGTACTCTCCGTTGATACCACTGCTT

> RferU16_CYP12V4

AAAAATATACACGAAGCGTCCTAATATAATACGTTTAATACGTATGAGATAAAACCATCTGTTTTCGAGAACAATG
GCAACTAAACAAATGTTACGCCATGGGTTCGTGTGTCAATGTCGGAGCTATGGTTCTACTCAGATATTAAGAAAAG
CTGCCTCTCAACCAATAGCCATTAATGACGTCAACTCAAAAGATGCTTATTACAAAAAAATGGGCCAGAAGCTGGA
TGTTGATGGAAGGCCTGAGGGATGGGACCAAGCCAAACCATTTTCTTCTATACCTGGTCCAAAACCCTTACCGATA
TTGGGGAATGTGTGGAGGTTCTTTCCAGGAATTGGCGAATATCATAATATTGAACTTGTAAATTGGTATAAAGGTA
TGAAAGAAAAGTATGGCGATATCGTTTATTTTTCGGGCATACCTGGAAAGAAACCATCAGTTCTGTGCTTTAATCC
GGATGATACTGAAACAATATTTCGAAATGAGGGGGCCTGGCCGATAAGATATGGACTAAACTCTTACATCTATTAC
CAACAGAAAATGCGACATGATGTTTTTCAAGGAATAGGTGGTGTGCTGTCTACCCAGGGCGAGAAGTGGTTTGAA
AATAGATCTGTAGTCAACAAGATCTTGATGCAGCCACGTACAGTTGATTTATATGTGGACAGTATGGACAAGGTG
GCTACAAGTTTTGATTGATTAATATTCGATATTTTTCACGTAGCAGATCCTGATAATATGATTTCCAACGATTTTCAA
AATGAAATATTCAAATGGACAATGGAGTCGATGGCCCTTATAACTTACAACAAACGAATAGGCGTTTTGAATCGAA
ACCTCCAAAAGGACTCTCAAGAGCAACGATTTATCAATAGCGTTCTGTCATTATTCGATTTATCTTATAAATTGGAT
ATATTGCCATCGTTGTGGCCATACATATCAACACCCAACTGGAGAAAATTCGTTAAAGCTATGGACTTTCTCACCGA
GTTGAATCAGAAATATATCCAAGAATGTTTGGATTCGAGTGATCCAAGTATTCCGGACCACGAGAAAAGTGTGCTA
GAGAAACTGATCGAAAAAGATCGTCGAATCGCCATAACCATGGTTAATGATATGATGATTGCTGGAATAGATACG
ACTGGTAAAACCATAGCAGCTGTTTTGTATTATCTTGCCAAAAACCCCGACAAACAAGAAAAACTACGCCAAGAAC
TGAAAACGTATCTACCGGAAAAAAGTAGCCCGGTGAATAAAGACCTTACAAGAGCGTCTCCTTATTTGAAGGCTGT
CATTAAGGAAGCCATGCGACTATCACCTATAGCGATTGGAATCCTTCGGACCTCTGTCAAAGATATGGTACTGAAT
GGTTATCAAATACCAAAAGGTACTGAAGTAATTTCTATTAACATTTTACCGTCGAGAGACGACAAATATTTCATGG
ATGCCAACGAATTTATACCAGAGAGATGGCTACGAAGTACTACAGATGAATATTCGTCCAAAAACGTTCATCCATT
TGCTTCGTTTCCATTCGGTCTTGGACCACGGTCTTGTGTAGGCAAACGCTTGGCGAACATGGAGCTACAAGTTGGT
GTAGCAAAGATAATAAGAAACTTTCAGTTCTCGTGGCCTCATCCTGATGCCCAATTCGGAGCAACCTTATTATATG
GTATTCAAACA

> RferU34_CYP314A1

CTGACAAAAAGCGAGACAAAAACGGTAGCATATTTAAAGTCGAGGTTACGTGCGGGATTAACTGGGCTTGGAAG
CCACCTGTGCCCTGGTTCTCGGAAGACGTATGGGTTTTCTGATCGAAGACCGCGTCTGCCCCGTCGCCCAGAGGCT
AGCGGACACCGTTAAGGTTAACTTCGCCGCCTGTCGGGACACGTACTTCGGCCTGCCGTTTTGGAAATGGTTCCCC
ACCAGGTCCTACCGTCACCTGTGCGAGAGCGAGGCGGCTATCTACGAACTGGCGTCGGAACTCATACGGACCGCC

GACGATTCCACAAAGGAGAGCGCCGTATTCCAGTCGGTTCTCAACGCCGACATCGACGAAAGAGAGAAGAAATCC
GCCATAGTCGATTTTCTAGCCGCGGGCATCCACACCTTRAAGAACAGTCTCCTGTTCCTGYTGTATCAAGTAGCTCT
CAATCCGGAATGCCAAGAGAAAATCATTCAGGATGATTCAAAGGCTTACTTGAAGGCATGCTCCATGGAGACCTTC
CGCATCACACCC

> RferU37_CYP12V3

AAAAGAAGAAGTAGGTATCAACGCAGAGTACTTTTGTTCTTTGTTCTGTTTCTATTTGCTTTGCGAGGCCGTAGAAA
ATGGCCGGTAAAAACAATATTCTATCACGAAAAAGCGTTTGGTACCAATGCCGTAGCTATGTTTCCACACAGCCAT
TTAAAAAAGCAGTTGCTGCGCAACAAGTTACTGTCAACGGTATCAACACTAAAGATATTTACTATAAGAAGATCGG
CAATAAACTTGAAAACGATGACAAACCAGCTGGATGGAATGAGGCAAAACCATTTTCCATGATACCAGGACCTAA
ACCAATTCCAATTTTGGGAAATATATGGAGATTTTTTCCGGGCATTGGAGAATATCATAATTTAGGGATTGTCGATT
TGCATAAAAAAATAACTGAAACCTATGGTGAAATAGCCTATTTTTCGGGTATATTTGGAAAGAAACCAATGGTATT
TTGCTACAATCCCGATGCAATGGAAACGATTTCCAGGAACGAAGGTGCGTGGCCAGTTAGATACGGAATGAATGC
CTTCGTTTATTATCAGAAAAATATACGTAAAGATGTGTTTCAAGGGATCGGTGGAGTTCTTTCTACACAAGGGGAG
GAATGGTTCAAAATTAGGTCGGCTGTGAATAAAATCCTGATGCAACCACGTACAGCTGAAATGTATGTGGGCAGT
ATGGATAATGTGGCGAATACTCTGGTTGATAATATTCGATATTTTTCAAGAGCAGATCCTGACAATCTAATGCCCA
GTGATTTTCAAAATGAATTATTTAAATGGACAATGGAATCGATGGCAGTTATAGCGTATAATAAACGAATTGGTGT
TTTAGATCGAAATTTGGATGTAAACTCCAAACAACAACAGTTCATTAACTCTGTATTAACAATGTTCGATATGTCTT
ACAAAATGGATGTCTTGCCGTCGTTATGGCCATATATTTCAACTCGAAATTGGAGGAAATTTGTGAACATCATGGA
CACTCTCACAGAGTTAAATCAAAAGTTTATCAAAGAATGTCTGGAATCGATCAATCCAGACTCAAACATTCCCGACC
ATGAGAGAAGTGTTCTTGAGAGATTGCTTAAAACAGATCGTCGAATTGCCATAACAATGACTAATGATATGCTCAT
TGCGGGAATCGATACGACCGGTAAGACCATGGCTTCTGCTTTATATTTTCTCGCGAAAAATCCAGAAAAGCAAGAA
AAACTACGACAAGAGCTGAAAACTTATTTACCAAATAAAGAGAGTGTGGTTAATCAAGATATGATAAGGGCATCG
CCTTATTTGAAGGCTGTTATCAAAGAAACCACAAGGTTGGCACCTATAGCCATTGGAAATCTGCGGTCTACTGTGA
AGGATTTAGTGTTGAGTGGATATCAAATACCAAAAGATACTGAAGTTGTCTCCATCAACATATTGTCGTCGATAGA
TGACAAATATTTTCTTAAAGCTCAGGAATTCGTGCCGGAAAGATGGCTTCGTACTACTGCGGATGAGTTTTCTCATA
AGAACGTTCACCCCTTTGCTTCCGTTCCGTTTGGTTTCGGTCCTAGATCATGTATAGGAAAGCGACTTGCAAATTTG
GAACTGGAAGTTGGCTTAGCAAAAATGATAAGGAATTTTCAAATTTCCTGGCCACATGCTGATGCTAAGTTCGGTG
CAACTTTATTGTATGGTATTCAATCTCCCTTAAGATTCAAAGTTGTTGAAGTTGATAATTAATATCGAATTCTGATTT
ATTTCTGTTAGTGTTAGTTATTACTGGAGGACTTCGTTAATAGAAATAAAGCCATGAAAACTAAAATACACGGCAA
TTGTCGTATAATTTATAAACAATATTCAAAGTTATATATAAGAGTGGTTTGGTGAATCCTGTAATGTTAACAATCTTT
ATGGAAGCGCTTAGTCATTATATAGTTACTTAGTGATTATACACTTTTTTTCGCAGAATTTAATAGGCTCAAGAACC
AGAACCTGACTAATGGTCAGAAGCCAAATGTTTATATTTATATTAAATATAGAAATTATTTTCAACAAAAAAAAAA
GAAAAAAAAAACAAAAAGTACTCTGCGTTGATACCACTGCTT

> RferU44_CYP301A1

GTTCTAGGGCCAAAACCCATCCCGTTGCTTGGCAATACTTGGCGATTGCTACCCTTTATTGGACAATACGATATATC
CGACGTTGCCAAATTGTCACAATTATTCTACAAGGAATACGGAAAAATCGTAAAAACTTTCCGGTCTTGTTGGAAG
ACCCGATCTGTTGTTTATTTATGATGCAGATGAAATAGAAAAGATTTATAGAAATGAAGGACCGACTCCTTTCAGA
CCTTCCATGCCATGTTTGGTCAAATATAAAAGTGAAGTTAGAAGAGATTTTTTTGGGGAAGAAGGTGGAGTAGTT
GGAGTGCATGGGGAGCCTTGGAAGACATTTAGAACCAAAGTACAAAAACCAATATTACAATTAAAAACTGTTAGG
AAATATGTCGAACCTATCGAAGCTGTTACGAGCGATTTCTTAGCTAGGATGATCGAAATTAGAGATGAGAATCAA
GAAATGCCGGCGGATTTTGACAATGAAATTCACAAATGGGCCTTGGAATGTATAGGCAGAGTATCGCTGGATGTT
AGATTAGGTTGTTTAGATCCAAACTTAAAGCACGATTCCGAAGCTTTAAAAATTATCGAGGCCGCCAAATACGCGT

TAAGGAACATCGCAATTTTGGAGTTGAGGTTTCCGTTTTGGAGATATTTCCCGACAACATTATGGACCAGATATGT
CAAGAACATGGATTATTTTGTCGAAATATGCATGAGACATATCGATGAGGCGATGGATCGTTTAAAACATAAAACT
GTAAAAGACGAAAAGGATTTATCCTTGGTAGAGAGGATACTGGCGAGCGAACCCGACCCCAAAACGGCTTATAAA
CTGGCTTTAGATCTAATCTTAGTTGGAATCGATACAATCTCGATGGCGGTTTGTTCCATATTGTATCAATTAGCAAT
CAGACCAGAACAACAAGAAAAAATGTATAGCAGAACTCGTCCGGATTTATGCCGGACCATAAACGAACCGTTGAC
AGTTGAGAAACTTAATCAGACTGACCTTTTTAAAAGGCATTTGTGAAAGAAGTCTTCAGGATGTACTCCACAGTAA
TAGGAAATGGAAGAACCCTCCAAGAAGACACGGTACTTCTAGGATACCACGTACCCAAAGGGGTTCAGGTAGTAT
TTCCGACCTTGGTAACCGGCAGCATGAGCGAGTACGTTTCGAACCCAACTGAATTCCTTCCGGAGAGATGGATCAA
ACAGGGATCCGGCCAGGACAAAATCCATCCATTTGCATCGTTGCCATATGGATATGGGGCGCGTATGTGTCTTGGT
AGACGATTCGCTGATCTCGAAATACAAGTGCTCTTAGCTAAATTGATCAGGTCTTACAAACTGGAGTTCCTACACG
ACCCACTGGAATACAAGGTCACTTTCATGTACGCCCCAGACGGTGAACTTAAGTTTAAAGTAACAGAAAGACCCTA
GAATTCTTCTTCATACGAGAGGAGACTATTTTTACTCTGATAATTATTTTAAAGTATACGTTTTTCACACGAAAAGGT
ACTTATGTAAGATCGC

> RferU51_CYP315A1

GTTTACGGTAATCCGACTTAGAAGTTGTTGAGTATTTAACAGTTTTGGAAATTCCTGTTTATGATGGCAACGATTCG
AAGCAGCAGTTTCAACAGCATTACATAATGTGGCGAACTTAGTCGAACTATTACTAAACAATTATGTAAGAGAAAA
CGAAGGACTGTTGGGGAAATTAAGAGGAAAAATGACAACAAACGAATTATCTCGTATCGTAACGGATCTTATATT
GGCTGCCGGCGACACAACGGCGTATTCAATGGAATGGACTTTATATCTGAATTGGAAAAACCTAGACGTCCAAAA
GAAACTCCGAGAACAAATTGCAGAGGATGAAAAGAAGAGTCAGTGCTGGACCTCTAACCTGTATTTAAAAAACAC
TATTAAGGAATCTCTGCGATTATACCCAGTAGCTCCATTTTTAACTCGAATTCTACCGAAACCTGCAACGATCCAAG
GATATTATATGCCGGCAGGTTCCGTTCTGATAATGTCGATTTTCTCGACAGGCCGAGATGCCATCAATTTTCCGAAT
CCACTAAAATTTGACCCCGACAGATGGATGAGAGACGATAACAATGGCGTCGCTGCCCGATCTGCATCTATACCGT
TTGCAATAGGTTCTAGGTCCTGTATAGGCAAGAAACTAGCAGAAAAACAACTTCAAATAACTTTAGGTGAACTTGT
GAGGAATTTTCATGTTACTGTCGATAACTCGGAAGACGTTGACGTTATATTGGAAATGGTTGCCATACCGTCGGAG
AAAATTAAGTTTCAATTTTCCAATTTGGAATAACTAGAAGATATTACGTACACCCGTATATTAAGAGAATATCCAAC
ATATACGCCAATCCCTTTGTATTTTCTCACCAGTCAAGAAGTAATAATGTTACTATACGACAAAAAGGAAAGCTTCT
AATATATTAAAAAGTAAATTATTTTTCGAGAATAC

> RferU32_CYP315A1

GATGGATGGAACTCCGTTACTCGATCACTTCCCGTAAATAGAAAAGGACGGATGCTGGCTCCAGTCTCTTTTACAA
TCACCGAAAGTGAGTGCCAGTGTCCGTTCTAACAACATTACTGGAAATTATTGTGGGGGCGTCTCGATATCAGATG
TACAGGATCGTTCATTACAAGGCAACAGTTCCATTTCCTCGACATGGATAACACGGATTTTCTTCATTAAGCGGTAC
AAAATTTAACAGTTACCAGACTTGGCAAGGTGAAATGTTCTGTATGAAATGTGTGGTGCGTTGTGTTAGGGCGGA
TATATTACCCAGAGGTGGTGCTTTATCGAGAATAGCTAGACAATCAAGTACAACGGTAACGAAGACTATTGATGAT
GTTCCAGGTCCTAAAGGGTTGCCTCTTATCGGTACTACACTATCCCTGTTGATGGCAGGTTCCACACCGAAACTTCA
TCATTATATAGATAAAAGACATTAAAGAATACGGGTCTCATTTTTAAGGAAAGTATTCGGACCAGTGACCATGTAT
ATTTATTAGTGATGCCAAAACCATCAGAGATGTGTTCGCCCACGAAGGAAAGTACCCTATCCATGTACTACCAGAA
GCTTGGACTACCTATAACACTTTACATAATGTATCAAGAGGAATATTCTTCATGGACGGCGAGGAATGGTGGCATT
TTCGTCGAATATTGAACGCAGCTCTGATGAAGGGCGACCAAGAATGGCTTGAAGATAGCTGCGTACCTGCCGTTG
AAAATATTATGCGAAGAATTCGAACGGTTTCTAGTTGTACATCATCATATCCTGATATGGAGCAGATATTGTATTCC
TGGTCATTAGAGGTAATAATATCGGTGCTAGTCGGACCACAGAATTATCTGTTAGCGAAAGATCAAATTGCCGATA
GGGTCAGACTGAGTTGGCGTCAACGTTACGTCTTATTTTTGAGACTAGTTCTAAGCTAATGTTGATTTCAAGTAAAT
TCGCTGCAAAATTCGGCATTTCCAAGGTCGGTATAAGATGGCAAACGATTCGAAGCAGCAGTTTCAACAGCATTAC

ATAATGTGGCGAACTTAGTCGAAACTATTACTAAACAATTATGTAAGAGAAAACGAAGGACTGTTGGGGAAATTA
AGAGGAAAAATGACAACAAACGAATTATCTCGTATCGTAACGGATCTTATATTGGCTGCCGGCGACACAACGGCG
TATACAATGGAATGGACTTAAAAGTATAGATTCGAAAAAACCTAGAGCAACGCAAAAGAAACAGAACAAAAGAC
AAAAGTACTCTGCGTTGATACCACTGCTT

> RferC12488_CYP49A1

CAGAGATGCGCGACGGGGTTGTAGTATCTCGTAGTTGGCGGGGGGGCGCGTAGAATTCCGAAATGTCGCGCTTT
GGTTGTGTTACAAAATGTTCTGGTTCTCCTTACGGTCCTCCGATCACGCTGTCTATAAAATTTGGCGCTGTAACTCG
CGCTATCATTCCTCGTTTAACAGGGGTGCCATAGAGAGGTAGTTGTTTTAGGAATTCTTGGATGCCGGTTTTATTTT
GAATATATTCAGTGTTAATATCCGGATTTCTTAGGTCCTCATCATGTCGGGCCACGTCCCCCTCAAAGCGATCCGCC
TATTACAAAAGGCCACGCCCGTGCGCGCAATCCACGCCCTCCAGATCCGGACCTACTCGACCTCGCCTGACCAGAG
TCCCAACCCCCGAGGGCGGACGTTCTCCACGGCTGTCGACATCCTACCGGAGGACGCGTACGAGACCCTGTGGGA
CGCGGACGACGTCGTGCAACCGTACAGCGCCATCCCCGGACCTCGGGAGTTGCCCATCATCGGGAATGCCTGGAG
ATTCGCCCCGATAATCGGTCAGTATAAGATCCAGGACCTGGATAAGGTCATGTGGTCGCTGTACGAGGACTACGG
GCGTATAGTCAAGGTAGGCGGGTTGATCGGGCATCCGGATCTTTTGTTCGTCTTCAACGGAGATGATATTCGAAA
GGTGTTCCGTAGGGAAGAGGCGATGCCTCACCGTCCGTCCATGCCCTCACTGCATTACTACAAGCAGAAACTGCAC
AAGGGCTTTTTTCACGGCAACGAAGGCGTTATCGGAGTGCACGGACCGAAGTGGGACGCCTTCAGGAAACAGGT
ACAGCAGGTCCTGCTGCAGCCCGCGACGGCCAAAAAATACGTCGAACCTCTGGACGTCATCGCCGACGACTTTCTC
GGCCTCATGGAGCACTCCCTGGACCACAACGACGAGCTGCCCGACAATTTCCTCTCCGAGATCTACAAGTGGGCCT
TGGAATCCGTCGCCAGGGTGGCTCTGAACACCCGCCTGGGATGCCTGGACGTCGAACTGCCGCGCGAGTCCGAGT
CCCAGAGGATCATCGACTCGATCAACACGTTCTTCTGGAACGTCGCCGAGGTCGAGCTCAAAATGCCCGTGTGGA
GGGTGTACCAGAACAAGGCGTTCAGGAAATACATCGGGGCCTTGGACGACTTCAAGACATTGTGCCTGAAGTACA
TCATGCAGACCGTGGAGGCCATGCAGTACAAGAACTACGACCACGCCAAAGAGGAGGATATCTCGATAGTGGAG
AGGATGCTACTGAAGACTGATAACCCTAAACTGGCGGCCGTTCTGGCCCTGGACCTGCTGCTAGTGGGCGTCGAC
ACGACGTCCATTGCTCTGGCGTCCACGGTGTATCAATTGAGTCAAAACCCGGCCAAACAACAAAAACTGTTCGAGG
AACTGAGAAGAGCCATGCCAAGGCCCGATTCCAAGATCGATGTAAAGACCTTGGAACAAGTACCGTATTTAAAAG
CCTGTATCAAAGAAACTTTAAGAATGTATCCTGTGATAATTGGTAACGGAAGATGTCTCCAATCTGACACAGTTCTC
GCGGGATATCACGTGCCCAAAGGGACTCACGTTATCTTCCCTCACTTAGTCGTAAGTAATATAGACGATTATTTCAA
AGAACCAGAACGATTTGTGCCTGAACGGTGGGTAAAACACAACTCCAGTTCGAAATGCCCTCATAATCAAGAAAA
AGCGCACCCTTTCGTGTCGCTTCCGTTTGGATACGGGCGTCGTTCTTGCTTAGGGCGTCGATTCGCCGAAATAGAG
TTATATATCATGTTGGCCAAGATTTTCAGGAAATATCAAGTAAACTTCAACTACGGGCCTCTGACGTACAAGATTAC
CCCCACTTACGTGCCGGAACAACCCCTAAAGTTTAAACTAACCCCTAGAACTGATTGATTACACTAACCCAGAGTAT
TATTAAAAGAAATATATTTTTTTCTTTATAGCGTCGTG

> RferC10039_CYP334E7

TGTTAACTCCCATTTAATTCCTATCATACCCCTATGTAATAAAAGTCGCTATAGCCTTGGCAAATTAAATATGCCGAT
TTGACATTTCGGTTATTGTCAAATAGTCAATGTTAAGTTGCGGCTTTGTTTAAAGTGTTGTTTCTGTTAAAAATGTG
GAGAATATCGAAAATACGAGCAAATGTGGTATTGGGAATTAGATATTGTGCCACCGAAGCCAAGTCCACCGCGTC
TCAGATAACTACCCCTTTGGTTGATGGTATTCCACCTCCAGCGACTGTCGTAAGAAAGGAAATACCAGTAGTGAAG
AAAATTTTAACGGAAGTTAAAGAACAAGATGGTAGAAGCAAAATTATCGAAAAATTTTGCGTCTTCGATGATGTAC
CAGGTCCGAGGAGTCTAAAATTAATTTCCAACATATGGAGTCACGTTCCCGCTTTAAGTAAGGAGTTTACTGCCGG
AGCACTTTTTCAGACTGATCGGATTTGGGNNNNNNNNNNNNNNNNNNNGCTCAGTTGGGGCGGTAACGCAAGCT
TTTTCCAAAAATTCTTTGACGTTTATGGTCCCGTGGTGAGACTGCACGGCCCTTTCGGTGGCGACGTCCTTCTTTTG
TCCCGCCCTGAACATGCCAGTATTGTTTTCCGAAACGAAGGCACGCGGCCTGTCCGGGCTTGTTTGGATTCAGTGG

AAAAATATCGTCTGGAGCATCGAAGACTGAGACAGGCAGGTCCTTTCTTGATGTCGGGTTCTGCTTGGGAAAAGA
TCCACGATTCCATCGATGATCCTATAAAGACTTCCGTAAATCGATACGCCAAACCAATGAACGAGATATGCGATGA
GTTCGTCGAACGCGTTCTGAATATTAGAAACTTACAAGACGAGGTACCTAAGAACTTTAAGGACGAGATTTTAAAA
TGGTGTCTGGAGAGTATGTGTGCCGTTACACTGAACAGAAAGTTTGGGTTTTTGGATACAACTGGTTTAAGTACCA
CATCGGACCCTGGAAGGATTTTGGATGGAGTTAATGGTGCTACTGAAGCTATCAGGAAATGTGAGTTTGGTTTCC
ATCTATGGAAATTTGTGGAGACACCGGCTTGGCGTTCTCTGGTGCGTAACTGCGACTCGATCGATTCCATTCTGAG
TAAATACGTAGAACGAGCCCAGACCGCATTACGTGAAAGGAAAGATCTGAACCCAAAGACGGCGGATTTTTCCGT
AGTCGACCGTTTGTTGCTCAACGAGGACGTTCTAATCGAAGATGCCATGACCGTCCTGCTGGACATGTTCATCATC
GGCGCCAACGCCACCGTGCACTCCGTGGCGTTTCTACTATATCACTTGGCGAAGGCTCCTAAGTGCCAAAAGAAGC
TACAACAAGAAATCGACGGGCAGACCCGCACCGAACTCACCGCAGACGATCTGAAGCGCATGCCTTATCTGCAGG
CCTGCATCAAGGAAAGCTTACGACTAGCGCCACCGATACCGATTTTGAATCGCGTACTCGCGCGAGATGCTATCAT
CCACAAGTATTTGGTACCCGAAGGCACGTACGTCCTAATCGCCACCCACTTGGCCAGCTTGCGCGAAGAGTATTTC
GAAGATGCCCGTCGCTTCCAGCCAGAACGTTGGCTCAACCAGGAAGTCGGTCGTCTCGCTCGCGATCTTCAGGAG
TTTGCTTCCATGCCCTTCGGTCACGGTCCCCGAGCTTGCCAGGCGCGCGAGCTTGCCGAGATGCAGGTGGGCTTGC
TGGTGGCCAAGGTCCTGAAGAGGTTTAACATCGAATATAATTATGGGGAGATGTCTAGCTCAAATTTGATGCTCTC
CAGCCCAACCAAACAGATCGGAAGAGCG
